# Intramolecular hydrogen bonding enforced by crystal packing and *vice versa* in nitrophenyl-, nitropyridyl-, and nitropyrimidyl-bearing carbazoles

**DOI:** 10.1039/d6cp02608g

**Published:** 2026-08-25

**Authors:** Jai-Ram Mistry, Patrick J. Kimber, Ruth M. Pollard, Glen Masterson, Lingyu Yang, Chloe R. Antonen, Stephanie Montanaro, Stefan Warrington, Simon J. Teat, Mark R. J. Elsegood, Felix Plasser, Marc K. Etherington, Iain A. Wright

**Affiliations:** a Department of Chemistry, Loughborough University Epinal Way Loughborough Leicestershire LE11 3TU UK; b School of Engineering, Physics and Mathematics, Northumbria University Ellison Place Newcastle upon Tyne NE1 8ST UK; c Advanced Light Source, Lawrence Berkeley National Laboratory Cyclotron Road Berkeley CA 94720 USA; d EaStCHEM School of Chemistry, University of Edinburgh David Brewster Road Edinburgh EH9 3FJ UK iain.wright@ed.ac.uk

## Abstract

This study concerns the synthesis, structural analysis, and optoelectronic properties of *N*-aryl and *N*-heteroaryl-9*H*-carbazoles in which any nitrogen atoms in the heteroaryl rings are positioned *ortho*- to the nitrogen atom of the carbazole. In principle, these N-atoms can display intramolecular hydrogen bonding (IHB) with the H^1/8^ protons of the carbazole. This IHB is apparent in the structures determined using X-ray crystallography, and the nitropyrimidyl compound in particular shows significant IHB stability with an almost perfectly planar structure. Here we show that when a substituent is present on the other *ortho*-site of the aryl moiety, any IHB in the crystal phase is being enforced primarily by molecular crystal packing by comparison with the calculated structure of their ground and first singlet excited state geometries in the gas phase. This is supported by the results of solution phase ^1^H-NMR spectroscopy, cyclic voltammetry, and UV-vis spectroscopy. A notable observation for the planar nitropyrimidyl compound is that it has a relatively wide HOMO–LUMO gap due to changes in the orbital distribution around the carbazole N-atom. These insights offer pathways for modulating optoelectronic behaviors in π-functional materials and highlight the impact of IHB and steric interactions in carbazole-based chromophores.

## Abstract 2

Interplay between steric hindrance and intramolecular hydrogen-bonding (IHB) in *N*-substituted donor–acceptor carbazoles is examined. This reveals that only specific derivatives maintain strong IHB with crystal packing often enforcing planarity.

## Graphical abstract

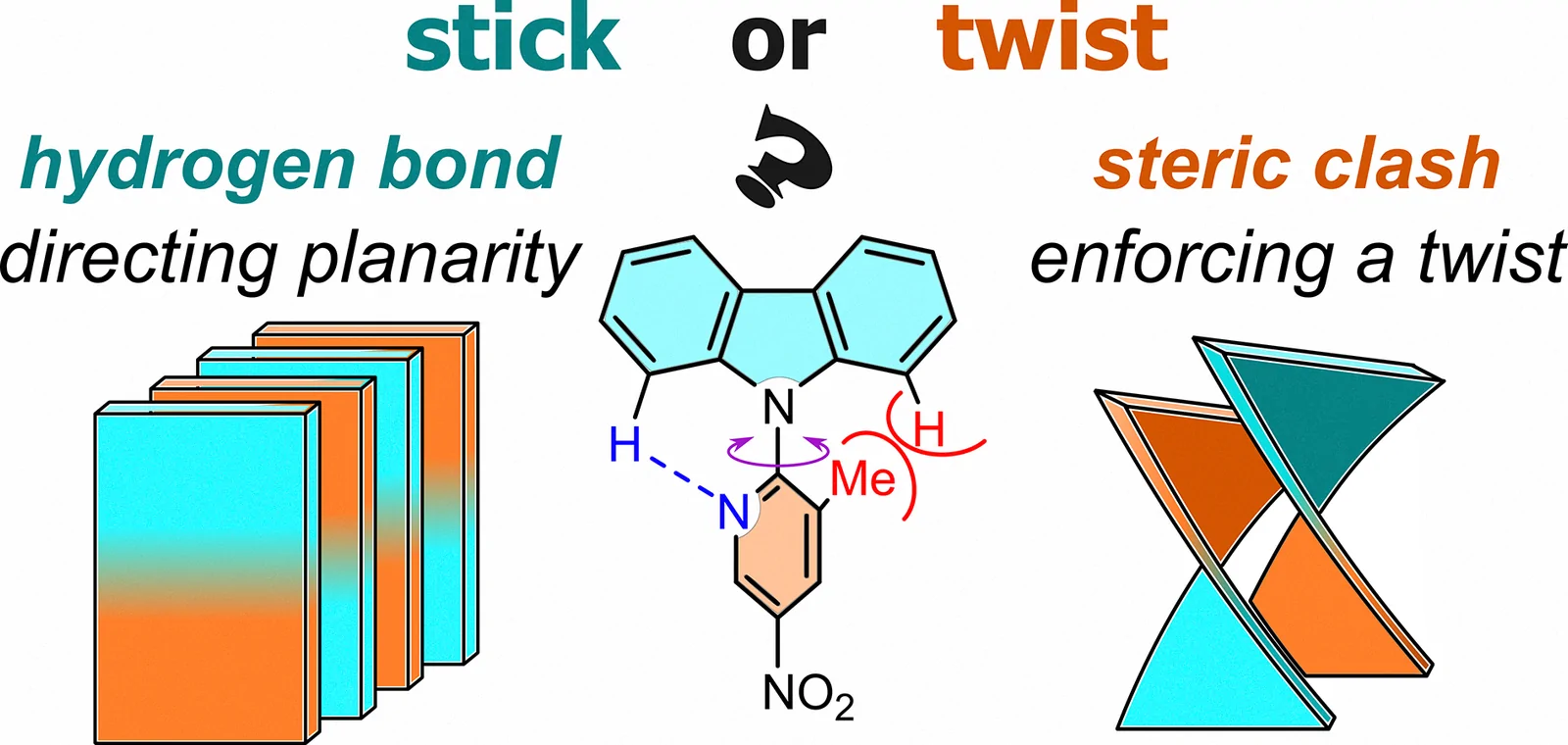

## Introduction

Carbazole is a widely employed heterocycle in π-functional organic materials chemistry, particularly pertaining to organic light-emitting diode (OLED) technologies. It can be used as the donor component in small molecules displaying emission through intramolecular charge transfer (ICT) which can be exploited towards thermally activated delayed fluorescence (TADF).^1^ Linear oligomers^2^ and polymers^3^ of carbazole also display efficient blue luminescence and good charge transport, with high triplet energies allowing them to act as efficient host materials.

*N*-Aryl-9*H*-carbazoles present opportunities for controlling the optoelectronic properties of such molecules. By appending suitable electron-withdrawing groups (EWG) to the aryl ring, a donor–acceptor structure is established and ICT can be observed. In this way, intuitive control of absorbance and emission wavelengths and redox properties of the molecule can be established. Broadly speaking, the highest occupied molecular orbital (HOMO) can be modulated by changing the substituents on the electron-rich carbazole, and the lowest unoccupied molecular orbital (LUMO) can be tuned by varying the substituents on the electron-poor aryl ring.

Alongside electronic effects, structural factors will also contribute to the properties of these molecules. They have a non-planar geometry typically with an interplanar angle along the bond between the carbazole nitrogen atom (N_Cbz_) and the carbon atom of the aryl ring it is bonded to (C_Ar_) of ∠N_Cbz_–C_Ar_ = ∼50°. This twist arises from steric clash between the H^1^ and H^8^ hydrogen atoms of carbazole and the hydrogen atoms (or other substituents) in the 2′- and 6′-position of the aryl ring. Derivatives with EWGs such as cyano-,^4^ ketone,^5^ and ester-^6^ substituents at the *para*-position of the aryl ring are emissive and show significant solvatochromism in both absorbance and emission. For the cyano- and ester-bearing compounds, this has been attributed to the formation of twisted intramolecular charge transfer^7^ (TICT) states where ∠N_Cbz_–C_Ar_ is completely orthogonal in the ICT state. Studies from Kapturkiewicz^5,8^ and Zachariasse^9^ and their co-workers have suggested much more limited variation of ∠N_Cbz_–C_Ar_ between the S_0_ and ICT states in compounds bearing *para*-cyano groups, while work by Lifshits *et al.* on the ester-bearing derivatives suggests an initial local excitation over the carbazole occurs, followed by sequential formation of the orthogonal TICT state, then relaxation back to S_0_.^6^

**Fig. 1:**
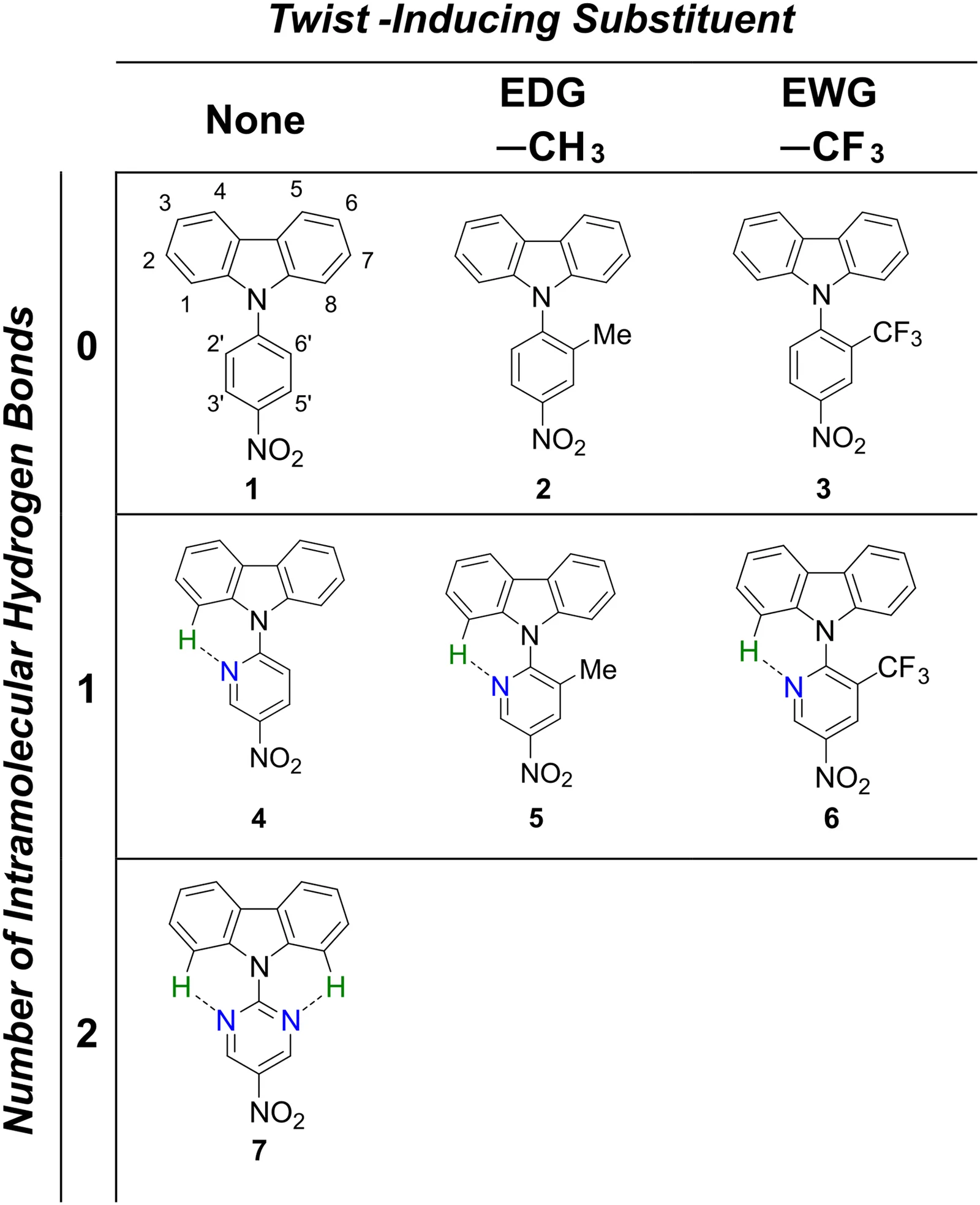
Structures of compounds 1–7 synthesised and studied in this work with the numbering convention employed shown on molecule 1. Possible intramolecular hydrogen bonds are shown as dashed bonds between the hydrogen bond donor hydrogen atoms (green) and the hydrogen bond acceptor nitrogen atoms (blue). EWG = electron withdrawing group; EDG = electron donating group.

We have devised a series of compounds 1–7 (Fig. 1) which let us examine the interplay between intramolecular hydrogen bonding (IHB), and steric hindrance imposed by *ortho*-substituents on the structural and optoelectronic properties of the molecules. The impact of whether the *ortho*-substituent is an EWG or an electron-donating group (EDG) is also explored. The synthesis of 1–7 is presented including three distinct approaches to compound 7. Differences observed between their X-ray determined molecular crystal structures and the molecular structures predicted by computational methods in the gas phase in the ground and first singlet excited states are discussed, alongside their nuclear magnetic resonance (NMR) spectroscopy, cyclic voltammetry, and steady-state absorbance and emission spectroscopy.

## Results and discussion

### Design and synthesis

4-Nitrophenyl, 3-nitropyridyl, and 5-nitropyrimidyl were selected as the aryl/heteroaryl rings to be bound to carbazole on the basis that the strongly electron-withdrawing –NO_2_ group would ensure an ICT transition would be observed. Compound 1 is a known ICT chromophore and has also previously been shown to demonstrate aggregation induced emission (AIE).^10^

Methyl- and trifluoromethyl-groups were selected as the sterically incumbent EDG and EWG respectively. The rotational barrier presented by the –CF_3_ group is notably greater than that of a –CH_3_ group,^11^ therefore larger twist angles and/or greater levels of distortion imposed by steric hindrance may be expected for those compounds bearing –CF_3_ substituents.

**Scheme 1:**
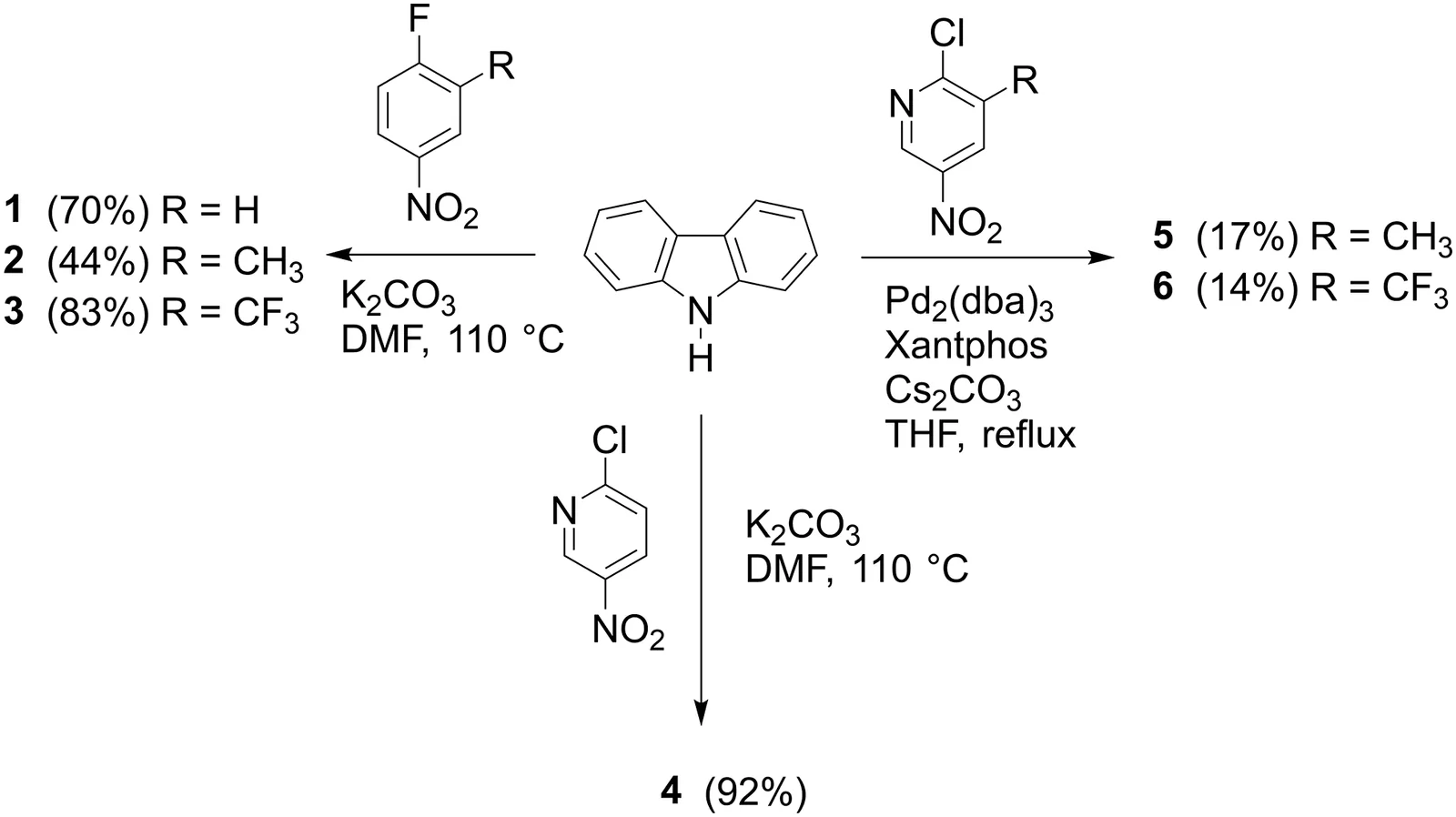
Synthesis of compounds 1–6.

Detailed synthetic procedures are provided in the SI. The synthesis of 1–6 is shown in Scheme 1. Compounds 1–4 were synthesised through simple nucleophilic aromatic substitution (S_N_Ar) reactions. Commercial carbazole was dissolved in *N*,*N*-dimethylformamide (DMF) and treated with K_2_CO_3_ prior to the addition of: 4-fluoronitrobenzene to produce 1; 2-fluoro-5-nitrotoluene to produce 2; or 2-fluoro-5-nitrobenzotrifluoride to produce 3. We note that in our hands the melting point measured for 3 was considerably higher (138–139 °C) than the previously reported value (74–76 °C).^12^ Compound 4 was synthesised in a similar fashion but NaH was required to deprotonate the carbazole in DMF prior to addition of the 2-chloro-5-nitropyridine electrophile.^13^ The presence of –CH_3_ and –CF_3_ groups *ortho*- to the site of substitution in 5 and 6 required a palladium-catalysed cross-coupling approach to be employed. Pd_2_(dba)_3_ in conjunction with Xantphos as the supporting ligand and Cs_2_CO_3_ as base in refluxing tetrahydrofuran (THF) worked in both cases but was poorly yielding.

**Scheme 2:**
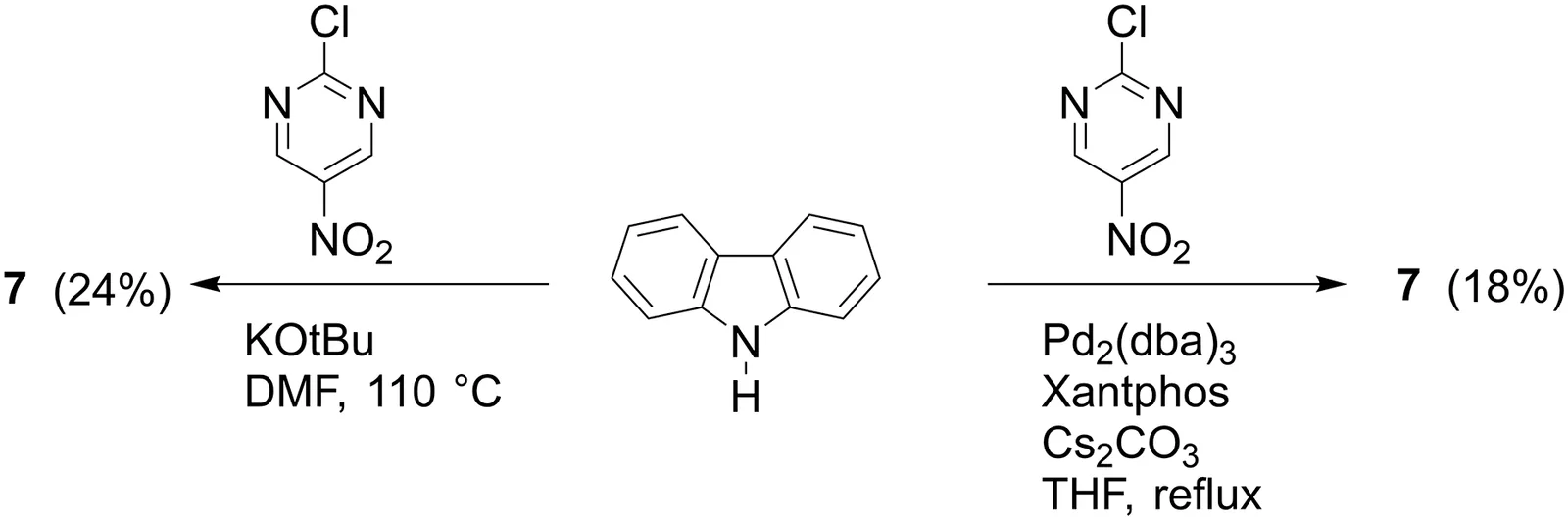
Initial synthetic routes towards compound 7.

The synthesis of 7 was surprisingly challenging and three approaches were ultimately established. First, approaches using carbazole itself were employed (Scheme 2). Initially, an S_N_Ar reaction between carbazole and 2-chloro-5-nitropyrimidine was attempted using the conditions employed for 1–4 but this reaction failed. Subsequent testing revealed that 2-chloro-5-nitropyrimidine not only degrades rapidly in the presence of a large variety of bases in THF or DMF solvent including K_2_CO_3_, *N*,*N*-diisopropylethylamine, KF, and NaH, but also decomposes spontaneously in acetonitrile in the absence of base. Ultimately, the use of DMF as the solvent and KO*t*Bu as base successfully provided 7 in 24% yield. Employing the same cross-coupling conditions as used for 5 and 6 also successfully produced 7, albeit in an even lower yield of 18%.

**Scheme 3:**
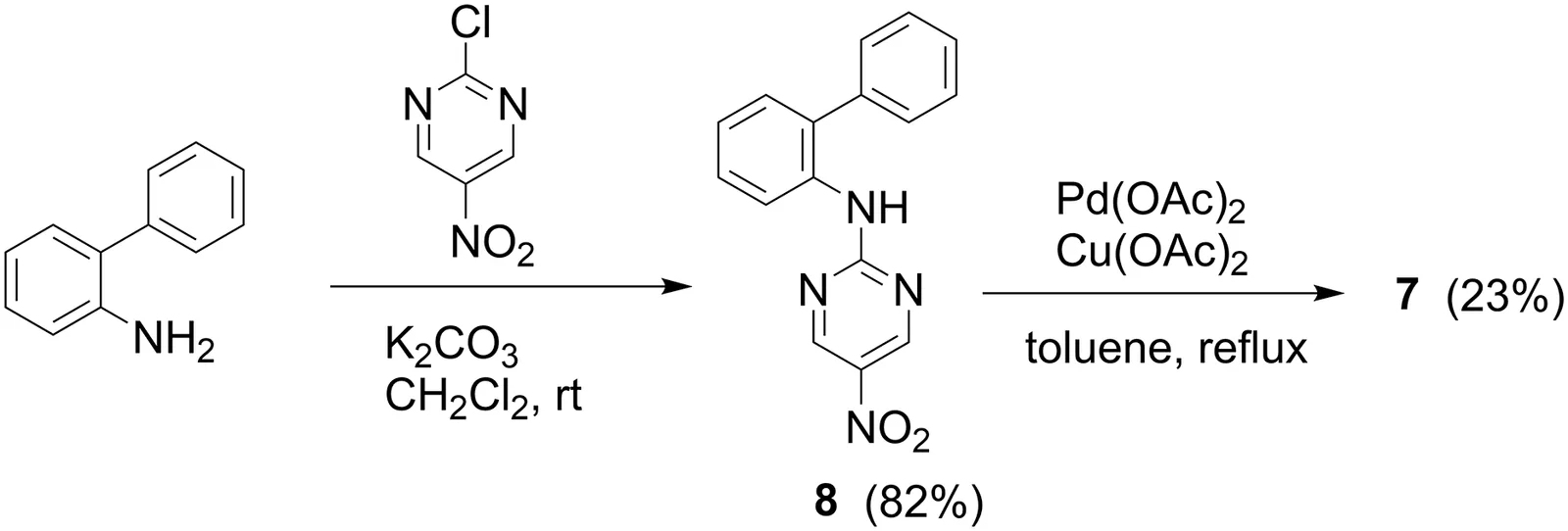
Stepwise synthetic route adopted for compound 7.

As the yields of reactions to make 7 directly from carbazole were consistently poor, a stepwise strategy was devised (Scheme 3). By using a primary amine as the nucleophile and returning to an S_N_Ar methodology, it was anticipated that an apolar solvent could be employed to form the required N_Cbz_–C bond. Gratifyingly, 2-aminobiphenyl and 2-chloro-5-nitropyrimidine reacted slowly to produce secondary amine 8 at room temperature in CH_2_Cl_2_, with K_2_CO_3_ present to mop up the HCl by-product. Oxidative cyclisation of 9 was then completed using a combination of Pd(OAc)_2_ and Cu(OAc)_2_ in refluxing toluene to produce 7 in a yield of 23%.

### X-Ray crystallography

Single crystals suitable for molecular structure determination by X-ray crystallography were obtained for compounds 1–7. The diffraction pattern from the single crystals obtained for 2 were identical to that obtained for CCDC deposition number 1008519.^14^ Focus is given primarily to the structures of the individual molecules within the asymmetric unit, with additional packing diagrams and descriptions available in Fig. S25–S37. Primary structural parameters discussed are summarised in Table 1. The interplanar angle ∠N_Cbz_–C_Ar_ was measured between two planes defined by the central five-membered C_4_N ring of carbazole and the aryl/heteroaryl ring respectively. Similarly, ∠C_Ar_–NO_2_ is the interplanar angle between two planes defined by the aryl/heteroaryl ring and the –NO_2_ group. Any systematic variations in ∠C_Ar_–NO_2_ when comparing between the crystal structures are not obvious.

**Table 1:** Selected structural parameters for 1–7

	∠N_Cbz_–C_Ar_^a^ (°)	N_Cbz_–C_Ar_ (Å)	∠C_Ar_–NO_2_^b^ (°)	C_Ar_–NO_2_ (Å)	H_Cbz_⋯N_Ar_ (Å)
	Molecular structure from X-Ray crystallography				
1	49.72(4)	1.4154(14)	9.980(18)	1.4706(14)	—
2	57.70(3) (65.41(3))^c^	1.4198(11) (1.4178(11))	4.05(6) (0.88(8))	1.4660(12) (1.4671(12))	—
3	68.61(4) (80.91(4))	1.4242(16) (1.4279(16))	4.54(13) (8.56(10))	1.4717(17) (1.4746(17))	—
4	34.12(8)	1.408(5)	8.2(3)	1.452(5)	2.50
5	51.38(4)	1.4096(16)	2.90(17)	1.4613(18)	2.571(19)
6	51.97(2) (53.77(2))	1.3982(10) (1.3990(10))	10.98(6) (12.36(7))	1.4622(10) (1.4636(11))	2.694(13) (2.656(13))
7	1.58(7)	1.3872(16)	6.7(2)	1.4486(17)	2.293(15)/2.313(15)^e^
	Ground state (S_0_) molecular structure from *ab initio* calculations^d^				
1	52.15	1.406	13.43	1.473	—
2	68.21	1.412	8.51	1.475	—
3	89.87	1.412	1.63	1.479	—
4	39.29	1.397	11.16	1.469	2.464
5	57.08	1.405	11.11	1.471	2.707
6	88.45	1.408	10.79	1.475	3.378/3.325^e^
7	0	1.383	0	1.463	2.254
	First excited singlet state (S_1_) molecular structure from *ab initio* calculations^d^				
1	54.76	1.407	0.91	1.369	
2	71.50	1.412	0.70	1.370	
3	89.97	1.411	0.04	1.369	
4	41.83	1.400	0.97	1.366	2.495
5	59.84	1.406	1.19	1.367	2.753
6	84.71	1.450	1.23	1.420	2.877
7	0	1.478	0	1.402	2.199

a ∠N_Cbz_–C_Ar_ is the interplanar angle between the two planes defined by the aryl/heteroaryl ring and the –NO_2_ group.

b ∠C_Ar_–NO_2_ is the interplanar angle between the two planes defined by the aryl/heteroaryl ring and the –NO_2_ group.

c For the compounds which have two molecules in their asymmetric unit the numbers in parenthesis are representative of the molecule with the more twisted structure.

d Calculated at the MP2/def2-SVP level.

e The large ∠N_Cbz_–C_Ar_ renders H^1^ and H^8^ similarly distant from N_Cbz_ so the values for both H atoms are shown.

### 1–3

**Fig. 2:**
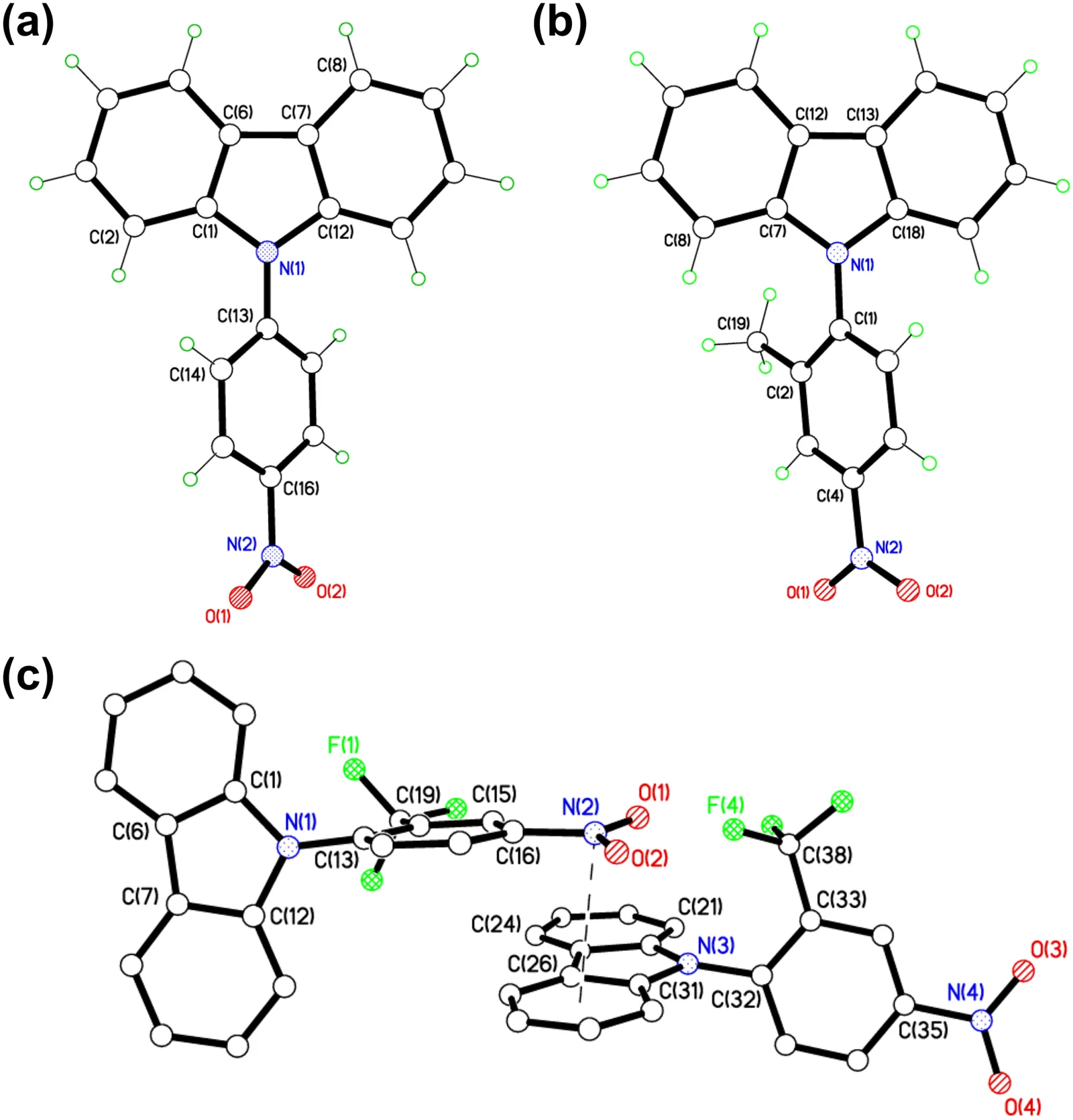
X-ray determined molecular crystal structures of (a) 1 (asymmetric unit), (b) 2 (one of two similar molecules shown), and (c) 3 (asymmetric unit).

The structures of the asymmetric units for 1–3 are shown in Fig. 2. The asymmetric unit of 1 contains a single molecule with a twist angle ∠N_Cbz_–C_A_ = 49.72(14)° imposed by H^1/8^⋯H^2′/6′^ interactions. The asymmetric units for 2 and 3 both contain two molecules. In both cases one molecule has a larger ∠N_Cbz_–C_Ar_ than the other. In 3, the values of ∠N_Cbz_–C_Ar_ are larger than those observed for 2 and some deviation away from planarity of the N_Cbz_ atom is observed. The N_Cbz_–C_Ar_ bond length increases only slightly when steric hindrance is introduced, and to a greater extent for 3 than 2. These differences are assigned primarily to the larger size of the –CF_3_ group, though may also be influenced by its electron-withdrawing strength.

### 4–6

With the introduction of a pyridyl- rather than phenyl- ring, to the system, any influences on the molecular conformation arising from IHB might be identified. The strength of any IHB will be modulated by the precise orientation of the hydrogen bond donor and acceptor atoms, and the relative acidities of the hydrogen atoms involved.^15^ Results from a study by Avendaño *et al.* provide evidence for this point very well.^16^

**Fig. 3:**
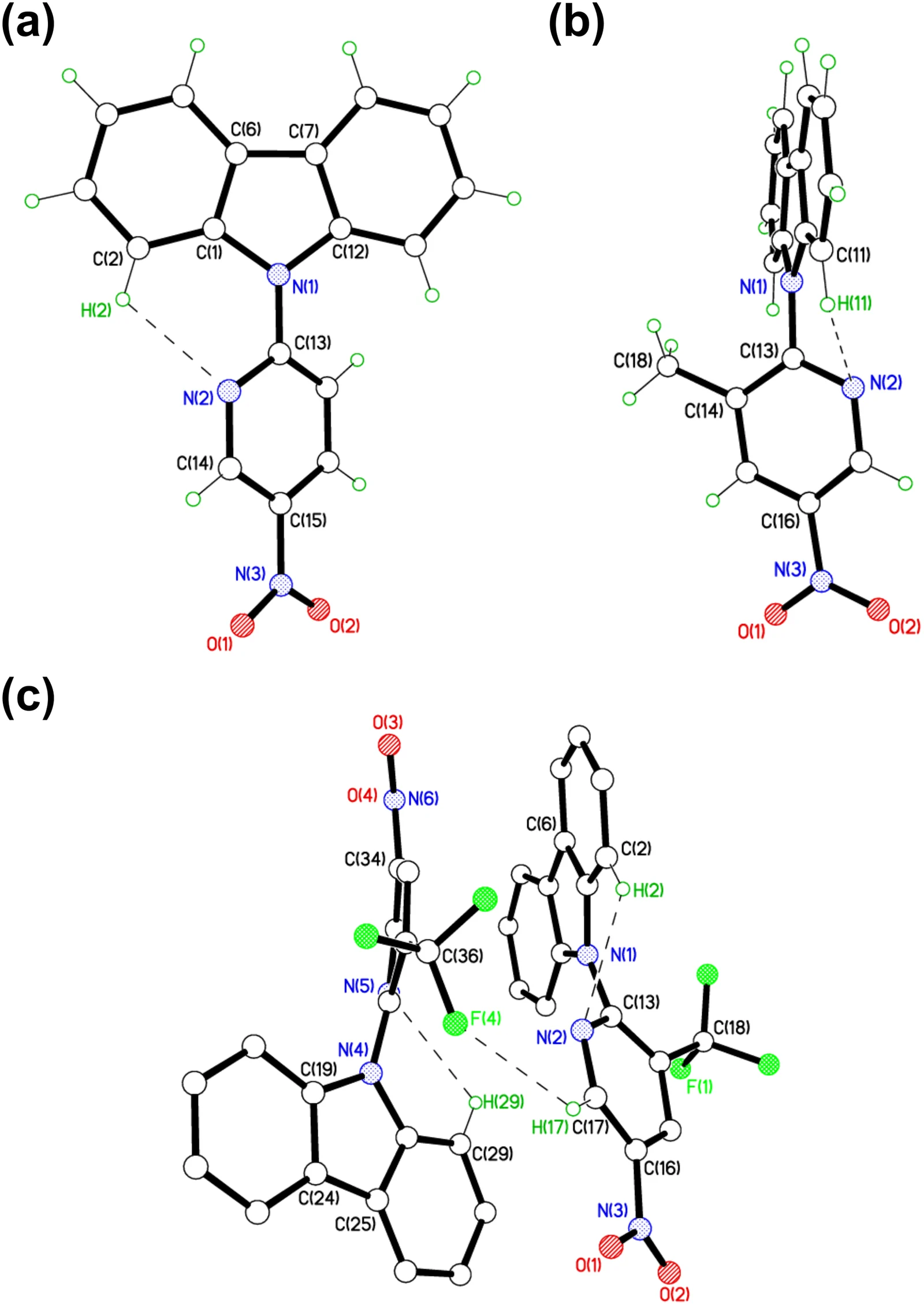
X-ray determined molecular crystal structures of the asymmetric units for (a) 4, (b) 5, and (c) 6.

The asymmetric units of 4 and 5 (Fig. 3a and b) both contain one molecule. 4 displays an intramolecular contact between a C–H atom of the carbazole (H(2) in Fig. 3a) and the N atom of the pyridine ring of C–H_Cbz_⋯N_Ar_ = 2.50 Å. This distance falls within the range at which some hydrogen bonding would be expected. We note that the space group (*P*2_1_2_1_2_1_) is non-centrosymmetric, but due to the lack of a heavy atom there is, at best, only a vague indication of the handedness, as exemplified by the large standard uncertainty on the Flack parameter. 5 has a slightly longer C–H_Cbz_⋯N_Ar_ = 2.571(19) Å. This interaction is longer than that for 4 because the –CH_3_ group has enforced a larger ∠N_Cbz_–C_Ar_ = 51.38(4)°. Similarly, due to the influence of the bulkier –CF_3_ group, the two molecules in the asymmetric unit of 6 display even longer C–H_Cbz_⋯N_Ar_ > 2.656(13) Å. In 6 (Fig. 3c), the pyridyl rings themselves are not entirely planar and are somewhat distorted. This is seemingly driven by repulsion between the –CF_3_ groups with the N atom of the carbazole, which has adopted a *gauche* conformation with respect to one of the F atoms. The two interlocked molecules in the unit cell have some weak intermolecular connections between them, and the pairs of molecules pack further into a layer structure. 6 adopts the same non-centro-symmetric space group as 4, but the absolute structure could not be determined reliably due to the lack of an anomalous scatter.

### 7

**Fig. 4:**
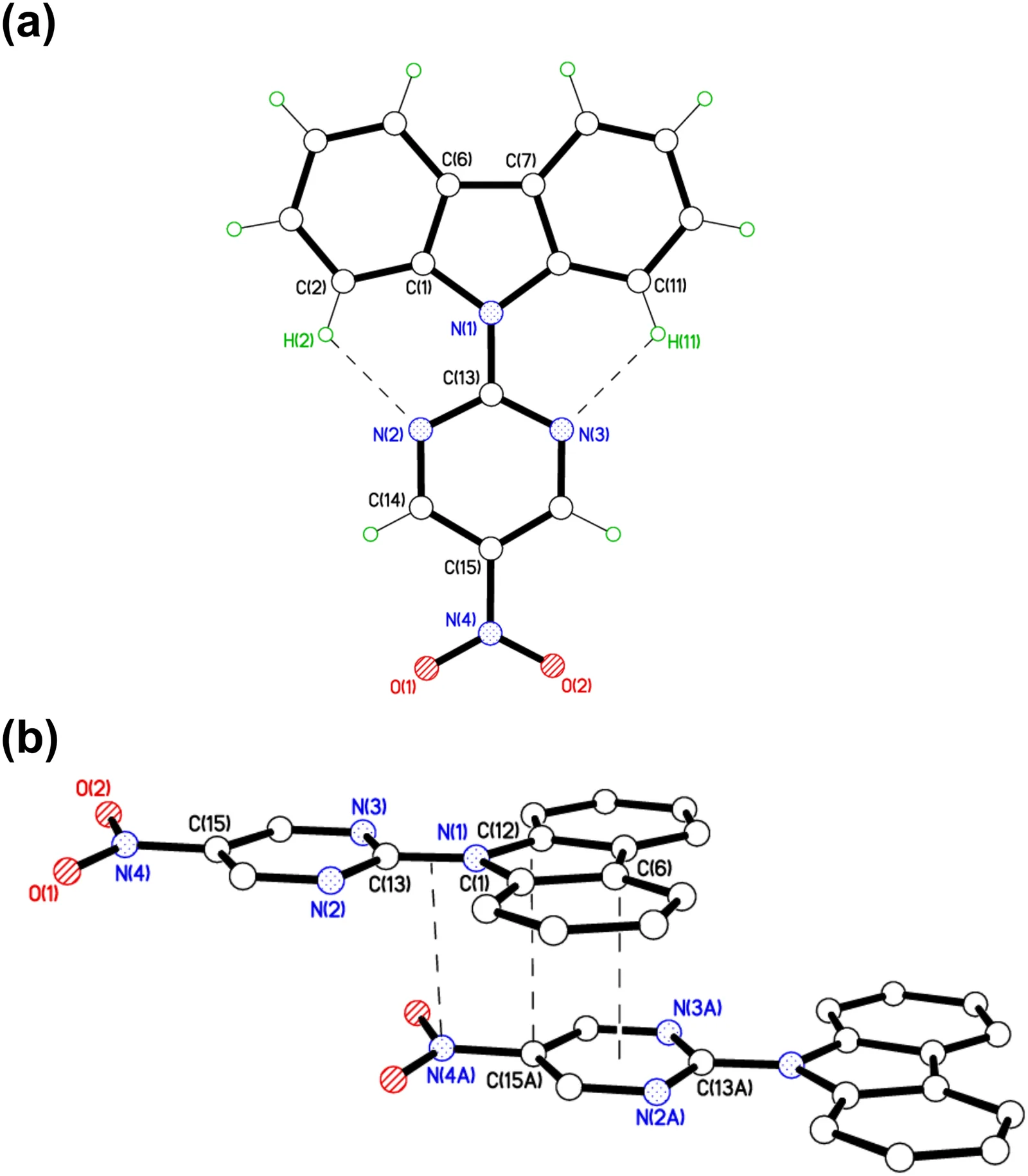
(a) The asymmetric unit of the X-ray determined molecular crystal structure for 7, and (b) a side-view highlighting the almost perfectly planar structure of 7 and its tight intermolecular packing.

Compound 7 (Fig. 4) has one molecule in the asymmetric unit. Between the ring systems ∠N_Cbz_–C_Ar_ = 1.58(7)° and the interplanar twist angle between the pyrimidine and the NO_2_ group is also small at ∠C_Ar_–NO_2_ = 6.7(2)° therefore the molecule is almost entirely planar. These flat molecules adopt a typical monoclinic herring-bone pattern with significant π⋯π stacking. The molecules are slanted or off-set, stacking parallel to *b* with ring-to-ring separations as close as 3.265 Å. This is rather shorter than the separation between planes in graphite.^17^ Adjacent stacks are connected *via* some very weak intermolecular interactions involving the oxygen atoms of the –NO_2_ groups.

When considering the influence of IHB, it is most pertinent to compare compounds 1, 4, and 7. When the phenyl ring in 1 is replaced by the pyridyl ring of 4, the influence of the N atom as a hydrogen bond donor causes ∠N_Cbz_–C_Ar_ to drop by 30% (15°). This angle tightens even more dramatically by a further 95% (with respect to 4) in 7 as the second intramolecular hydrogen bond is established. The lengths of N_Cbz_–C_Ar_ (and C_Ar_–NO_2_) follow a similar trend, getting steadily shorter as N atoms are introduced into the ring of the nitroaryl group. A contributing factor to the shortening of N_Cbz_–C_Ar_ is that the overall electron-acceptor strength of the nitroaryl groups is also increasing as electronegative N atoms are introduced to the ring system.

Finally, we note that a combination of packing interactions and the presence of IHB have powerfully counteracted the steric demands of the –CF_3_ group in 6, even to the point of causing the pyridine ring itself to buckle slightly. This has led to both molecules in the asymmetric unit of 6 having a ∠N_Cbz_–C_Ar_ which is comparable with that of 5, and actually tighter than that observed for 3.

### Computational ground and excited state structures

Ground and excited state geometries for 1–7 were obtained using the *ab initio* MP2 and ADC(2) methods with the def2-SVP basis set, as implemented in Turbomole 7.4.^18–21^ The important structural parameters are included in Table 1 and the calculated structures shown in Fig. S40. The calculated HOMO and LUMO distributions of S_0_ are characteristic of donor–acceptor systems with the HOMO distributed entirely over the carbazole and the LUMO over the nitroaryl group (Fig. S41).

The experimentally determined and computationally predicted geometries for the compounds without –CH_3_ or –CF_3_ substituents 1, 4, and 7 are in close agreement. The calculated H_Cbz_⋯N_Ar_ IHB lengths for 4 and 7 agree to within 0.05 Å. More significant differences can be observed for compounds 2, 3, 5, and 6. Much larger ∠N_Cbz_–C_Ar_ are observed in all cases. This is most extreme for the –CF_3_-bearing compounds 3 and 6 where the two ring systems are 21° and 36° more twisted in the gas phase, and so becoming essentially perpendicular to each other. This large change in ∠N_Cbz_–C_Ar_ has an immediate impact on whether IHB can occur at all, with any IHB in 5 and 6 either severely weakened or entirely broken. Unlike the crystal structure, the pyridine ring in the calculated structure of 6 is undistorted, instead, the entire carbazole ring-system is forced by the sterically demanding –CF_3_ group to hinge towards the N-atom of the pyridine. From these comparisons it seems that for the substituted molecules 2, 3, 5, and 6 any apparent IHB is being enforced by the crystal packing rather than being an intrinsic part of the molecular structure.

The *ab initio* calculations were extended to predict the geometries of the first singlet excited states which were revealed to be very similar to the S_0_ geometries. The primary differences arise from decreases in both ∠C_Ar_–NO_2_ and C_Ar_–NO_2_. Both reflecting a planarization and enhanced conjugation of the nitro group with respect to the aromatic ring. Variations of the N_Cbz_–C_Ar_ bond are negligible.

### ^1^H NMR spectroscopy

**Fig. 5:**
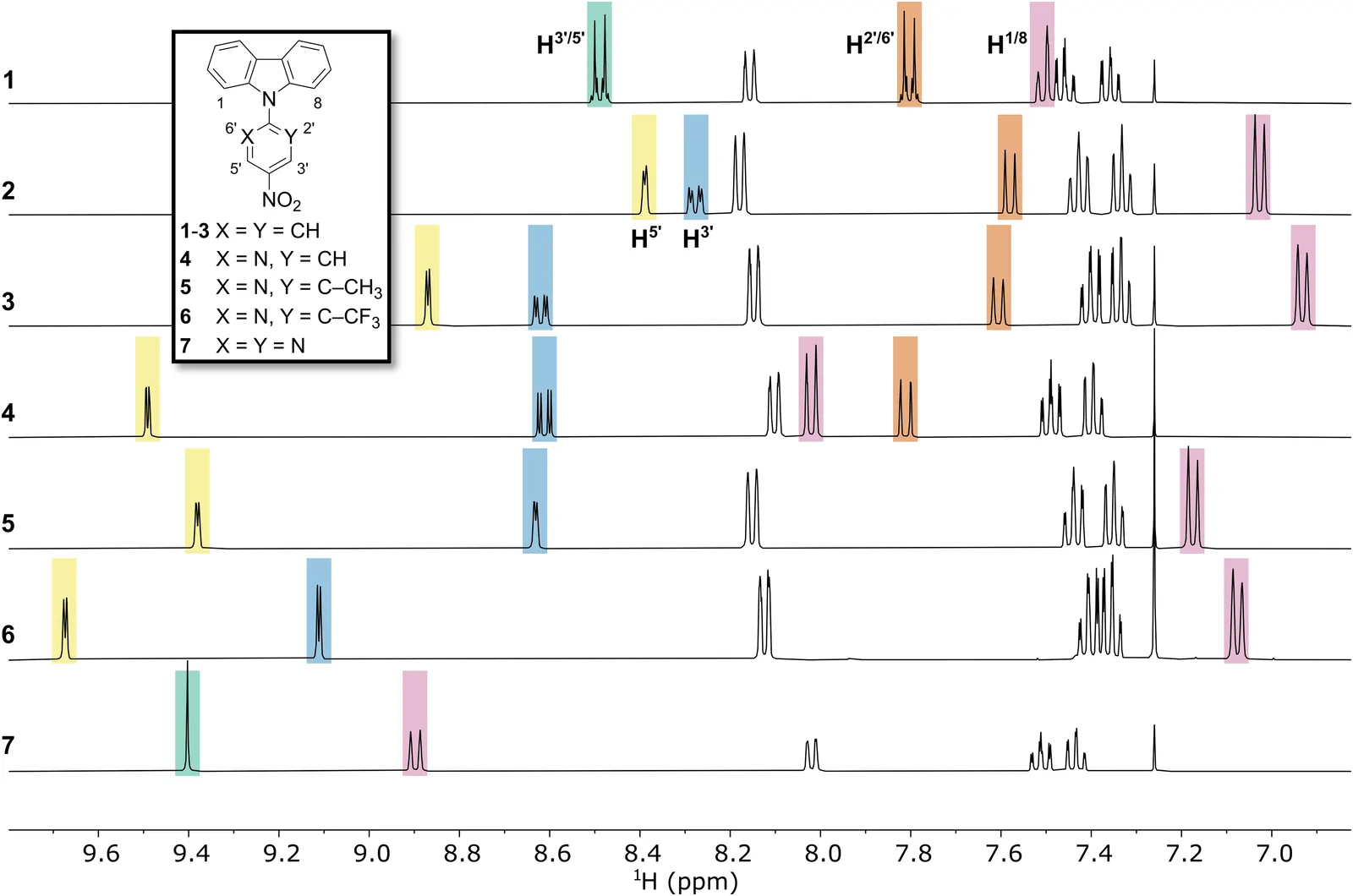
The aromatic region of the ^1^H-NMR spectrum for 1–7. The resonances discussed in the text are highlighted as H^1/8^ (purple), H^2′/6′^ (orange), and H^3′/5′^ (green) in 1 which decomposes into, H^3′^ (blue) and H^5′^ (yellow) for 2–6 as the molecular symmetry is broken. The dashed lines connecting the H^1/8^ resonances for 1, 4, and 7 highlights the large downfield shift due to intramolecular hydrogen bonding, which should be considered alongside the H^1/8^ resonances for 2 and 3 which both shift upfield and to a much lesser extent.

Next, we were interested in changes in the ^1^H chemical shifts observed between the proton NMR spectra of 1–7 (Fig. 5 and Table 2) to obtain insights into the local bonding environment of the H^1/8^ of carbazole in solution. We would expect protons involved in hydrogen bonding to have higher chemical shift values.

**Table 2:** Chemical shifts of the aromatic protons from the ^1^H-NMR spectra of 1–7 in CDCl_3_ (*δ* = 7.26 ppm) solution

	H^1/8^	H^2/7^	H^3/6^	H^4/5^	H^2′^	H^6′^	H^3′^	H^5′^
1	7.51	7.46	7.36	8.16	7.80	7.80	8.49	8.49
2	7.03	7.43	7.33	8.18	—	7.58	8.39	8.28
3	6.93	7.40	7.33	8.15	—	7.61	8.87	8.62
4	8.02	7.49	7.40	8.10	7.81	—	8.61	9.49
5	7.17	7.44	7.35	8.15	—	—	8.63	9.38
6	7.08	7.41	7.35	8.12	—	—	9.11	9.67
7	8.90	7.51	7.43	8.02	—	—	9.40	9.40

In 1, H^1/8^ display a resonance at δH^1/8^ = 7.51 ppm. This is approximately 0.17 ppm downfield from that observed for 9-phenylcarbazole,^15^ and is due to the influence of –NO_2_ as a strong EWG. In 2 and 3, the –CH_3_ and –CF_3_ groups have been shown in the previous sections to impose progressively larger ∠N_Cbz_–C_Ar_ twist angles and, in their NMR spectra, this results in upfield shifts of δH^1/8^ to 7.03 (2) and 6.93 ppm (3). The larger upfield shift for the –CF_3_-bearing compound is also apparent for 5 and 6. Assuming the absence of significant IHB in 5 and 6, these observations indicate that the magnitude of δH^1/8^ is dictated to a large extent by the size of ∠N_Cbz_–C_Ar_ rather than whether the group causing the twist is an EDG or an EWG.

The influence of IHB becomes apparent in 4 where δH^1/8^ has shifted >0.5 ppm downfield to δH^1/8^ = 8.02 ppm, and in the very high δH^1/8^ = 8.88 ppm in pyrimidine compound 7. This is a downfield shift of 1.82 ppm in comparison with 1, and 0.86 ppm in comparison with 4. Interestingly, when the δH^1/8^ of 7 is compared with that of an analogue which has no substituents on the pyrimidine ring, the absolute value is essentially unchanged.^16^ This suggests limited influence of the –NO_2_ group on the H-bonding ability of the pyrimidine N_Ar_ atoms, which can be rationalised by the fact that the N_Ar_ atoms and the –NO_2_ group of 7 are *meta*-conjugated with respect to each other. In contrast H^3′^ and H^5′^ display a 0.60 ppm downfield shift by the NO_2_, as may be expected.

Finally, some additional systematic differences can be observed upon changing the phenyl ring of 1–3 to a pyridine ring in 4–6. The H^2′^ atom of 4 sits *meta*-to the N atom of the pyridine and experiences a small upfield shift of 0.01 ppm in comparison with the H^2′/6′^ resonance of 1. In contrast, H^3′^ and H^5′^ are *para*- and *ortho*- to the pyridine N, respectively which causes a larger change in chemical shift than is observed for H^2′^, pushing the chemical shifts downfield. The magnitude of the changes in chemical shift of the H^3′^ and H^5′^ protons indicate much greater sensitivity towards the influence of the –CH_3_ and –CF_3_ groups in 4–6 compared with 1–3.

Overall, these results support the findings that in solution IHB is only significant for 4 and 7, and that the apparent IHB observed in the crystal structures of 5 and 6 are being supported by packing interactions.

### Cyclic voltammetry

**Fig. 6:**
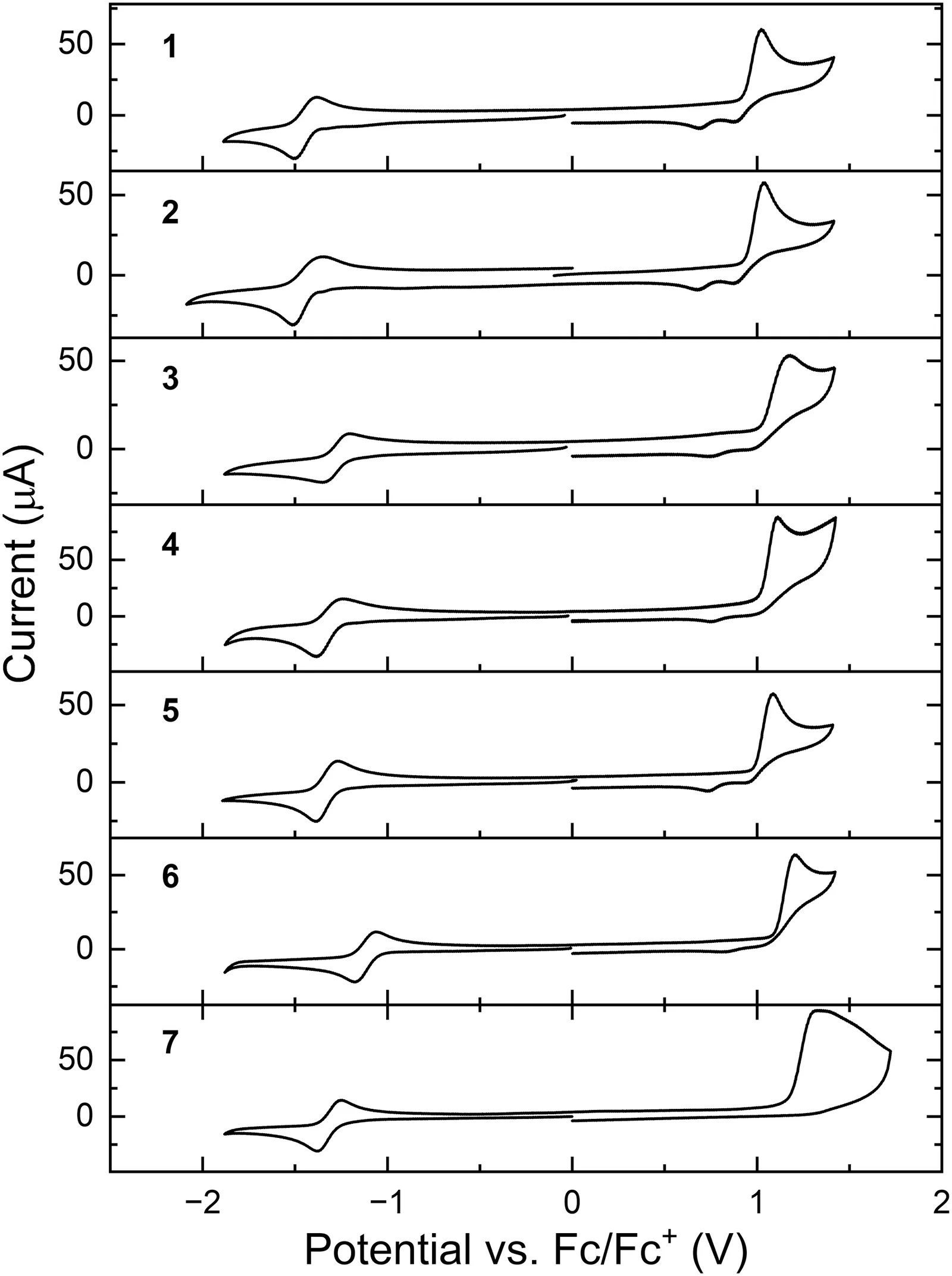
Cyclic voltammograms of 1–7 in 1 mM CH_2_Cl_2_ solution with 0.1 M [*n*-Bu_4_N][BF_4_] supporting electrolyte. Scan rate = 100 mV s^−1^.

Cyclic voltammetry was performed on 1–7 (Fig. 6 and Table 3). All of the molecules display a single irreversible oxidation, and a reversible, or quasi-reversible reduction. The oxidation occurs over the electron-rich carbazole and the reduction over the nitro(hetero)aryl. This is supported by the HOMO and LUMO distributions predicted from the *ab initio* calculations (Fig. S41). The irreversible nature of the oxidation is due to oxidative dimerisation of the carbazole radical cation, which is typical for carbazoles that do not have substituents on the 3- and/or 6-positions.^2*a*,22^

**Table 3:** Cyclic voltammetry results for 1–7

	*E* ^ox^ (V)	*E* ^rd^ (V)	HOMO^a^ (eV)	LUMO^b^ (eV)	*E* ^elec^_g_ c (eV)
1	+1.03^ir^	−1.50/−1.35^q^	−5.98	−3.80	2.18
2	+1.04^ir^	−1.51/−1.35^q^	−5.98	−3.80	2.18
3	+1.17^ir^	−1.33/−1.20^q^	−6.08	−3.94	2.14
4	+1.11^ir^	−1.39/−1.25^q^	−6.03	−3.89	2.14
5	+1.09^ir^	−1.39/−1.26^r^	−6.05	−3.90	2.15
6	+1.21^ir^	−1.20/−1.02^r^	−6.15	−4.07	2.08
7	+1.32^ir^	−1.38/−1.25^r^	−6.16	−3.86	2.30

a *E*_HOMO_ = −(*E*^ox^_onset_*vs.* Fc/Fc^+^ + vacuum level) eV.

b *E*_LUMO_ = −(*E*^red^_onset_*vs.* Fc/Fc^+^ + vacuum level) eV. *E*^ox^_onset_ and *E*^red^_onset_ are the onset of the first oxidation and reduction peak respectively. Vacuum level taken as −5.10 eV.^23^

c *E* ^elec^_g_ = −(HOMO–LUMO). ^ir^Irreversible. ^q^Quasireversible. ^r^Reversible.

The presence of the –CF_3_ group leads to a higher first reduction potential (*E*^rd^) for 3 (in comparison with 1 and 2) and 6 (in comparison with 4 and 5) so stabilising the HOMO by approximately 0.10 eV. Considering that both the experimental and calculated structures have the –CF_3_ group positioned over the face of the carbazole and imposing itself to the extent of distorting the local bonding around the N_Cbz_–C_Ar_ bond, and that there is an absence of any significant influence of the –CH_3_ group on the HOMO energies of 2 and 5 (when compared with 1 and 4, respectively) this stabilising of the HOMO likely arises primarily from through-space, rather than through-bond, inductive effects. The presence of –CH_3_ as an EDG in 2 and 5 has negligible influence on the LUMO energy, as evidenced by the identical reduction potentials compared with 1 and 4 respectively. In contrast, the –CF_3_ EWG stabilises the LUMO by 0.14 eV for 3 (compared with 1), and 0.08 eV for 6 (compared with 4). The reduction waves have relatively large separations between the cathodic and anodic peaks which may be explained by changes of configuration around N_Cbz_–C_Ar_ upon formation of the radical anion.

Comparing the electrochemical properties of the series 1, 4 and 7, between 1 and 4 the HOMO is stabilised by 0.10 eV, then between 4 and 7 the HOMO energy drops by a further 0.08 eV. In other words, the system is becoming progressively harder to oxidise as N atoms are introduced into the aryl substituent, despite the fact that the carbazole moiety as an electron donor remains unchanged. This suggests stronger electronic coupling between Cbz and the nitro-aryl acceptor as a consequence of the ∠N_Cbz_–C_Ar_ decreasing. For similar reasons, the LUMO of 7 is actually intermediate to that of 1 and 4, despite pyrimidine being a stronger acceptor than pyridine. Overall, this actually gives 7 the largest electrochemical HOMO–LUMO gap across all of the compounds 1–7 at 2.30 eV despite its planar structure. Visual analysis of the HOMO–LUMO distributions reveals that the contribution from N_Cbz_ to the HOMO diminishes, while its contribution to the LUMO increases. This is clearly observed when comparing 1 and 7 where the HOMO distribution of 7 is much more similar to a biphenyl group than a carbazole resulting in a deeper HOMO energy.

### UV-Vis and photoluminescence

**Fig. 7:**
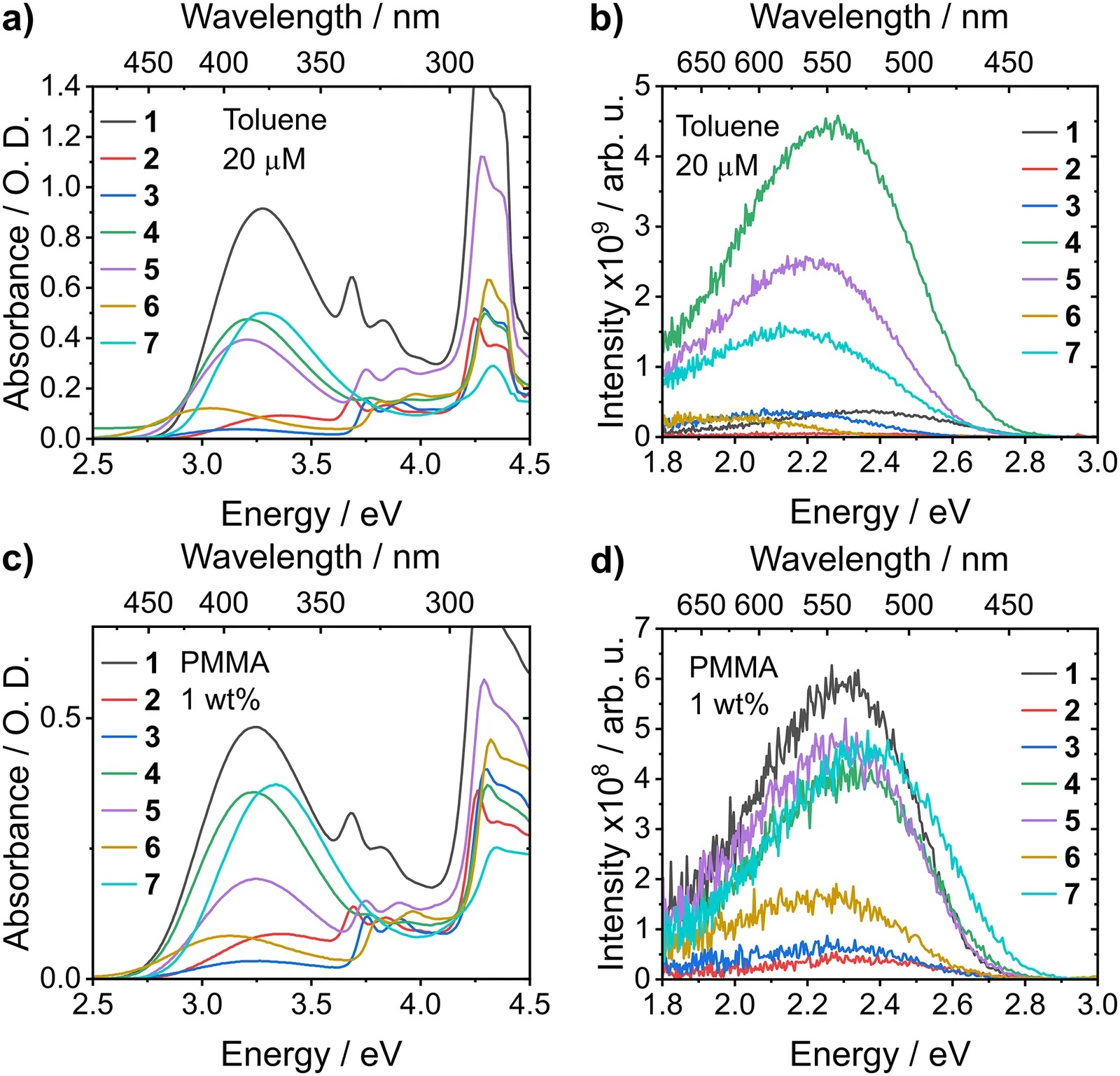
The (a) absorption and (b) photoluminescence spectra of compounds 1–7 in toluene solution at a concentration of 20 µM and the (c) absorption and (d) photoluminescence of those same compounds in doped thin films at 1 wt% in PMMA host. Intensity has been corrected to be a function of energy according to the Jacobian in ref. 24.

UV-Vis and photoluminescence spectra were obtained in a range of solvents (20 µM) and at 1 wt% in poly(methyl methacrylate) (PMMA) films. The spectra obtained in toluene solution and PMMA are shown in Fig. 7 and the results summarised in Table 4. Additional experimental details and spectra are in the SI (Table S3 and Fig. S42–S45). The photoluminescence quantum yield (PLQY) of the molecules in PMMA were less than 5% for all compounds and for some of the systems less than 1% (Table S4). The low PLQYs for these systems can be noted by the signal to noise ratio in the photoluminescence spectra (Fig. 7d).

**Table 4:** UV-Vis absorbance and photoluminescence results for 1–7 in 20 µM toluene solution and 1 wt% in PMMA thin film (in brackets)

	*λ*_abs_ (nm)	*λ*_em_ (nm)	Stokes shift (eV)	*E* ^opt^_g_ a (eV)
1	379 (382)	524 (538)	0.91 (0.94)	2.86 (2.76)
2	369 (369)	537 (539)	1.05 (1.06)	2.92 (2.85)
3	391 (382)	579 (547)	1.03 (0.98)	2.76 (2.74)
4	387 (384)	546 (533)	0.93 (0.90)	2.84 (2.77)
5	387 (383)	561 (538)	0.99 (0.93)	2.80 (2.76)
6	408 (396)	605 (553)	0.99 (0.89)	2.61 (2.64)
7	377 (371)	578 (525)	1.14 (0.98)	2.91 (2.88)

a *E* ^opt^_g_ = 1240.68/*λ*_onset_ where *λ*_onset_ is the onset of the lowest energy absorbance.

In all conditions employed the longest wavelength absorbance and the emission band have a Gaussian profile characteristic of ICT emission, with the exception of the spectra obtained in non-polar methylcyclohexane (MCH). Increased fine structure in the MCH spectra is attributed to diminished influence from the ICT state with increased locally excited character.

All compounds absorb in a very similar region both in solution and film with the band edge occurring at approximately 2.75 eV (450 nm) with their emission peaking in the general area of 2.25 eV (550 nm) making them green/yellow emitters.

## Conclusions

In conclusion, comparisons between the X-ray crystallography determined structures of 1–7 in the solid state and gas phase structures from computational approaches reveal that only 4 and 7 demonstrate intramolecular hydrogen bonding between the carbazole and the heteroaryl group which persists in both the solid state and gas phase. This interaction is notably strong for 7 where there are two such bonds and is clearly apparent experimentally in the chemical shifts of the carbazole 1- and 8- protons in the solution phase ^1^H NMR spectra. Coupled with variation in the calculated ground and excited states structures, these observations indicate that any apparent intramolecular H-bonding interactions in the crystal structures for 2, 3, 5 and 6 are primarily a consequence of crystal packing supporting the IHB, rather than IHB itself. This provides direction towards how IHB might be implemented in the design of new molecular materials, especially for those intended for use in the bulk phase such as charge-transport and AIE materials.

It is noteworthy, that despite having the strongest acceptor moiety, the planar molecule 7 has one of the widest HOMO–LUMO gaps in this series, as indicated by both UV-vis and cyclic voltammetry. This is primarily due to the influence of the N atom of the carbazole shifting from being sequestered in the HOMO into the LUMO manifold. While the use of –NO_2_ groups here has led to only low PLQY, the use of alternative strong acceptor groups in a similar fashion may present routes to exploiting carbazole as a weaker donor, with knock-on influences on the magnitude and relative positioning of critical photophysical properties such as the S_1_ and T_1_ energies.

## Author contributions

J. R. M., G. C. M., L. Y., C. R. A., S. M. and S. W. completed the synthetic chemistry and J. R. M. and S. W. performed the electrochemistry. I. A. W. supervised these researchers. P. J. K. completed the computational work with F. P. supervising. Photophysical studies completed by R. M. P. with M. K. E. supervising. M. R. J. E. and S. J. T. curated the X-ray crystallography data and solved the structures. The work was conceptualized by I. A. W. who produced the original draft. M. R. J. E., F. P., and M. K. E. assisted with analysis, review and editing.

## Conflicts of interest

There are no conflicts to declare.

## Supplementary Material

CP-028-D6CP02608G-s001

CP-028-D6CP02608G-s002

## Data Availability

Supplementary information (SI) is available. This includes synthetic methods, NMR and mass spectra, crystal packing figures and tabulated crystallography data, computationally optimised structures with frontier orbital distributions, and additional photophysical data and plots. See DOI: https://doi.org/10.1039/d6cp02608g. CCDC 2402749–2402754 (1 and 3–7) contain the supplementary crystallographic data for this paper.^25*a*–*f*^ The other data supporting this article have been included as part of the SI.
