# Supplementary material for: Intramolecular hydrogen bonding enforced by crystal packing and vice versa in nitrophenyl-, nitropyridyl-, and nitropyrimidyl-bearing carbazoles
Source: Phys Chem Chem Phys. 2026 Aug 25;28(36):22280–8. doi: 10.1039/d6cp02608g (PMC13614093; doi:10.1039/d6cp02608g)
Supplement: Supplementary file 1 [file CP-028-D6CP02608G-s001.pdf]

This journal is © the Owner Societies 2026

Supporting Information

Intramolecular Hydrogen Bonding Enforced by Crystal Packing and *vice versa* in Nitrophenyl-, Nitropyridyl- and Nitropyrimidyl Carbazoles

Jai-Ram Mistry,[a] Patrick J. Kimber,[a] Ruth M. Pollard,[b] Glen C. Masterson,[a] Lingyu Yang,[a] Chloe R. Antonen,[a] Stephanie Montanaro,[a] Stefan Warrington,[a] Simon J. Teat,[c] Mark R. J. Elsegood,[a] Felix Plasser,[a] Marc K. Etherington,[b] and Iain A. Wright[d*]

Addresses:

a Department of Chemistry, Loughborough University, Epinal Way, Loughborough, Leicestershire, LE11 3TU, United Kingdom.

b School of Engineering, Physics and Mathematics, Northumbria University, Ellison Place, Newcastle upon Tyne, NE1 8ST, United Kingdom.

c Advanced Light Source, Lawrence Berkeley National Laboratory, Cyclotron Road, Berkeley, CA, 94720, United States of America.

d EaStCHEM School of Chemistry, University of Edinburgh, Joseph Black Building, David Brewster Road, Edinburgh, EH9 3FJ, United Kingdom. Email: iain.wright@ed.ac.uk

Table of Contents

General Methods S3

Synthetic Methods S4

^{1}H and ^{13}C NMR Spectra S11

Mass Spectra S19

Crystallography S27

Computational S38

Photophysics S39

References S44

General Methods

All reactants and reagents were purchased from commercial suppliers and used without further purification unless otherwise stated. Column chromatography was carried out using silica gel 40-60 μm mesh (Fluorochem). Analytical thin-layer chromatography was performed on precoated aluminium silica gel 60 F254 plates (Merck), which were visualised using ultraviolet light (254/365 nm).

NMR spectra were recorded on Jeol ECS 400 MHz and Jeol ECZ 500 MHz spectrometers. Chemical shifts are reported in ppm downfield of tetramethylsilane (TMS) using TMS or the residual solvent as an internal reference. NMR spectra were processed using MestReNova. Multiplicities are reported as singlet (s), doublet (d), triplet (t), and multiplet (m).

Melting points were determined in open-ended capillaries using a Stuart Scientific SMP10 melting point apparatus at a ramping rate of 1 °C/min. They are recorded to the nearest 1 °C and are uncorrected.

Electrochemical studies were performed using an Autolab Potentiostat (PGSTAT302N). All electrochemical experiments were performed in a 4:1 acetonitrile:dichloromethane (DCM) mixture with 0.1 M $TBAPF_6$ as supporting electrolyte. Solutions were degassed with Ar prior to data collection. All electrochemistry experiments were carried out with a glassy carbon disk, a Pt wire coil counter electrode, and a $Ag/AgNO_3$ reference electrode (Ag wire in 0.01 M $AgNO_3$ solution). Unless stated otherwise, potentials are reported relative to the ferrocenium/ferrocene couple (Fc^+/Fc) as an internal standard with analyte molarity of *ca.* 10^{-4} M.

Synthetic methods

Compound 1[S1]

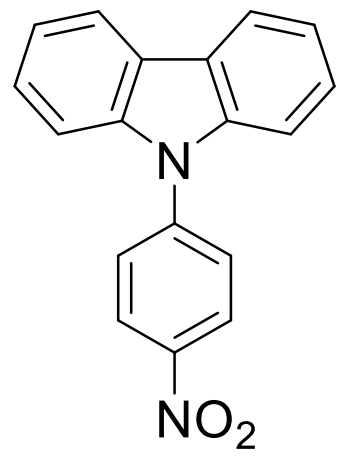

9*H*-Carbazole (2.51 g, 15 mmol) was dissolved in 40 mL of *N*,*N*-dimethylformamide (DMF) then solid K_2CO_3 (10.4 g, 75 mmol) was added with stirring. *p*-nitrofluorobenzene (8.47 g, 60 mmol) was subsequently added in one portion. The mixture was heated with stirring to reflux at 100 °C for 12 hours with stirring. After cooling, the mixture was poured into 100 mL of ice water. The solids formed were isolated by filtration then washed with H_2O and finally recrystallised from ethanol (EtOH) to yield the final product as a bright yellow crystalline solid (3.07g, 71%). M.p. 209-210 °C (lit. 208-210 °C).

^{1}H NMR (400 MHz, $CDCl_3$, ppm) δ 8.49 (2H, d, *J* = 9.0 Hz), 8.16 (2H, dt, *J* = 7.8, 1.0 Hz), 7.80 (2H, d, *J* = 9.0 Hz), 7.52–7.48 (2H, m), 7.44–7.48 (2H, m), 7.36 (2H, ddd, *J* = 8.1, 6.9, 1.2 Hz).
^{13}C NMR (101 MHz, $CDCl_3$, ppm) δ 146.0, 144.0, 140.0, 126.9, 126.6, 125.7, 124.3, 121.4, 120.8, 109.7.
HRMS (MALDI, m/z) 289.0971 [$M+H^+$], requires 289.0972 for [$C_{18}H_{13}N_2O_2^+$]
Elemental analysis: Calculated for $C_{18}H_{12}N_2O_2$: C, 74.99; H, 4.20; N, 9.72. Found C, 74.93; H 4.18; N; 9.67.

Compound 2

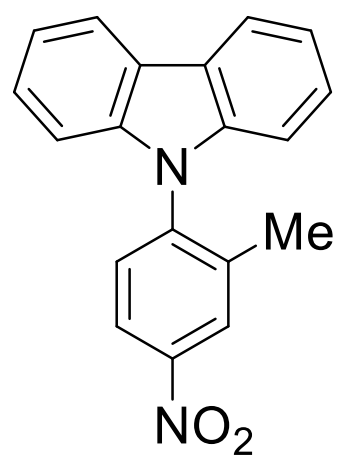

9*H*-Carbazole (2.51 g, 15 mmol) was dissolved in 40 mL of DMF then solid K_2CO_3 (10.4 g, 75 mmol) was added with stirring. 2-Fluoro-5-nitrotoluene (4.65 g, 30 mmol) was subsequently added in one portion. The mixture was heated with stirring to reflux at 100 °C for 12 hours with stirring. After cooling, the mixture was poured into 100 mL of ice water. The solids formed were

isolated by filtration then washed with H_2O and finally recrystallised from EtOH to yield the final product as a bright yellow crystalline solid (2.02 g, 45%). M.p. 137-138 °C.

^1H NMR (400 MHz, $CDCl_3$, ppm) δ 8.39 (1H, d, *J* = 2.6 Hz), 8.28 (1H, dd, *J* = 8.6, 2.6 Hz), 8.18 (2H, d, *J* = 7.5 Hz), 7.58 (1H, d, *J* = 8.6 Hz), 7.43 (2H, ddd, *J* = 8.3, 7.2, 1.3 Hz), 7.33 (2H, ddd, *J* = 8.1, 7.2, 1.0 Hz), 7.03 (2H, d, *J* = 8.2 Hz), 2.14 (3H, s).
^{13}C NMR (101 MHz, $CDCl_3$, ppm) δ 147.6, 142.4, 140.6, 139.3, 130.4, 126.9, 126.5, 123.7, 122.7, 120.8, 120.6, 109.7, 18.3.
HRMS (MALDI, m/z) 303.1127 [M+H$^+$], requires 303.1128 for [$C_{19}H_{15}N_2O_2^+$]
Elemental analysis: Calculated for $C_{19}H_{14}N_2O_2$: C, 75.48; H, 4.67; N, 9.27. Found C, 75.48; H 4.67; N; 9.30.

Compound 3[S2]

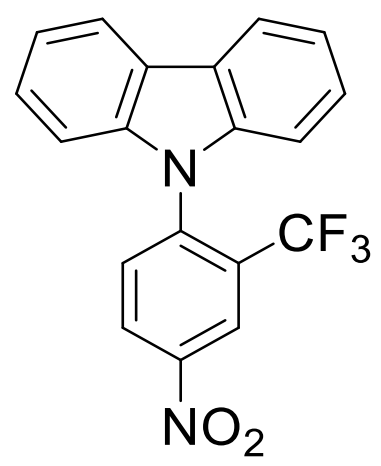

9*H*-Carbazole (2.51 g, 15 mmol) was dissolved in 20 mL of DMF then solid K_2CO_3 (10.4 g, 75 mmol) was added with stirring. 2-Fluoro-5-nitrobenzotrifluoride (6.27 g, 30 mmol) was subsequently added in one portion. The mixture was heated with stirring to reflux at 100 °C for 12 hours with stirring. After cooling, the mixture was poured into 100 mL of ice water. The solids formed were isolated by filtration then washed with H_2O and finally recrystallised from EtOH to yield the final product as a bright yellow crystalline solid (4.52 g, 85%). M.p. 138-139 °C (lit. 74-76 °C)

^1H NMR (400 MHz, $CDCl_3$, ppm) δ 8.87 (1H, d, *J* = 2.6 Hz), 8.62 (1H, ddd, *J* = 8.7, 2.6, 0.5 Hz), 8.15 (2H, ddd, *J* = 7.7, 1.4, 0.7 Hz), 7.61 (1H, d, *J* = 8.6 Hz), 7.40 (2H, ddd, *J* = 8.1, 7.2, 1.4 Hz), 7.31–7.35 (2H, m), 6.93 (2H, d, *J* = 8.1 Hz)
^{13}C NMR (101 MHz, $CDCl_3$, ppm) δ 147.8, 142.6, 142.5, 132.7 (q, $^2J_{CF}$ = 33.0 Hz), 128.6, 126.5, 124.1, 123.8 (q, $^3J_{CF}$ = 5.2 Hz), 123.3, 121.0, 120.6, 109.9
HRMS (MALDI, m/z) 357.0843 [M+H$^+$], requires 357.0845 for [$C_{19}H_{16}N_2O_2^+$]
Elemental analysis: Calculated for $C_{19}H_{11}F_3N_2O_2$: C, 64.05; H, 3.11; N, 7.86. Found C, 63,89; H 3.06; N; 7.85.

Compound 4[S3]

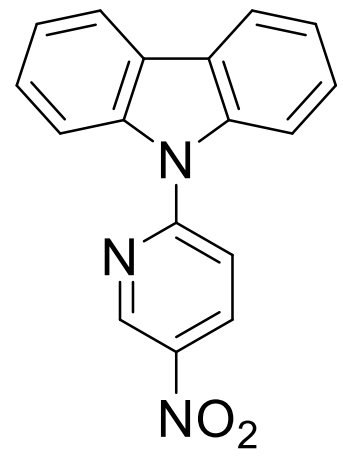

9*H*-Carbazole (0.80 g, 4.8 mmol) was added to a suspension of NaH (60% dispersion in paraffin,168 mg, 4.2 mmol) in 10 mL of anhydrous DMF at 0 °C. 2-Chloro-5-nitropyridine (0.73, 4.6 mmol) was added and the mixture left to stir for 1 hour at 0 °C then allowed to warm to room temperature and poured into 20 mL of ice water. The solids formed were isolated by filtration then washed with H_2O and finally recrystallised from iso-propanol (*i*PrOH) to yield the final product as a bright yellow crystalline solid (0.86 g, 62%). M.p. 185-187 °C. (lit. 185-188 °C).

^{1}H NMR (400 MHz, $CDCl_3$, ppm) δ 9.49 (1H, dd, *J* = 2.8, 0.6 Hz), 8.61 (1H, dd, *J* = 9.0, 2.8 Hz), 8.10 (2H, ddd, *J* = 7.7, 1.4, 0.7 Hz), 8.02 (2H, dt, *J* = 8.3, 0.8 Hz), 7.81 (1H, dd, *J* = 9.0, 0.7 Hz), 7.49 (2H, ddd, *J* = 8.4, 7.2, 1.3 Hz), 7.40 (2H, ddd, *J* = 8.2, 7.3, 1.0 Hz)
^{13}C NMR (101 MHz, $CDCl_3$, ppm) δ 156.2, 145.8, 140.8, 138.9, 133.7, 127.0, 125.6, 122.8, 120.5, 116.9, 112.3
HRMS (MALDI, m/z) 290.0923 [M+H$^+$], requires 290.0924 for [$C_{17}H_{12}N_3O_2^+$]
Elemental analysis: Calculated for $C_{17}H_{11}N_3O_2$: C, 70.58; H, 3.83; N, 14.53. Found C, 70.49; H 3.71; N; 14.28.

Compound 5

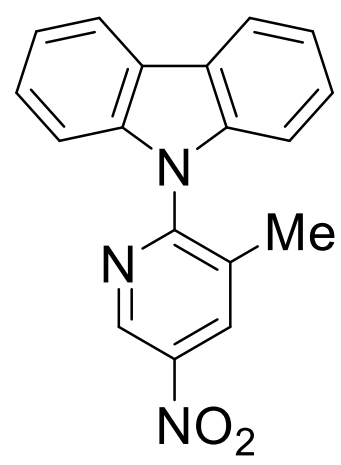

A solution of 9*H*-carbazole (0.40 g, 2.4 mmol), 2-chloro-3-methy-5-nitropyridine (0.35 g, 2 mmol), 4,5-bis(diphenylphosphino)-9,9-dimethylxanthene (Xantphos, 69 mg, 0.12 mmol), and Cs_2CO_3 (1.30 g, 4 mmol) in anhydrous tetrahydrofuran (THF, 7 mL) was degassed with Ar for 40 minutes after which $Pd_2(dba)_2$ (55 mg, 0.06 mmol) was added in one portion. The resulting dark purple solution was stirred at reflux for 12 hrs. After cooling to room temperature, the THF was removed under reduced pressure and the residue was dissolved in dichloromethane (DCM) and transferred to a separating funnel containing H_2O (20 mL). The organic layer was removed and

the aqueous phase extracted with additional DCM (3 × 20 mL). The combined organic layers were dried over $MgSO_4$ and the solvent subsequently removed under reduced pressure. Purification was achieved using flash chromatography (SiO_2; 9:1 *n*-hexane:ethyl acetate (EtOAc), R_f = 0.54) followed by recrystallisation from EtOH to yield the desired product as a yellow crystalline solid (0.105 g, 17 %). M.p. 195-196 °C.

^{1}H NMR (400 MHz, $CDCl_3$, ppm) δ 9.38 (1H, dd, *J* = 2.7, 0.7 Hz), 8.63 (1H, dd, *J* = 2.7, 0.8 Hz), 8.15 (2H, ddd, *J* = 7.7, 1.3, 0.7 Hz), 7.44 (2H, ddd, *J* = 8.3, 7.2, 1.3 Hz), 7.37–7.33 (2H, m), 7.17 (2H, dt, *J* = 8.2, 0.9 Hz), 2.29 (3H, s).
^{13}C NMR (101 MHz, $CDCl_3$, ppm) δ 154.7, 143.2, 139.7, 135.9, 131.8, 126.5, 124.4, 121.4, 120.8, 110.7, 18.4.
HRMS (MALDI, m/z) 304.1079 [$M+H^+$], requires 304.1081 for [$C_{18}H_{14}N_3O_2^+$]
Elemental analysis: Calculated for $C_{18}H_{13}N_3O_2$: C, 71.27; H, 4.32, N, 13.85. Found C, 71.07; H, 4.30; N, 13.60.

Compound 6

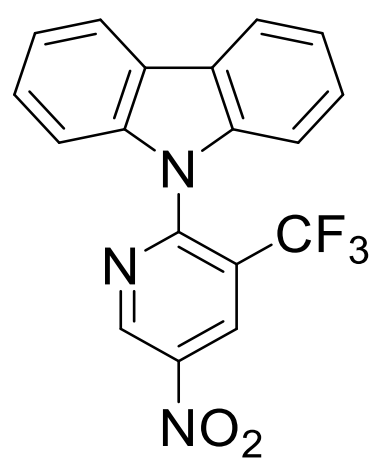

A solution of 9*H*-carbazole (0.20 g, 1.2 mmol), 2-chloro-3-trifluoromethyl-5-nitropyridine (0.23 g, 1.0 mmol), Xantphos (35 mg, 0.06 mmol.), and Cs_2CO_3 (0.65 g, 2.0 mmol) in anhydrous THF (5 mL) was degassed for 40 minutes with Ar. $Pd_2(dba)_2$ (27 mg, 0.03 mmol) was then added in one portion. The resulting dark purple solution was stirred at reflux for 12 hrs. After cooling to room temperature, the THF was removed under reduced pressure and the residue was dissolved in dichloromethane (DCM), then transferred to a separating funnel containing H_2O (20 mL). The organic layer was removed and the aqueous phase extracted with additional DCM (3 × 20 mL). The combined organic layers were dried over $MgSO_4$ and the solvent subsequently removed under reduced pressure. Purification was achieved using flash chromatography (SiO_2; 4:1 *n*-hexane:EtOAc, R_f = 0.49) followed by recrystallisation from EtOH to yield the desired product as fine yellow needles (49 mg, 14 %). M.p. 152-153 °C.

^{1}H NMR (400 MHz, $CDCl_3$, ppm) δ 9.67 (1H, d, J = 2.6 Hz), 9.11 (1H, d, J = 2.7 Hz), 8.12 (2H, ddd, J = 7.5, 1.4, 0.7 Hz), 7.42–7.38 (2H, m), 7.35 (2H, td, J = 7.4, 1.2 Hz), 7.08 (2H, d, J = 8.0 Hz)

^{13}C NMR (125 MHz, $CDCl_3$, ppm) δ 153.6, 148.7, 143.1, 141.6, 133.8 (q, $^{3}J_{CF}$ = 4.4 Hz), 126.2 (q, $^{2}J_{CF}$ = 34 Hz), 124.8, 121.9, 121.6 (q, $^{1}J_{CF}$ = 275 Hz), 120.7, 110.4.

HRMS (MALDI, m/z) 358.0796 [M+H$^+$], requires 358.0798 for [$C_{18}H_{11}F_3N_3O_2^+$]

Elemental analysis: Calculated for $C_{18}H_{10}F_3N_3O_2$: C, 60.51; H, 2.82; N, 11.76. Found C, 60.34; H, 2.68; N, 11.54.

Compound 7

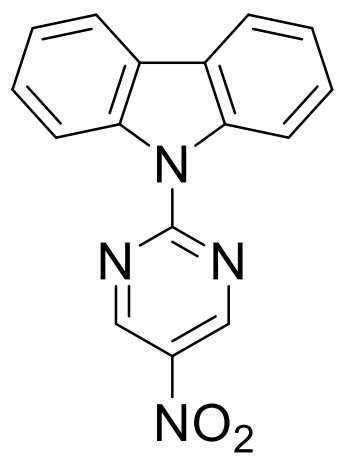

Approach 1:

Nucleophilic aromatic substitution between 9*H*-carbazole and 2-chloro-5-nitropyrimidine

9*H*-Carbazole (100 mg, 0.60 mmol) was dissolved in 4 mL of anhydrous DMF prior to addition of KOtBu (81 mg, 0.72 mmol) then stirred for 45 minutes. 2-Chloro-5-nitropyrimidine (115 mg, 0.72 mmol) was added whereupon the solution colour changed from yellow to orange. The mixture was heated to 70 °C for 24 hours then allowed to cool to room temperature and poured into 20 mL of ice water. Aqueous 1M HCl (1.2 mL) was added before the mixture was transferred to a separating funnel and the aqueous phase was washed with DCM (4 x 20 mL). The combine organic extracts were dried over MgSO4 prior to removal of the solvent under reduced pressure. Purification was achieved by column chromatography (SiO_2; 9:1 *n*-hexane:EtOAc, Rf = 0.59) to yield the desired product as yellow needles (42 mg, 24 %).

Approach 2:

Buchwald-Hartwig cross-coupling between 9*H*-carbazole and 2-chloro-5-nitropyrimidine

A solution of 9*H*-carbazole (150 mg, 0.9 mmol), 2-chloro-5-nitropyrimidine (431 mg, 2.7 mmol), Xantphos (40 mg, 0.07 mmol), and Cs_2CO_3 (652 mg, 2.0 mmol) in anhydrous THF (5 mL) was degassed for 40 minutes with Ar. $Pd_2(dba)_2$ (27 mg, 0.03 mmol) was then added in one portion and the mixture was heated to reflux overnight. Water (10 mL) was added before the mixture was transferred to a separating funnel and the aqueous phase was extracted with DCM (3 x 20 mL) and dried over MgSO4 prior to removal of the solvent under reduced pressure Purification was

achieved by column chromatography (SiO_2; 9:1 *n*-hexane:EtOAc, Rf = 0.59) to yield the desired product as yellow needles (32 mg, 18 %).

Approach 3:

Oxidative cyclisation of *N*-([1,1'-biphenyl]-2-yl)-5-nitropyrimidin-2-amine (Compound 8)

Step 1) Synthesis of Compound 8

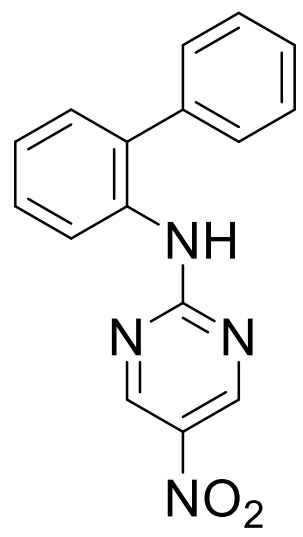

A solution of 2-aminobiphenyl (1.12, 6.6 mmol), 5-nitro-2-chloropyrimidine (1.03 g, 6.5 mmol) and potassium carbonate (2.69 g, 19.6 mmol) in DCM (50 mL) was heated to reflux for 72 hours in air. The initial purple solution turns yellow. After consumption of the staring material, water (50 mL) was added, the two phases were separated. The organic was washed with water (3 × 20ml). The organics where dried over $MgSO_4$ and evaporated to dryness to yield a crude yellow oil. This crude residue was purified using flash column chromatography (SiO_2; 9:1 *n*-hexane:EtOAc, Rf = 0.41) to yield a dark yellow solid (1.56 g, 82 %).

^{1}H NMR (400 MHz, $CDCl_3$, ppm) δ 9.11 (2H, s), 8.23 (1H, dd, *J* = 8.23, 1.2 Hz), 7.76 (1H, s), 7.51–7.34 (7H, m), 7.29 (1H, td, *J* = 7.4, 1.2 Hz).

^{13}C NMR (101 MHz, $CDCl_3$) δ 161.4, 155.2, 138.0, 135.8, 134.34, 134.30 134.2, 130.8, 129.4, 129.3, 128.5, 128.3, 125.72, 125.67, 122.6, 122.5.f

HRMS (MALDI, m/z) 293.1032 [M+H$^+$], requires 293.1033 for [$C_{16}H_{13}N_4O_2^+$]

Step 2) Oxidative Cyclisation of Compound 8

N-([1,1'-biphenyl]-2-yl)-5-nitropyrimidin-2-amine (0.8 g, 2.75 mmol), $Pd(OAc)_2$ (0.06 g, 10 mol%), $Cu(OAc)_2$ (1.65 g, 9.01 mmol) was heated to reflux in toluene (50 mL) under N_2 for 3 days. After this period, the reaction mixture was cooled to room temperature and quenched with the addition of water (50 mL). The two phases were separated, the organic layer was washed with a saturated NH_4Cl solution then washed twice with water (50 mL). The combined organics were dried over $MgSO_4$ and evaporated to dryness to yield a crude yellow residue Purification was

achieved by column chromatography (SiO_2; 9:1 *n*-hexane:EtOAc, Rf = 0.59) to yield the desired product as yellow needles to yield a dark yellow solid (0.18 g, 23 %). M.p. 233 °C

^{1}H NMR (400 MHz, $CDCl_3$, ppm) δ 9.40 (2H, s), 8.90 (2H, d, J = 8.4 Hz), 8.02 (2H, dd, J = 7.6, 0.8 Hz), 7.51 (2H, ddd, J = 8.5, 7.3, 1.5 Hz), 7.43 (2H, td, *J* = 7.4, 1.1 Hz).

^{13}C NMR (101 MHz, $CDCl_3$) δ 160.5, 154.0, 139.0, 137.3, 127.5, 127.3, 124.5, 119.8, 118.3.

HRMS (MALDI, m/z) 291.0875 [$M+H^+$], requires 291.0877 for [$C_{16}H_{11}N_4O_2^+$]

Elemental analysis: Calculated for $C_{16}H_{10}N_2O_2$: C, 66.20; H, 3.47; N, 19.30. Found C, 66.06; H 3.19; N; 19.23.

^{1}H and ^{13}C NMR Spectra

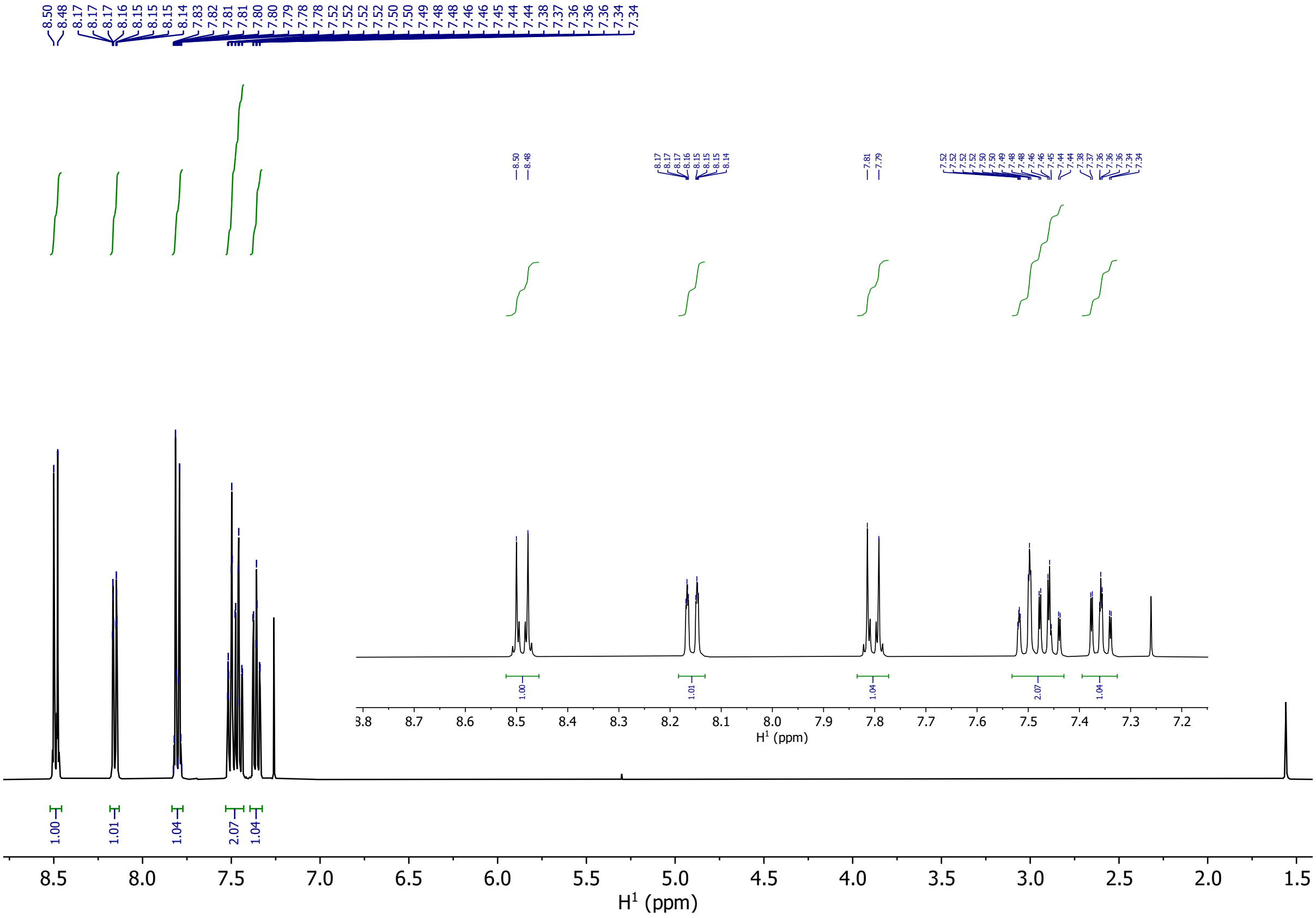

Figure S1: Compound **1** ^{1}H NMR spectrum.

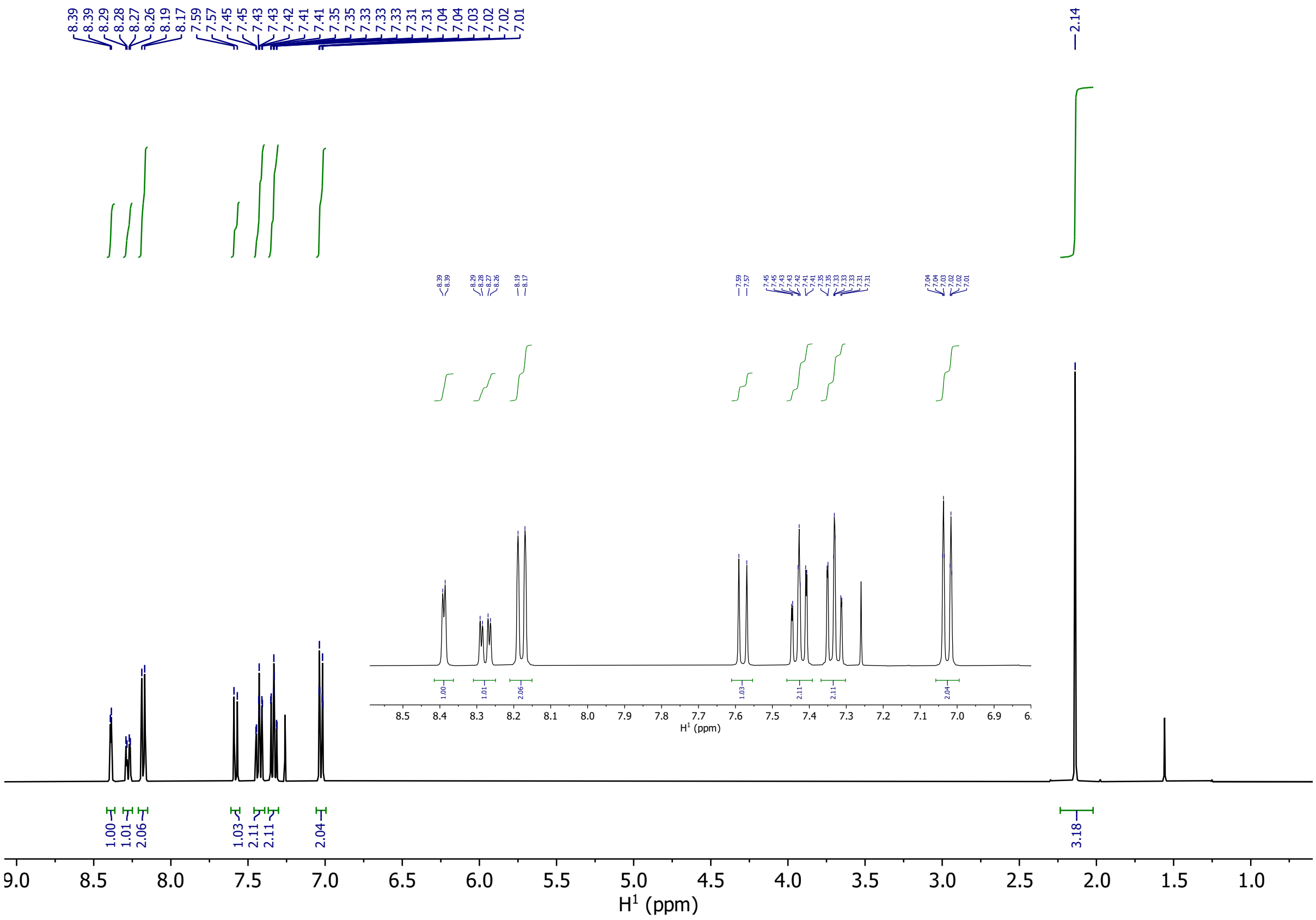

Figure S2: Compound **2** ^{1}H NMR spectrum.

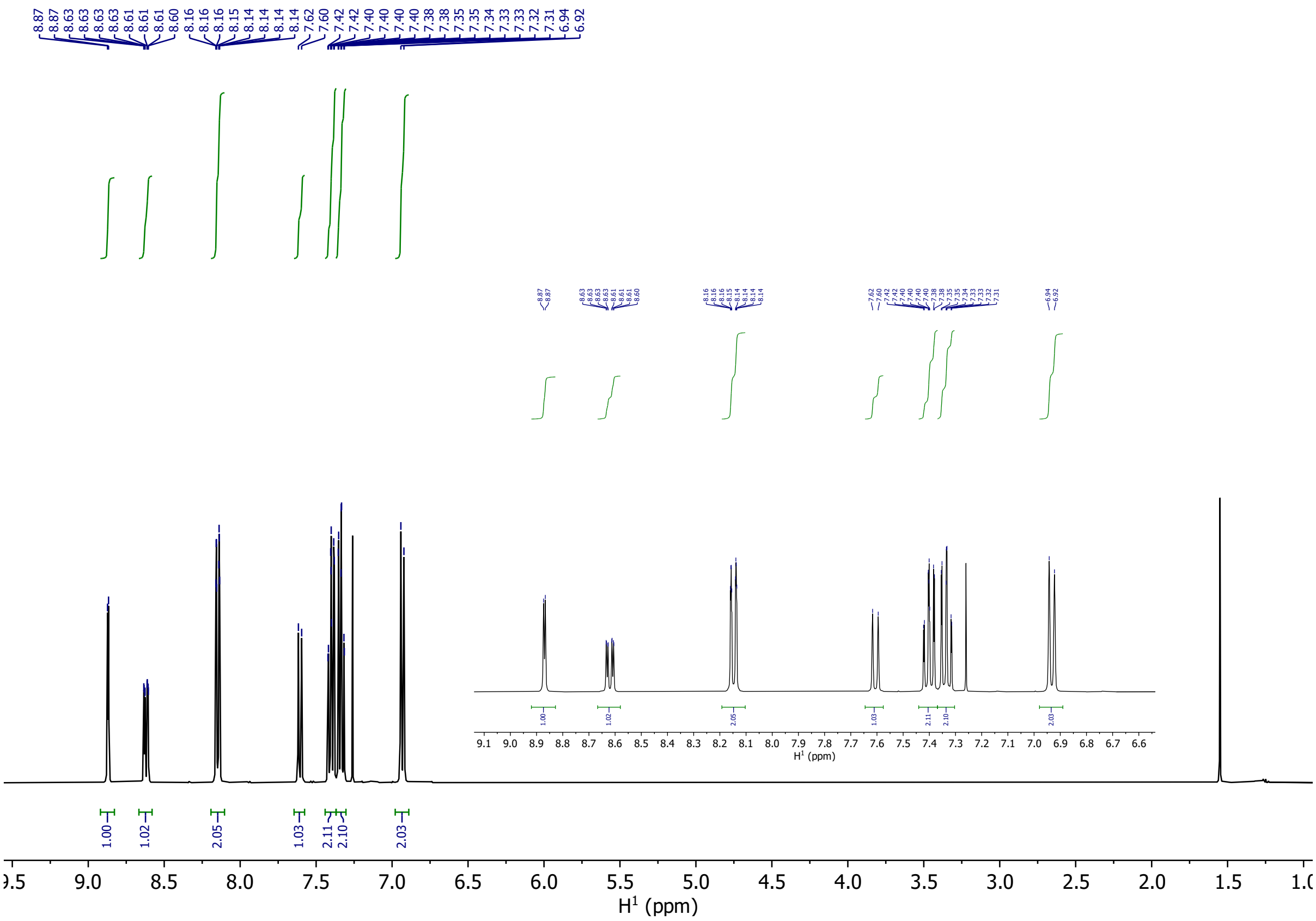

Figure S3: Compound **3** ^{1}H NMR spectrum.

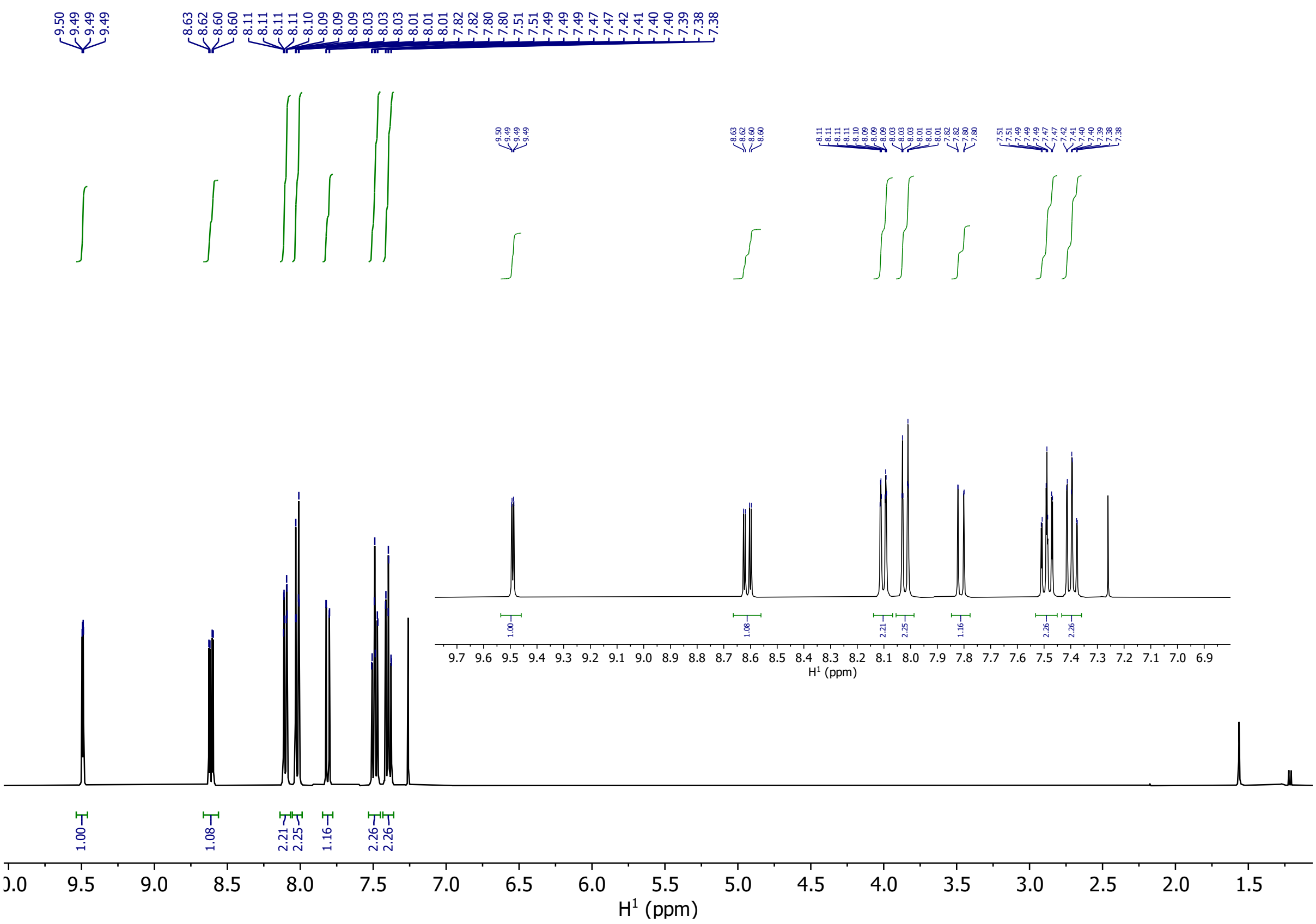

Figure S4: Compound **4** ^{1}H NMR spectrum.

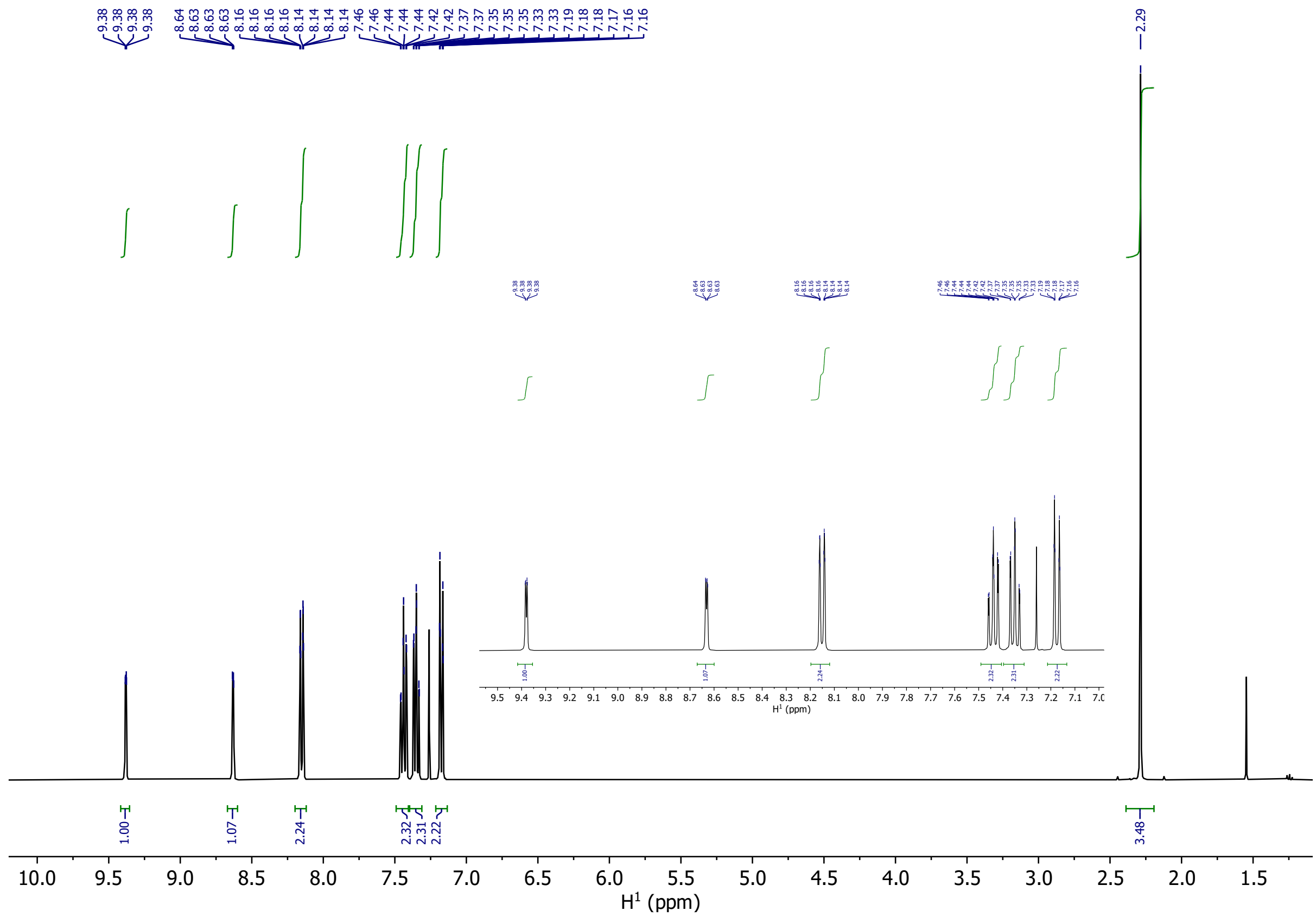

Figure S5: Compound **5** ^{1}H NMR spectrum.

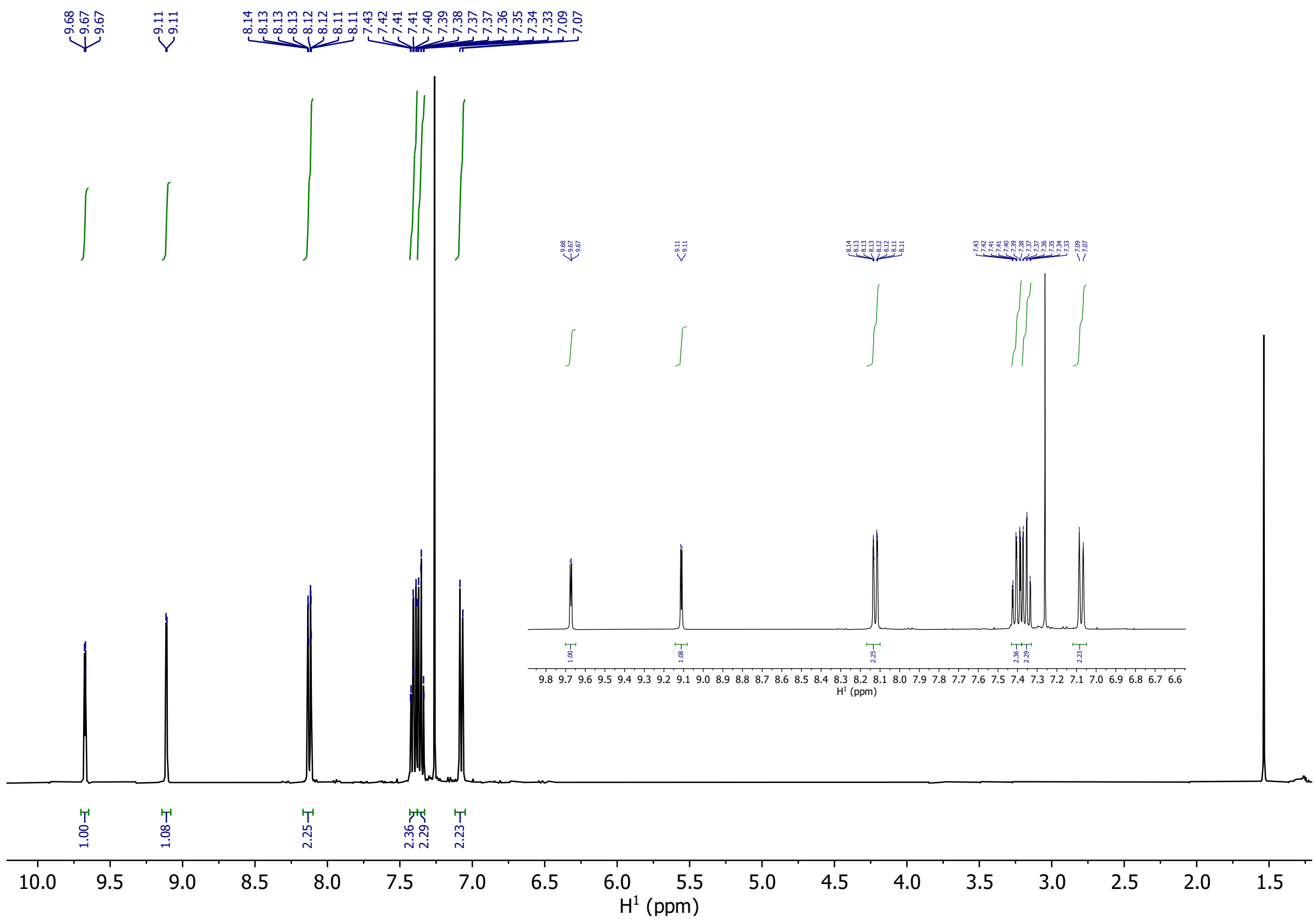

Figure S6: Compound **6** ^{1}H NMR spectrum.

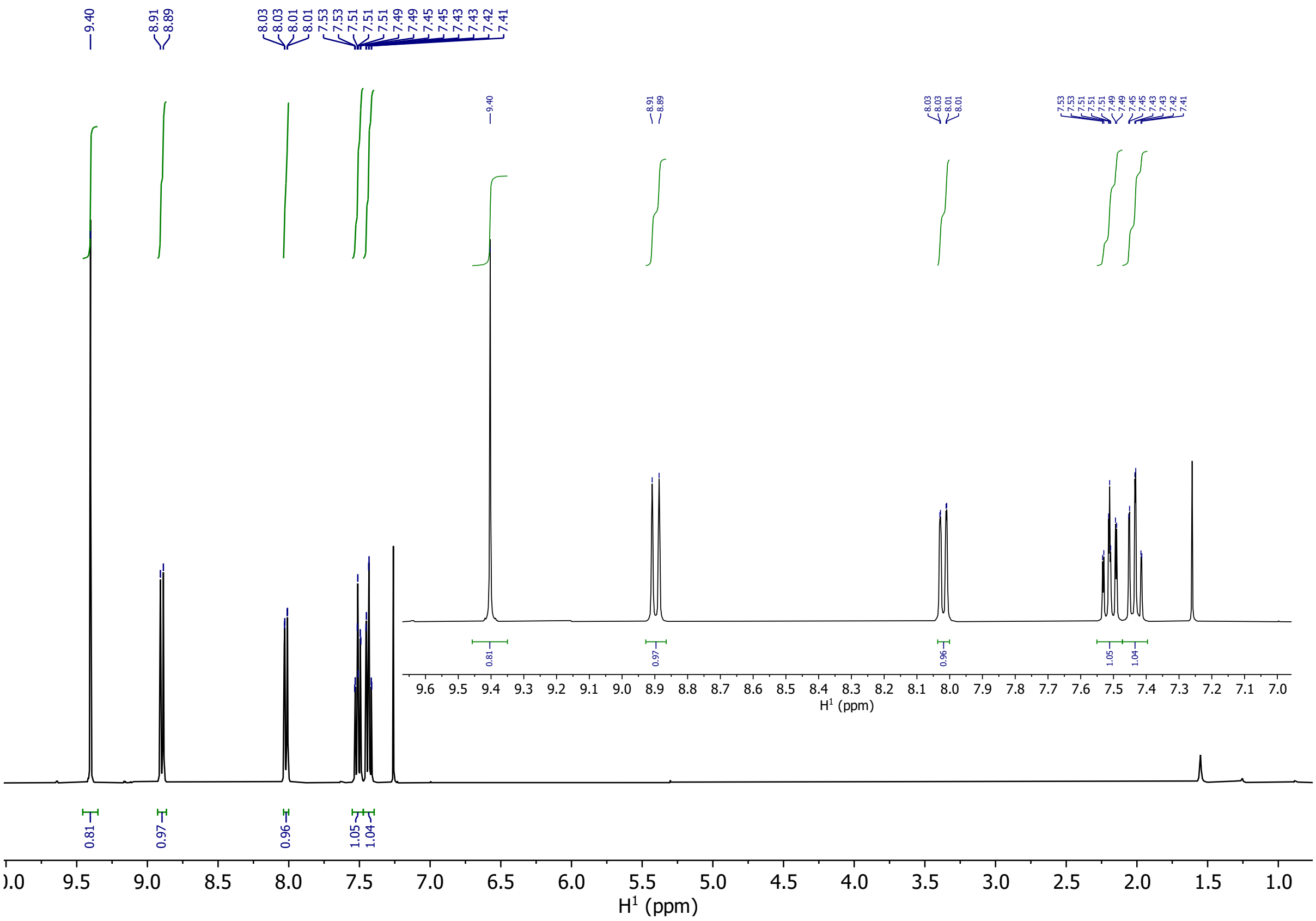

Figure S7: Compound **7** ^{1}H NMR spectrum.

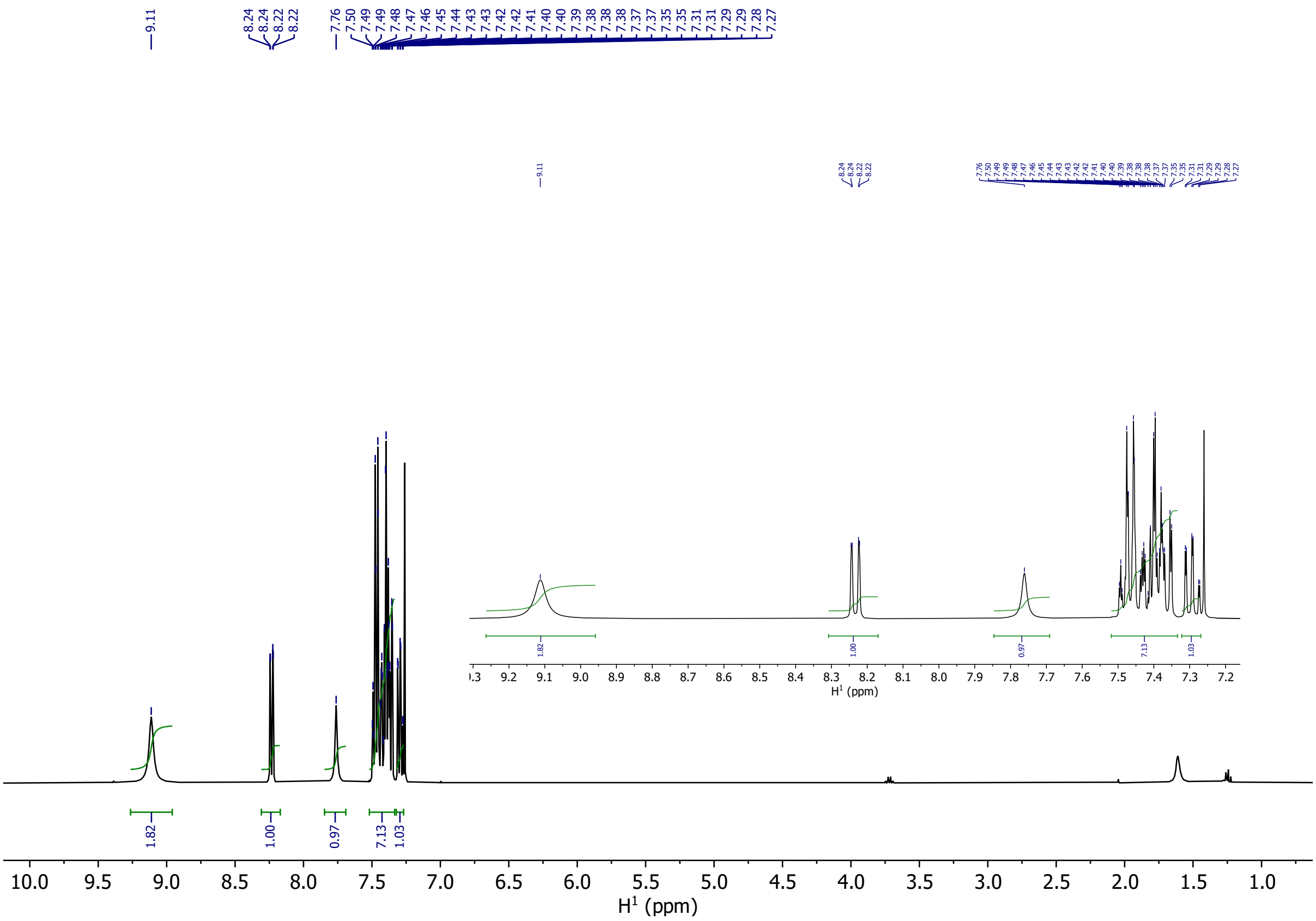

Figure S8: Compound **8** ^{1}H NMR spectrum.

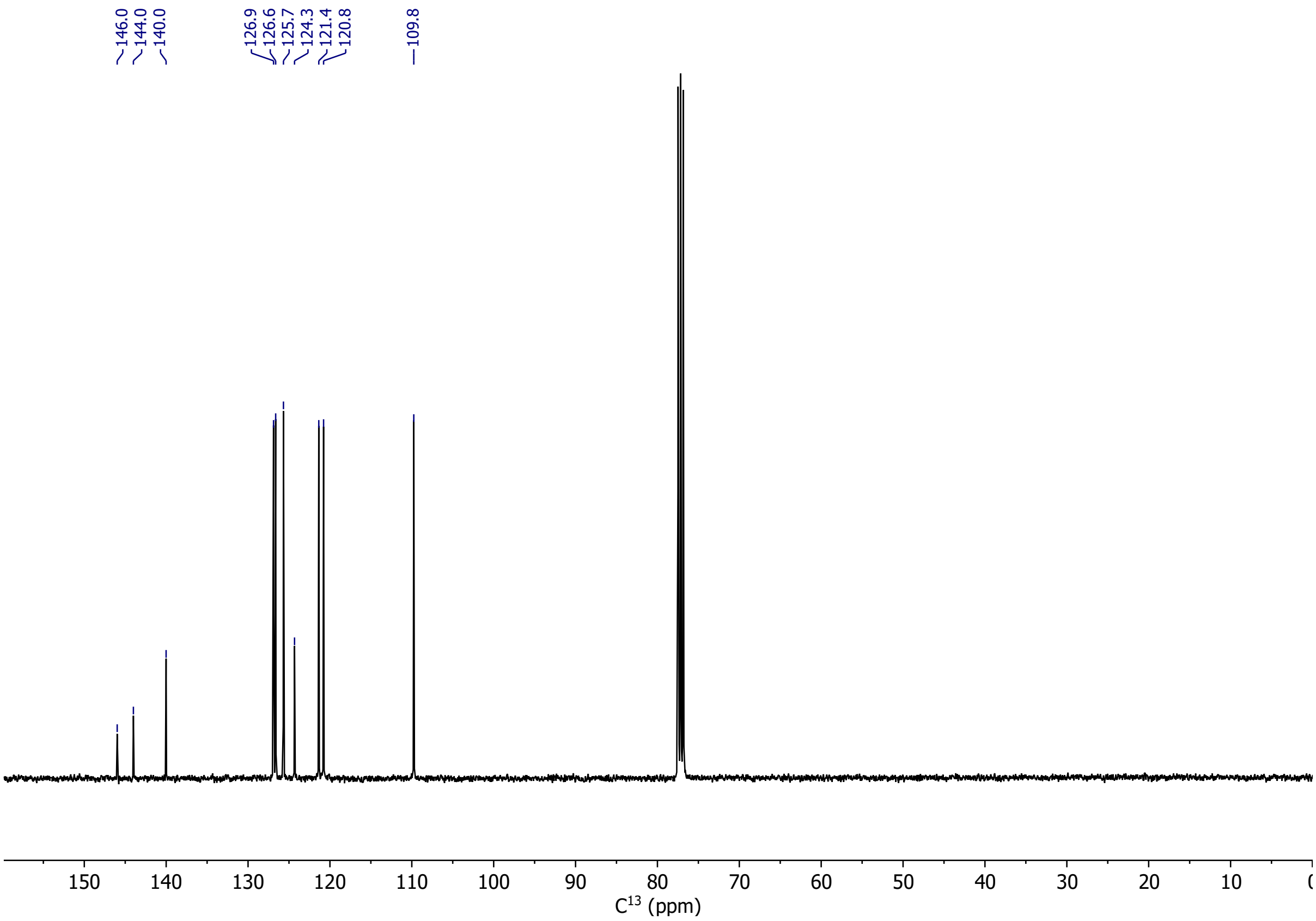

Figure S9: Compound **1** ^{13}C NMR spectrum.

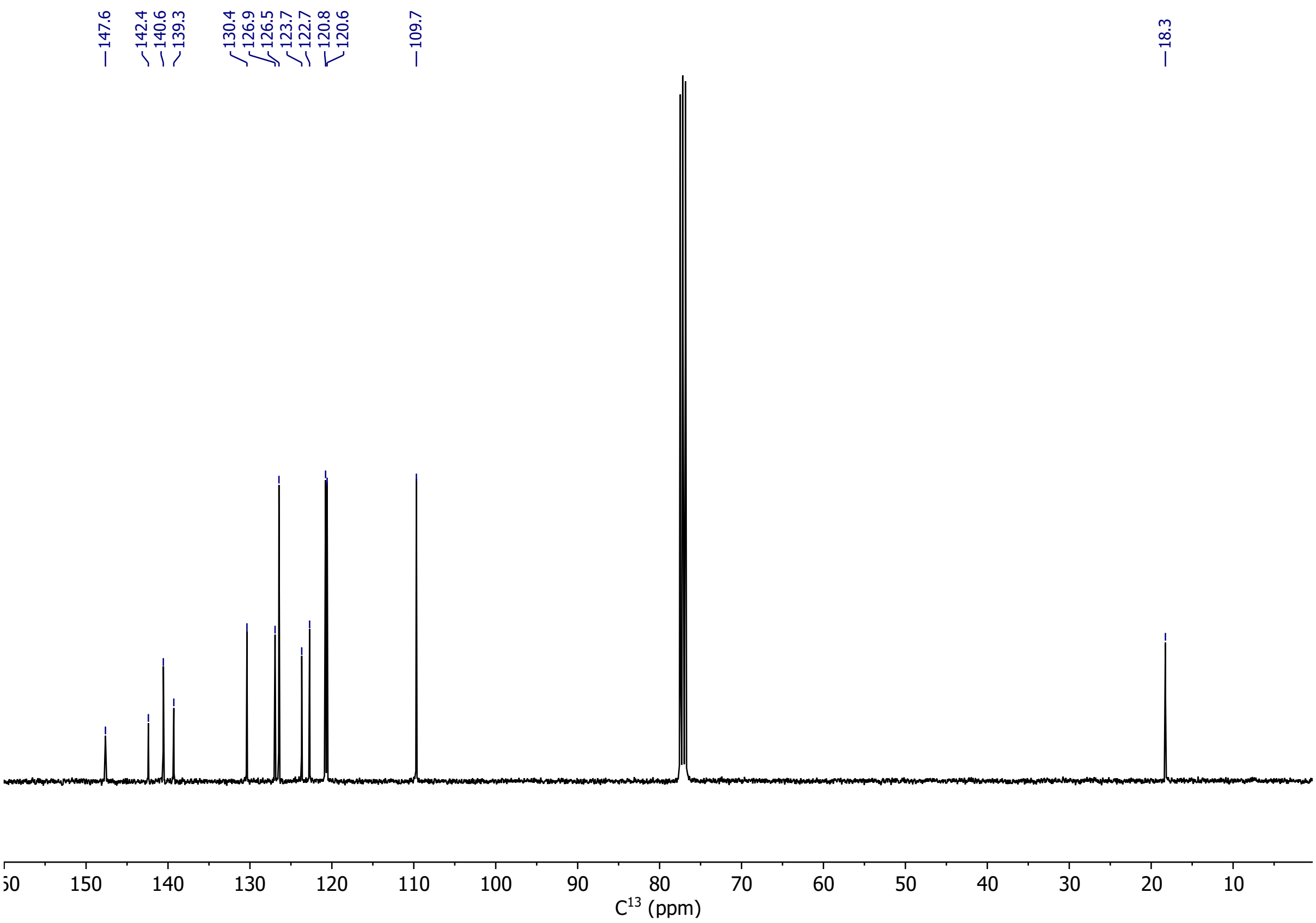

Figure S10: Compound **2** ^{13}C NMR spectrum.

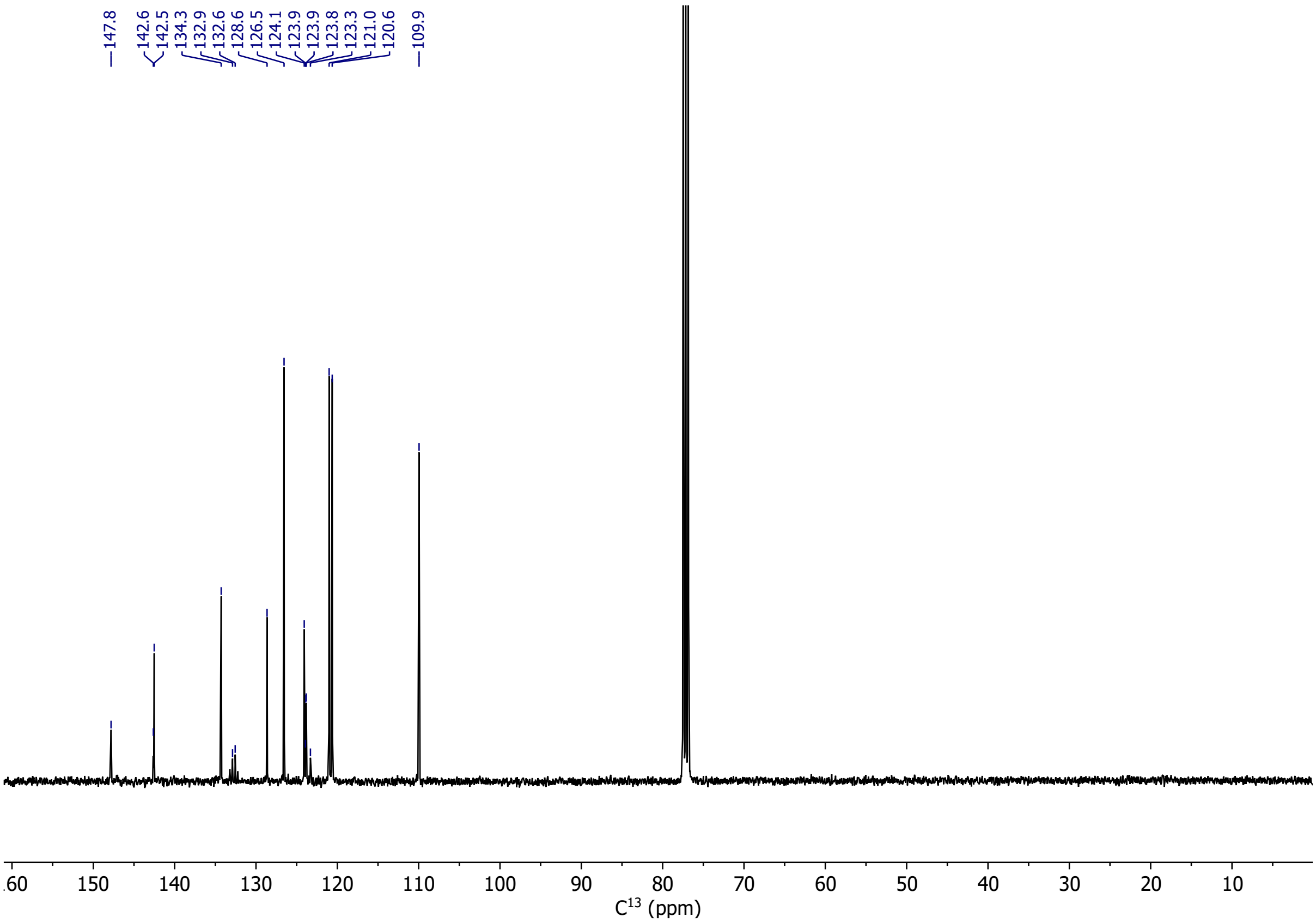

Figure S11: Compound **3** ^{13}C NMR spectrum.

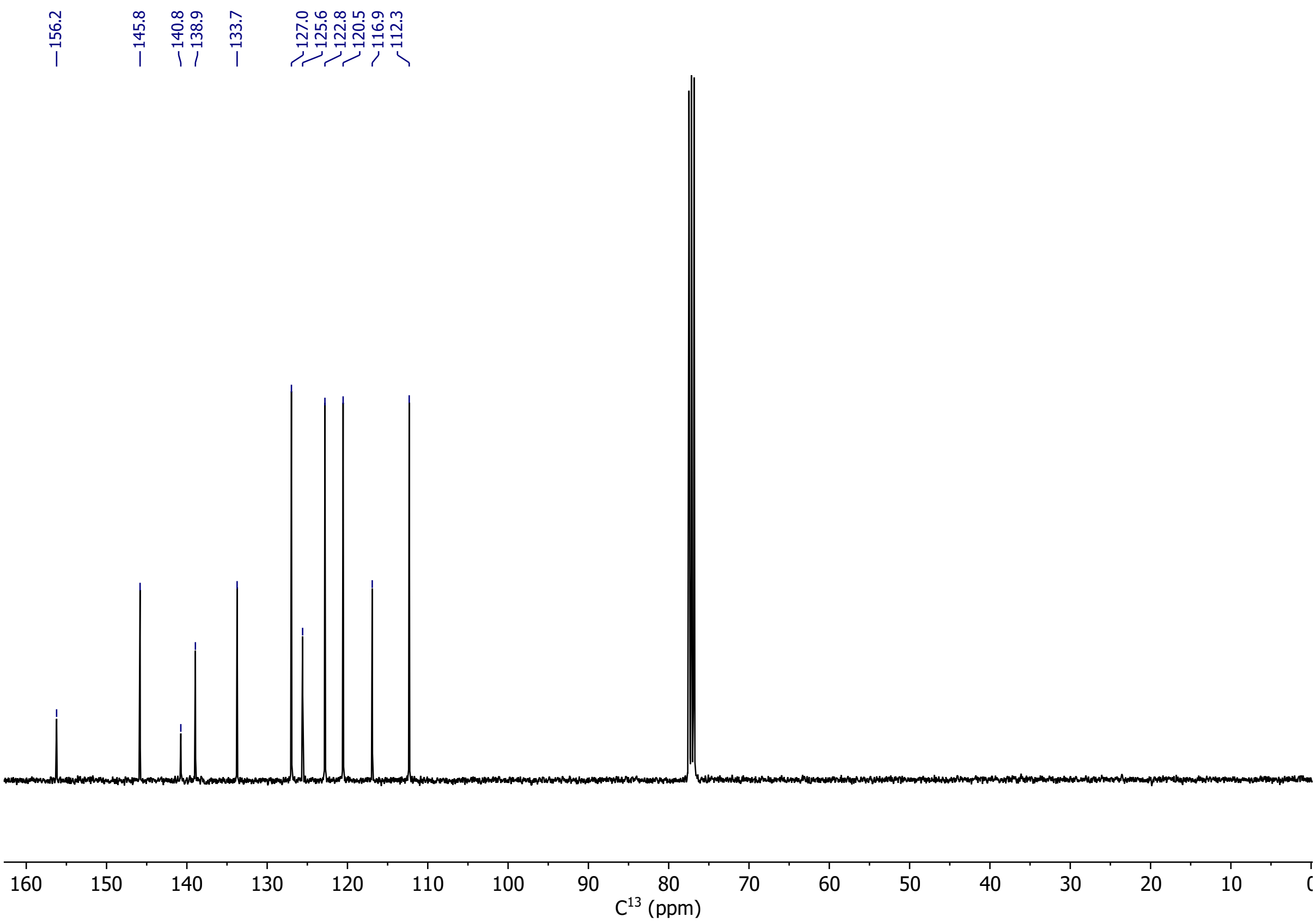

Figure S12: Compound **4** ^{13}C NMR spectrum.

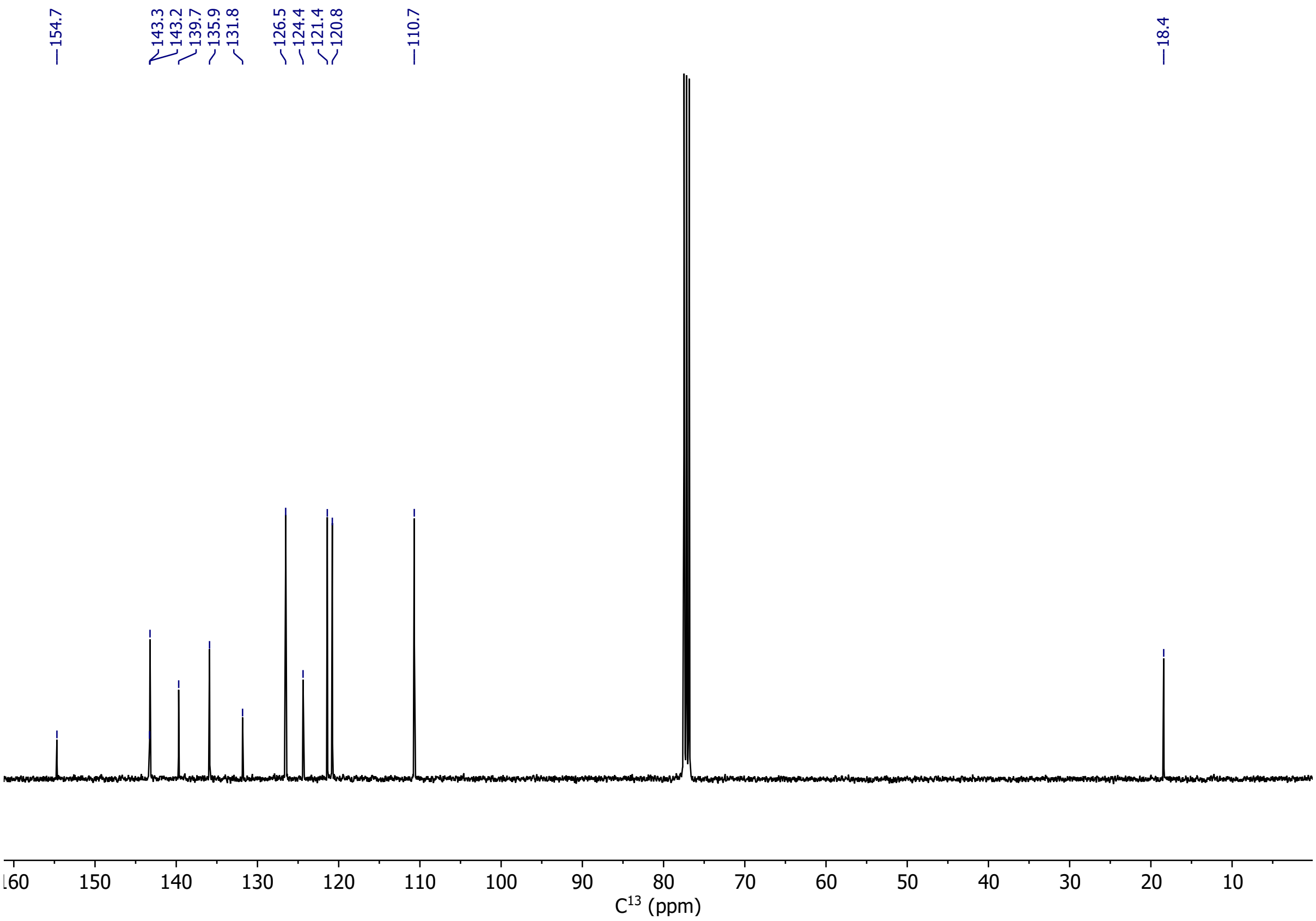

Figure S13: Compound **5** ^{13}C NMR spectrum.

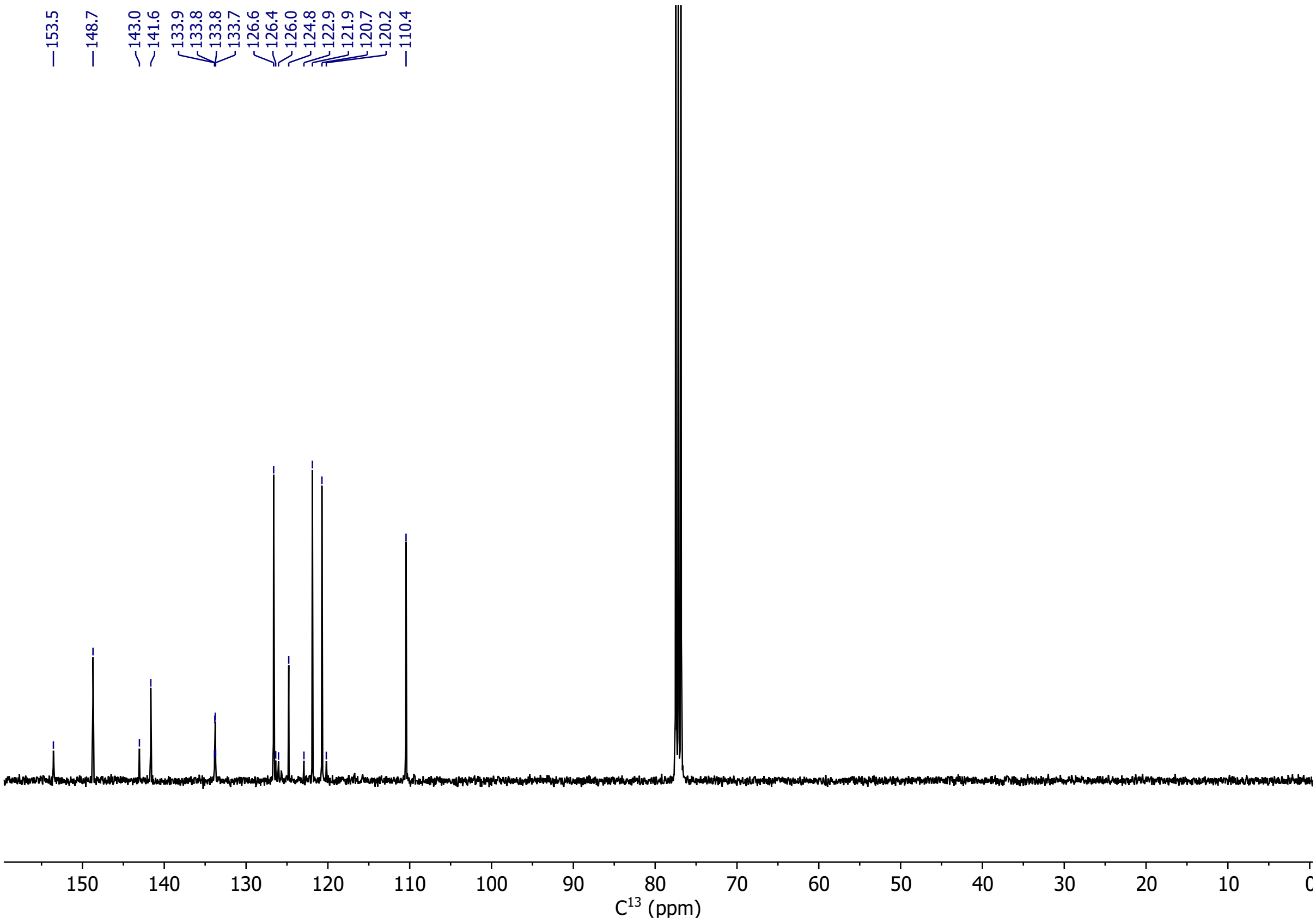

Figure S14: Compound **6** ^{13}C NMR spectrum.

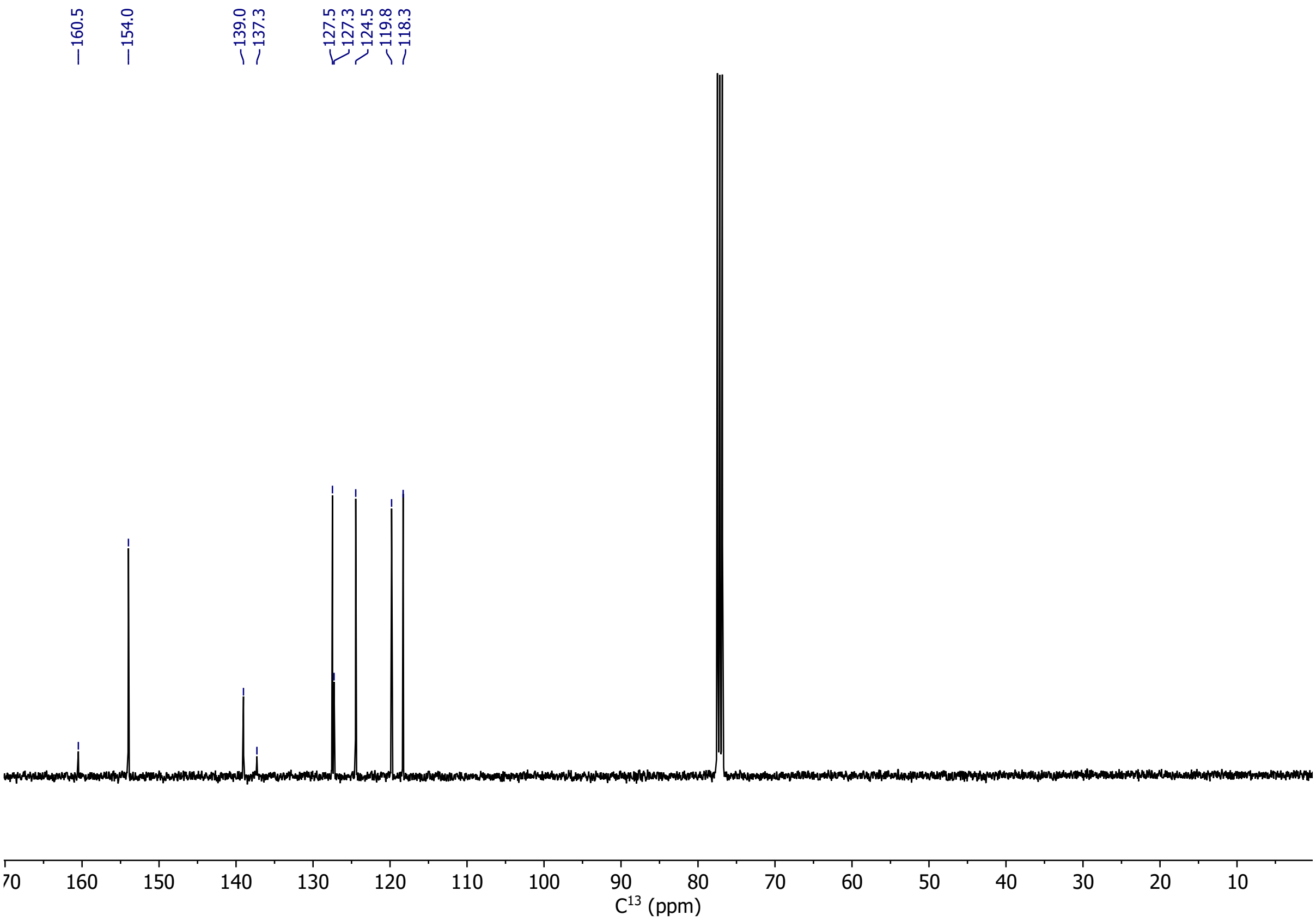

Figure S15: Compound **7** ^{13}C NMR spectrum.

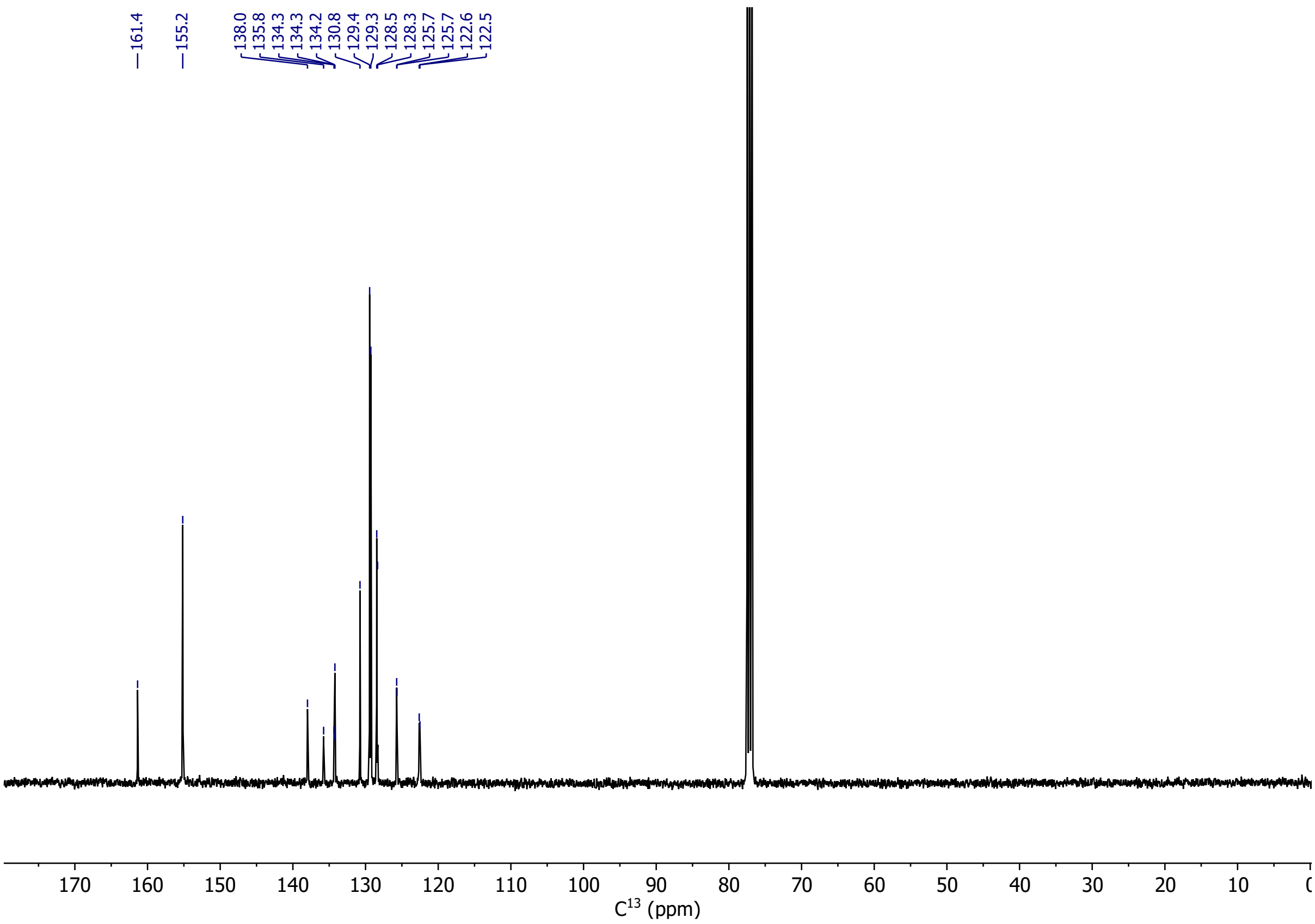

Figure S16: Compound **8** ^{13}C NMR spectrum.

Mass Spectra

Window Display Report

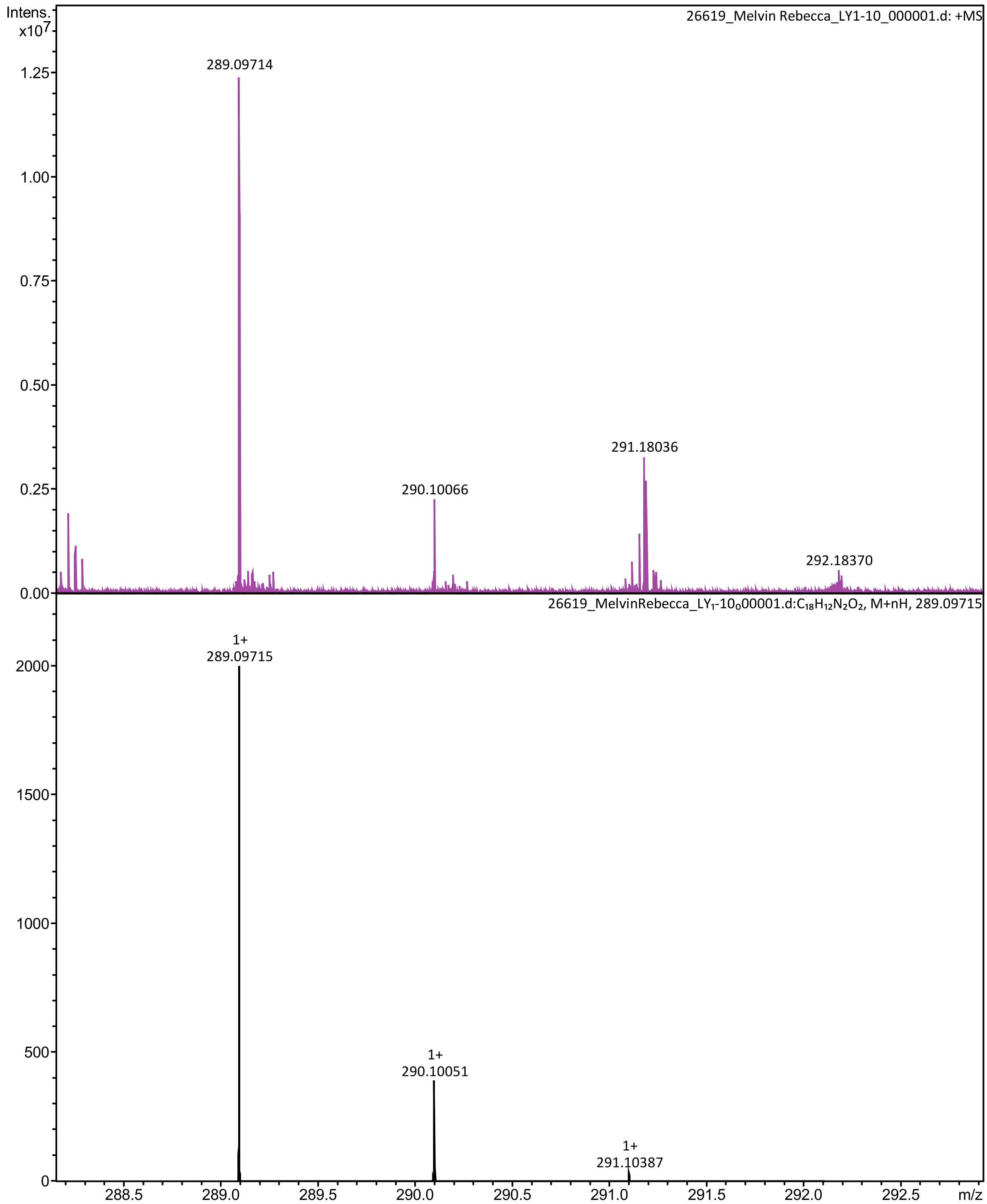

Bruker Compass DataAnalysis 4.4 printed: 30/01/2026 16:04:00 by: kgallag2 Page 1 of 1

Figure S17: Compound **1** mass spectrum.

Window Display Report

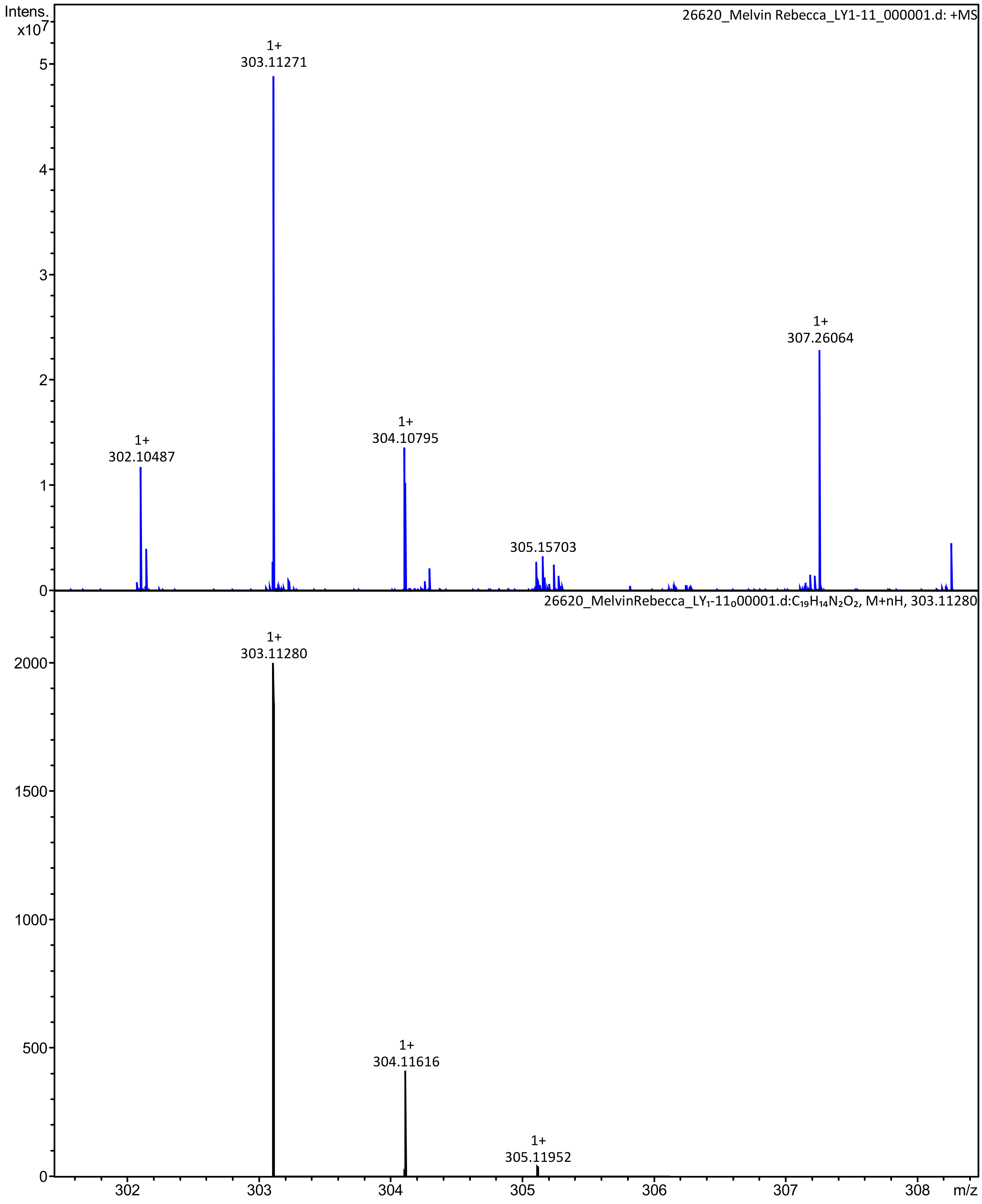

Bruker Compass DataAnalysis 4.4 printed: 30/01/2026 16:06:59 by: kgallag2 Page 1 of 1

Figure S18: Compound **2** mass spectrum.

Window Display Report

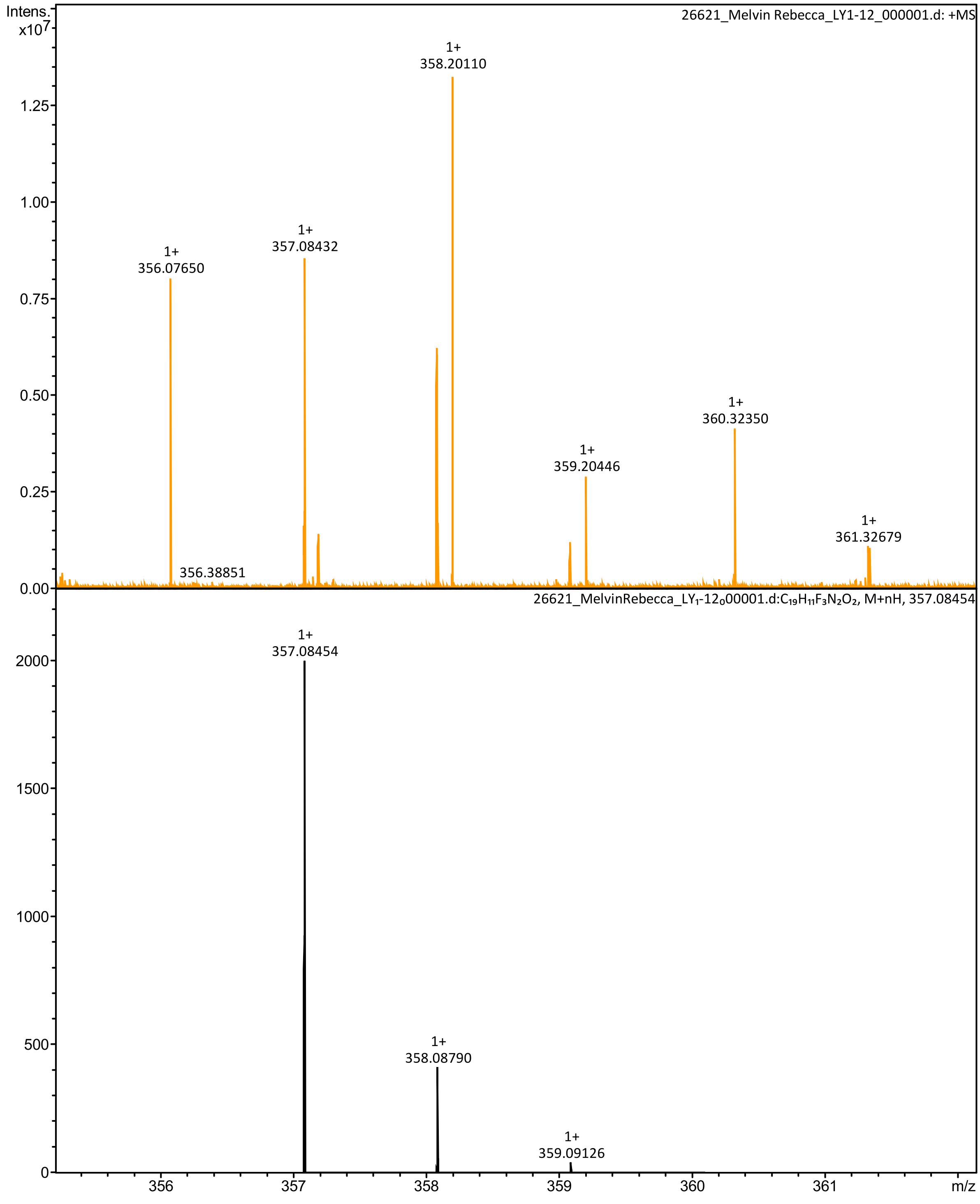

Bruker Compass DataAnalysis 4.4 printed: 30/01/2026 16:09:33 by: kgallag2 Page 1 of 1

Figure S19: Compound **3** mass spectrum.

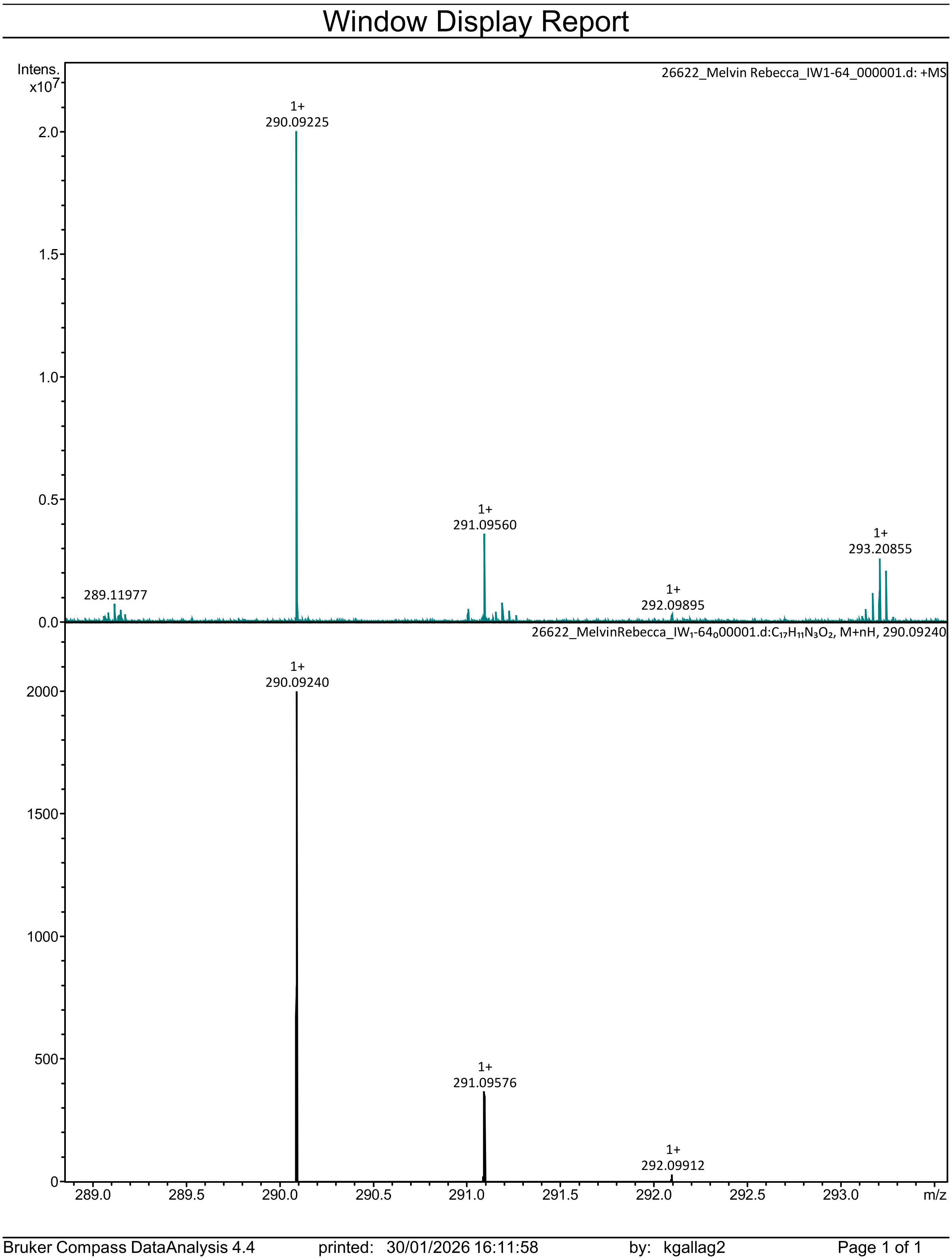

Figure S20: Compound **4** mass spectrum.

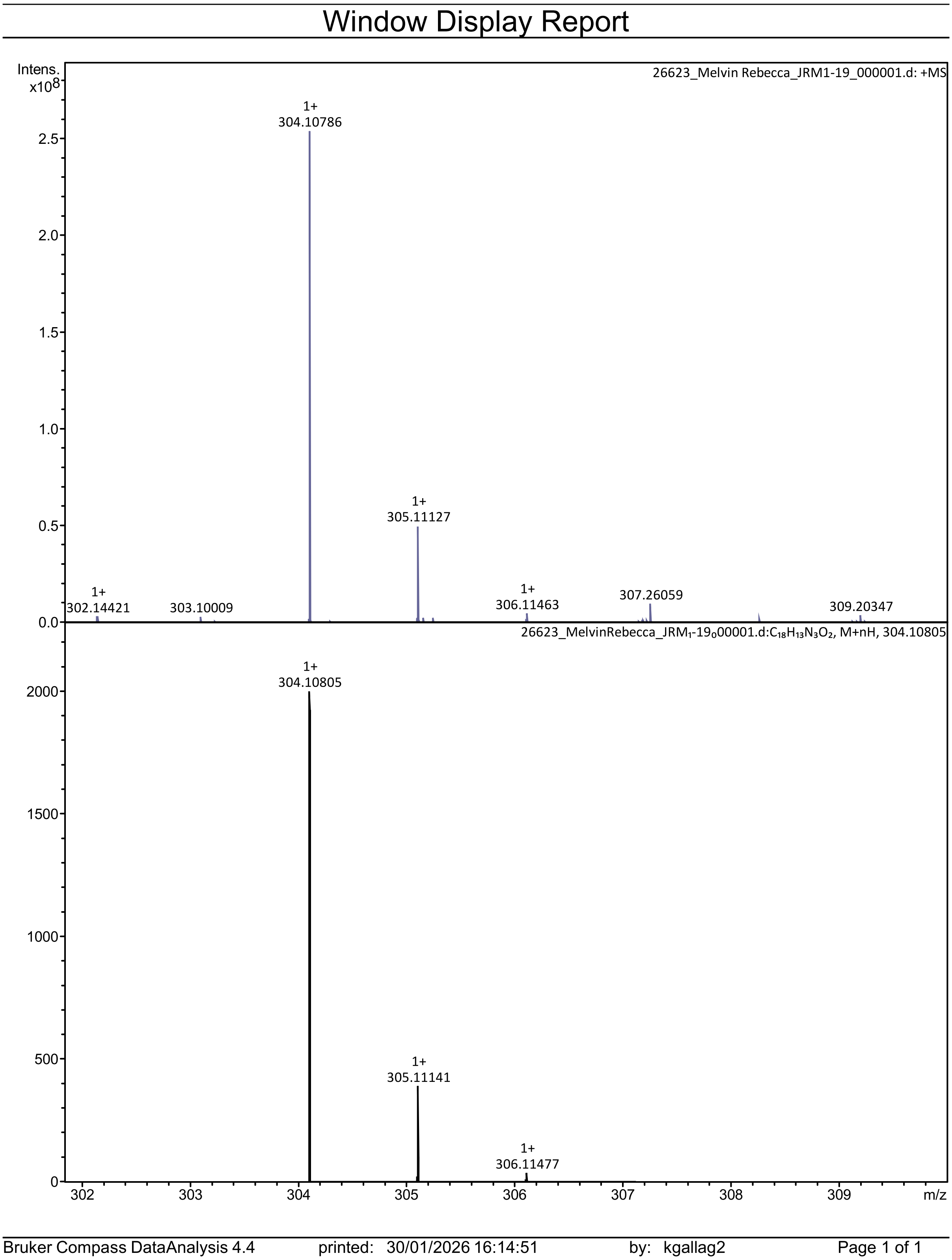

Figure S21: Compound **5** mass spectrum.

Window Display Report

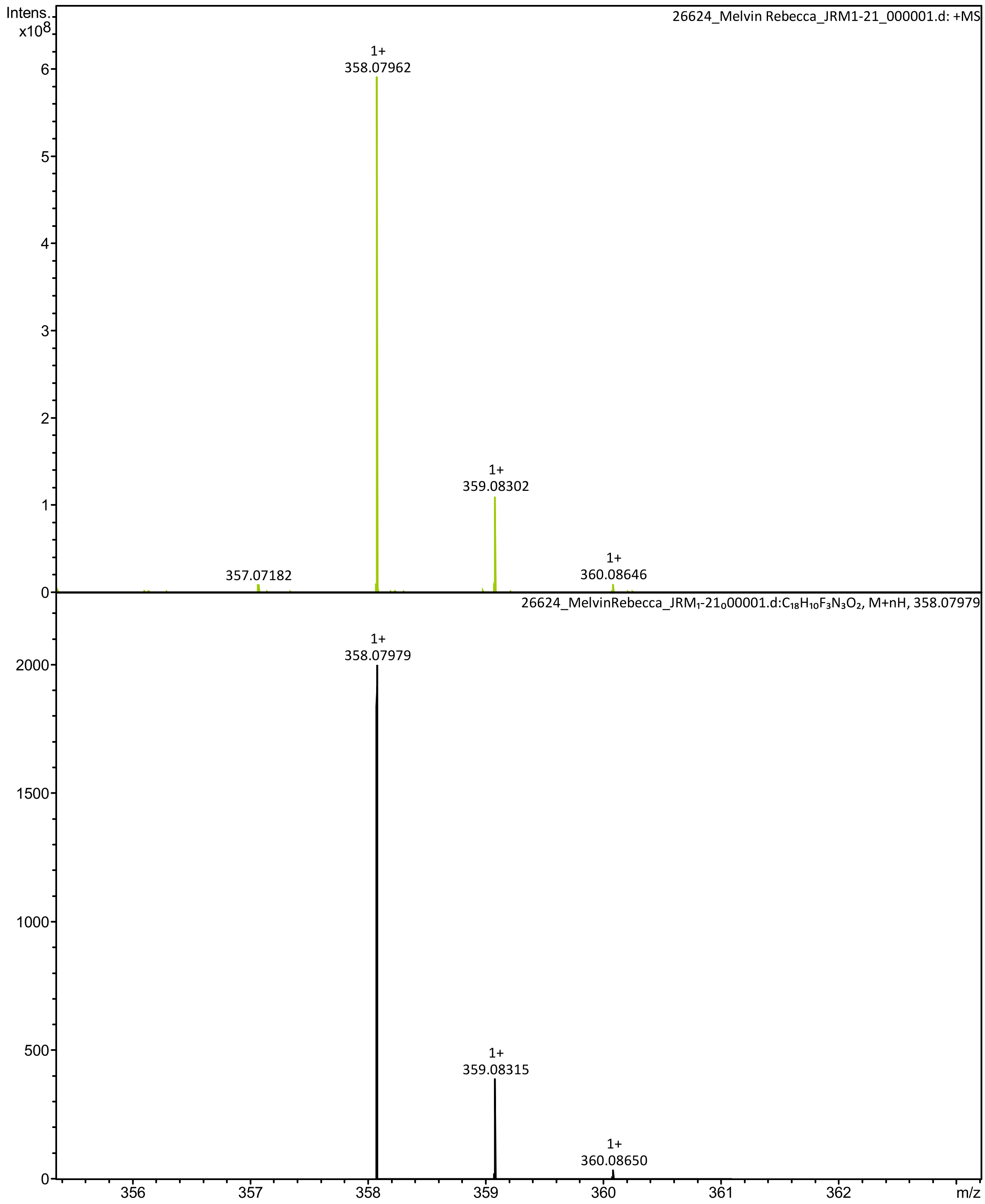

Bruker Compass DataAnalysis 4.4 printed: 30/01/2026 16:17:54 by: kgallag2 Page 1 of 1

Figure S22: Compound **6** mass spectrum.

Window Display Report

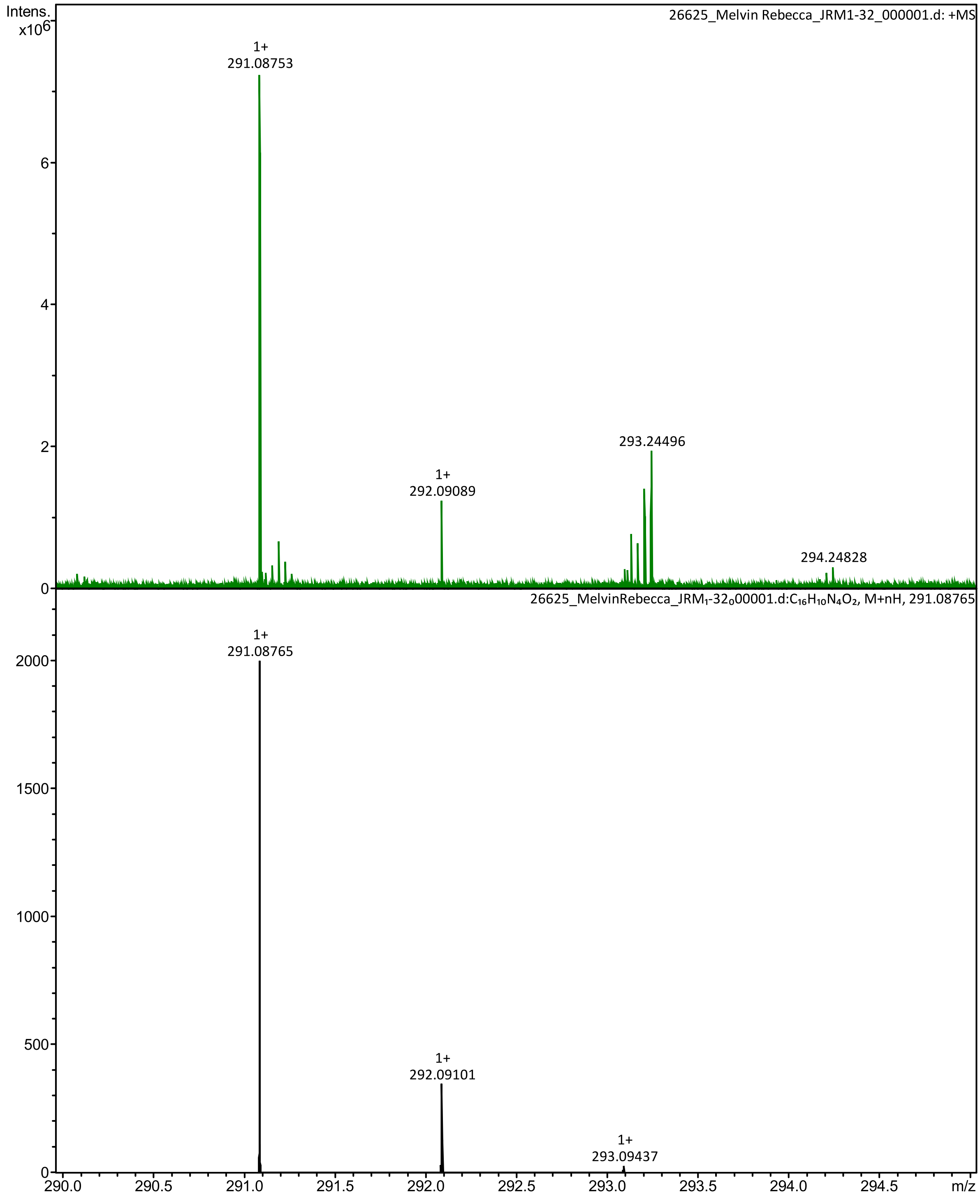

Bruker Compass DataAnalysis 4.4 printed: 30/01/2026 16:20:22 by: kgallag2 Page 1 of 1

Figure S23: Compound **7** mass spectrum.

Window Display Report

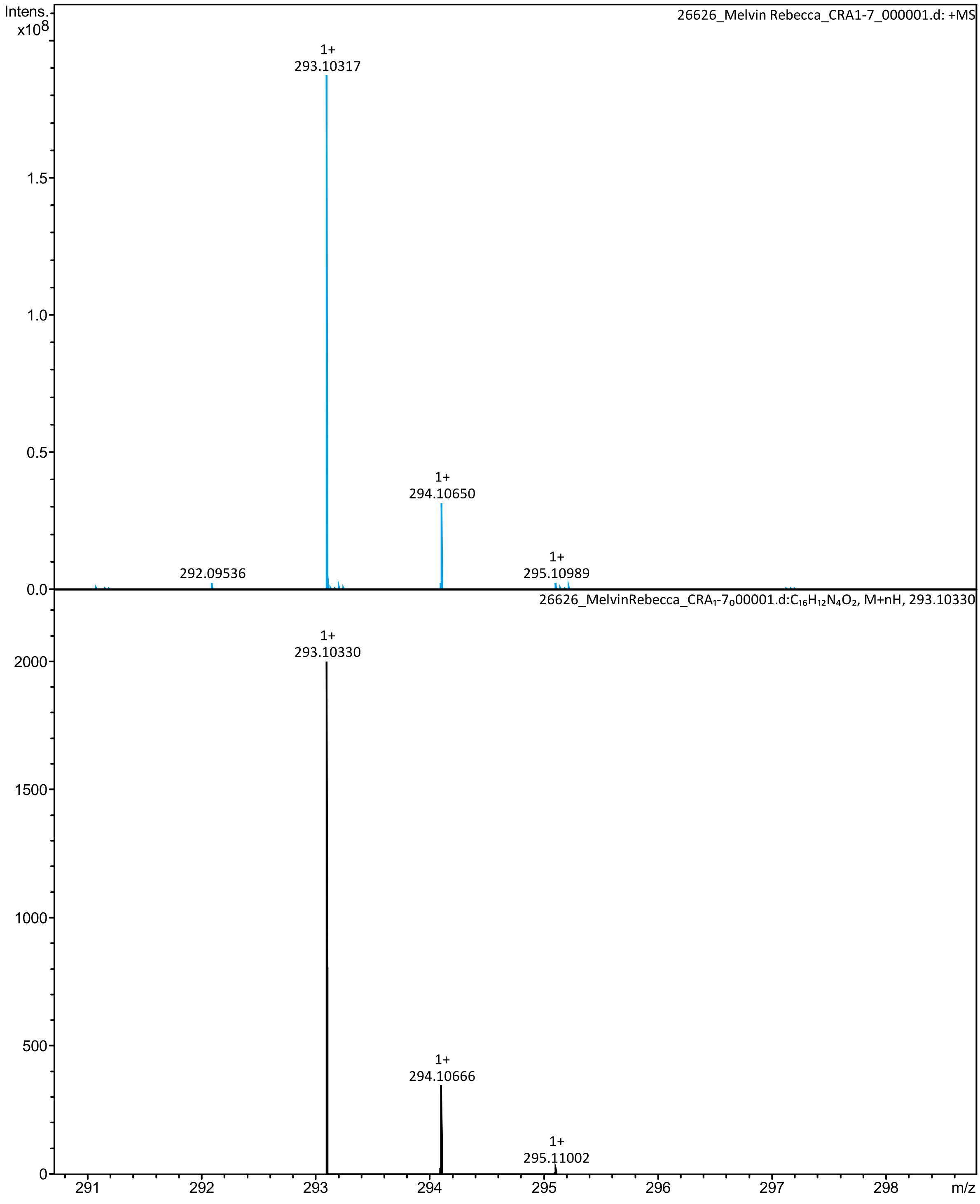

Bruker Compass DataAnalysis 4.4 printed: 30/01/2026 16:23:06 by: kgallag2 Page 1 of 1

Figure S24: Compound **8** mass spectrum.

X-Ray Crystallography

Diffraction data were collected at the ALS synchrotron, Station 12.2.1, with silicon 111-monochromated X-radiation, using a Bruker D8 diffractometer equipped with a Photon 100 detector for structures **1**, and **4**-**7**.[S4,S5] For **3**, a laboratory-based Rigaku 007HF diffractometer equipped with a HyPix 6000 detector and rotating anode source was used.[S6] The data were corrected for Lp effects and for absorption. The structures were solved and refined routinely.[S7,S8] H atom coordinates and U_{iso} parameters were freely refined for **1**, **3**, and **5**-**7** for which data quality was excellent, but placed geometrically for **4**, where it was not quite so good, probably due to the needle habit of the crystals. For non-centrosymmetric structures **4** {x = 0.3(2)} and **5** {x = –0.6(4)}, the lack of a heavy-enough anomalous scatterer meant the absolute structure parameter was poorly determined. Further details are presented in Tables S1 & S2 below.

CCDC 2402749-2402754 contain the supplementary crystallographic data for **1**, and **3**-**7**. These data can be obtained free of charge from The Cambridge Crystallographic Data Centre via www.ccdc.cam.ac.uk/structures.

Table S1: Crystallographic data for compounds **1** and **3-5**.

Compound	**1**	**3**	**4**	**5**
CCDC dep. no	2402749	2402750	2402751	2402752
Formula	$C_{18}H_{12}N_2O_2$	$C_{19}H_{11}F_3N_2O_2$	$C_{17}H_{11}N_3O_2$	$C_{18}H_{13}N_3O_2$
Formula weight	288.30	2300.61	289.29	303.31
Crystal system	Monoclinic	Triclinic	Orthorhombic	Orthorhombic
Space group	$P2_1/c$	$P\bar{1}$	$P2_12_12_1$	$P2_12_12_1$
a (Å)	8.2254(10)	8.59135(15)	11.9605(5)	7.5074(3)
b (Å)	21.265(3)	12.61480(14)	16.2743(6)	10.2416(4)
c (Å)	8.1217(10)	15.0464(2)	6.9237(3)	18.6642(8)
α (º)	90	103.1691(12)	90	90
β (º)	105.426(5)	91.5816(14)	90	90
γ (º)	90	93.7764(12)	90	90
V (Å^3)	1369.4(3)	1582.84(4)	1347.69(10)	1435.05(10)
Z	4	4	4	4
Temperature (K)	100(2)	100(2)	100(2)	100(2)
Wavelength, λ (Å)	0.7288	1.54178	0.7288	0.7288
Calculated density (g.cm^{-3})	1.398	1.495	1.426	1.404
Absorption coefficient, μ (mm^{-1})	0.10	1.05	0.10	0.10
Crystal size (mm^3)	0.17 × 0.10 × 0.07	0.19 × 0.10 × 0.03	0.23 × 0.01 × 0.01	0.30 × 0.20 × 0.18
θ(max) (°)	28.3	68.2	29.2	31.4
Reflections measured	38936	29047	43189	26670
Unique reflections	3152	5750	3366	4393
Reflections with $I > 2σ(I)$	2811	5175	3254	4228
R_{int}	0.089	0.041	0.049	0.053
Number of parameters	247	557	200	261
R_1 [$F^2 > 2σ(F^2)$]	0.042	0.035	0.058	0.040
wR_2 (all data)	0.113	0.096	0.138	0.095
GOOF, S	1.04	1.06	1.25	1.08
Largest difference peak and hole (e Å^{-3})	0.33 and –0.30	0.23 and –0.27	0.23 and –0.29	0.43 and –0.41

Table S2. Crystallographic data for compounds **6** and **7**.

Compound	**6**	**7**
CCDC dep. no	2402753	2402754
Formula	$C_{18}H_{10}F_3N_3O_2$	$C_{16}H_{10}N_4O_2$
Formula weight	357.29	290.28
Crystal system	Monoclinic	Monoclinic
Space group	$P2_1/c$	$P2_1/n$
a (Å)	7.3708(3)	14.1867(9)
b (Å)	18.4047(8)	5.8319(4)
c (Å)	22.1303(10)	16.1091(10)
α (°)	90	90
β (°)	91.758(2)	104.523(2)
γ (°)	90	90
V (Å^3)	3000.7(2)	1290.21(15)
Z	8	4
Temperature (K)	100(2)	100(2)
Wavelength, λ (Å)	0.7288	0.7288
Calculated density (g.cm^{-3})	1.582	1.494
Absorption coefficient, μ (mm^{-1})	0.14	0.11
Crystal size (mm^3)	0.30 × 0.02 × 0.02	0.18 × 0.02 × 0.02
θ(max) (°)	31.4	26.4
Reflections measured	107132	33171
Unique reflections	9195	2459
Reflections with $I > 2\sigma(I)$	8400	2046
R_{int}	0.058	0.081
Number of parameters	550	240
R_1 [$F^2 > 2\sigma(F^2)$]	0.037	0.033
wR_2 (all data)	0.102	0.086
GOOF, S	1.05	1.05
Largest difference peak and hole (e Å^{-3})	0.56 and –0.32	0.25 and –0.19

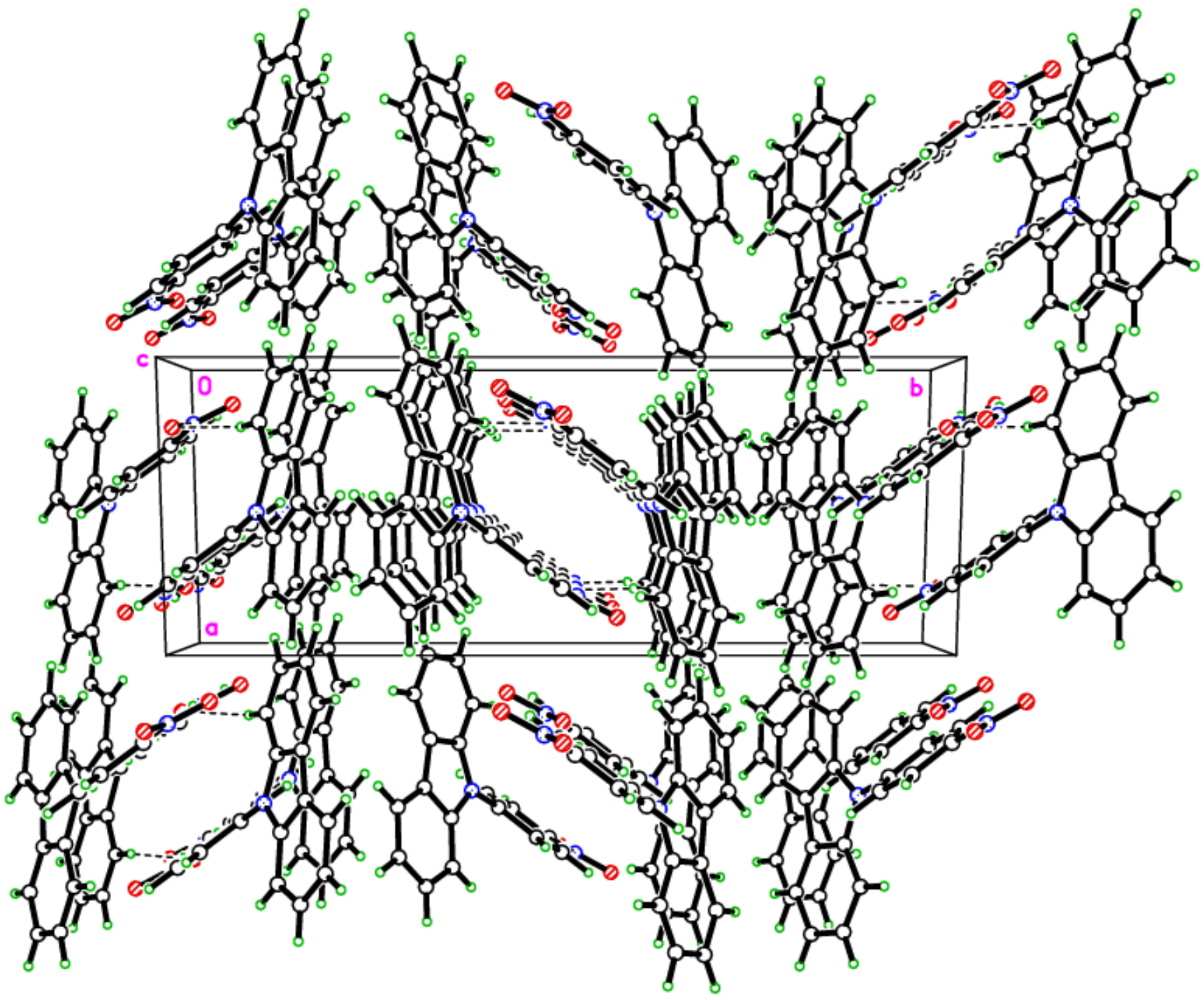

Figure S25 Crystal packing for **1** viewed down the *c*-axis. Interactions between one of the O atoms of the –NO_2 groups with the $H^{1/8}$ atoms of the carbazole in the nearest adjacent stack are shown as dashed lines.

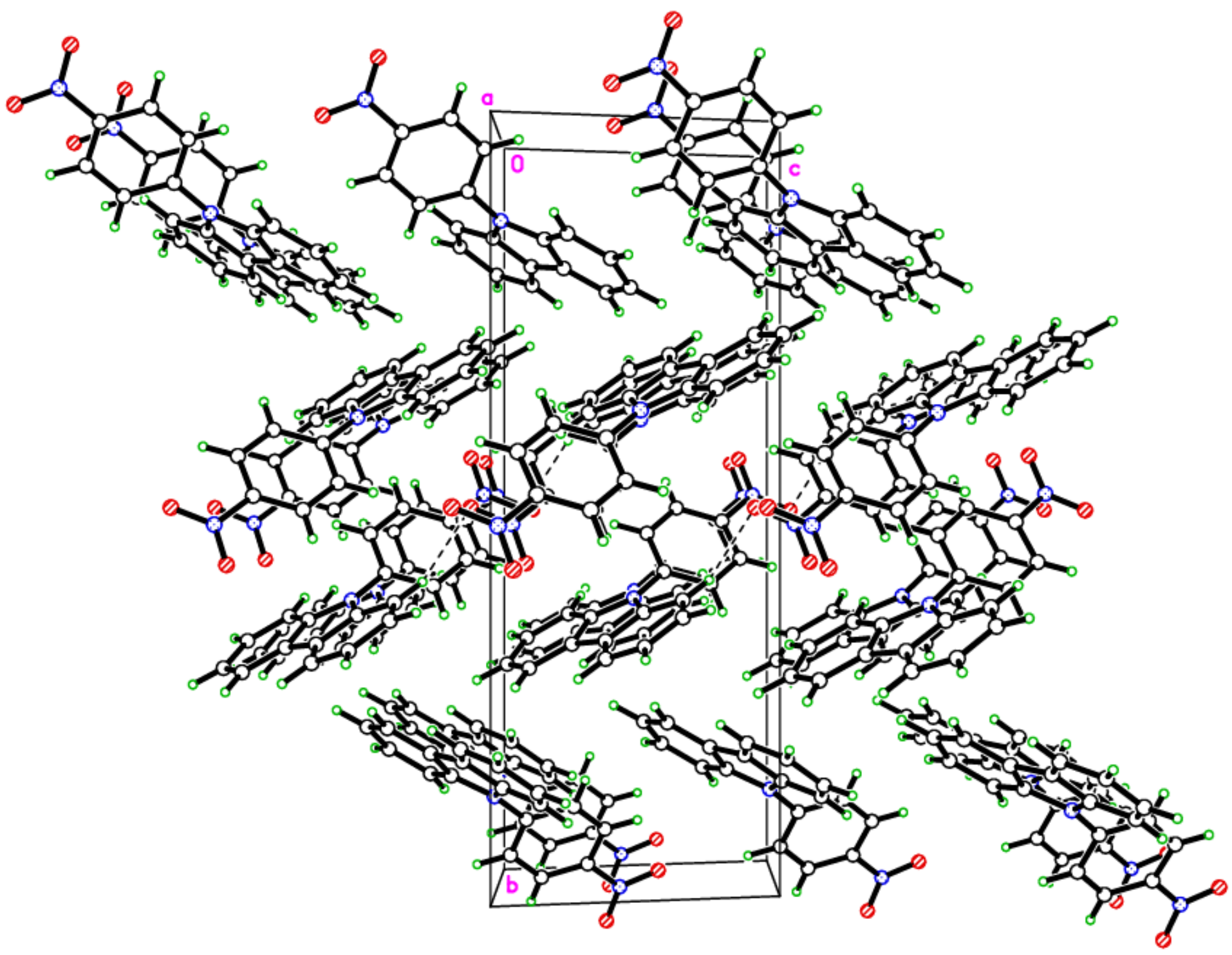

Figure S26 Crystal packing for **1** viewed down the *a*-axis. Interactions between the –NO_2 groups with the $H^{1/8}$ atoms of the carbazole in the nearest adjacent stack are shown as dashed lines.

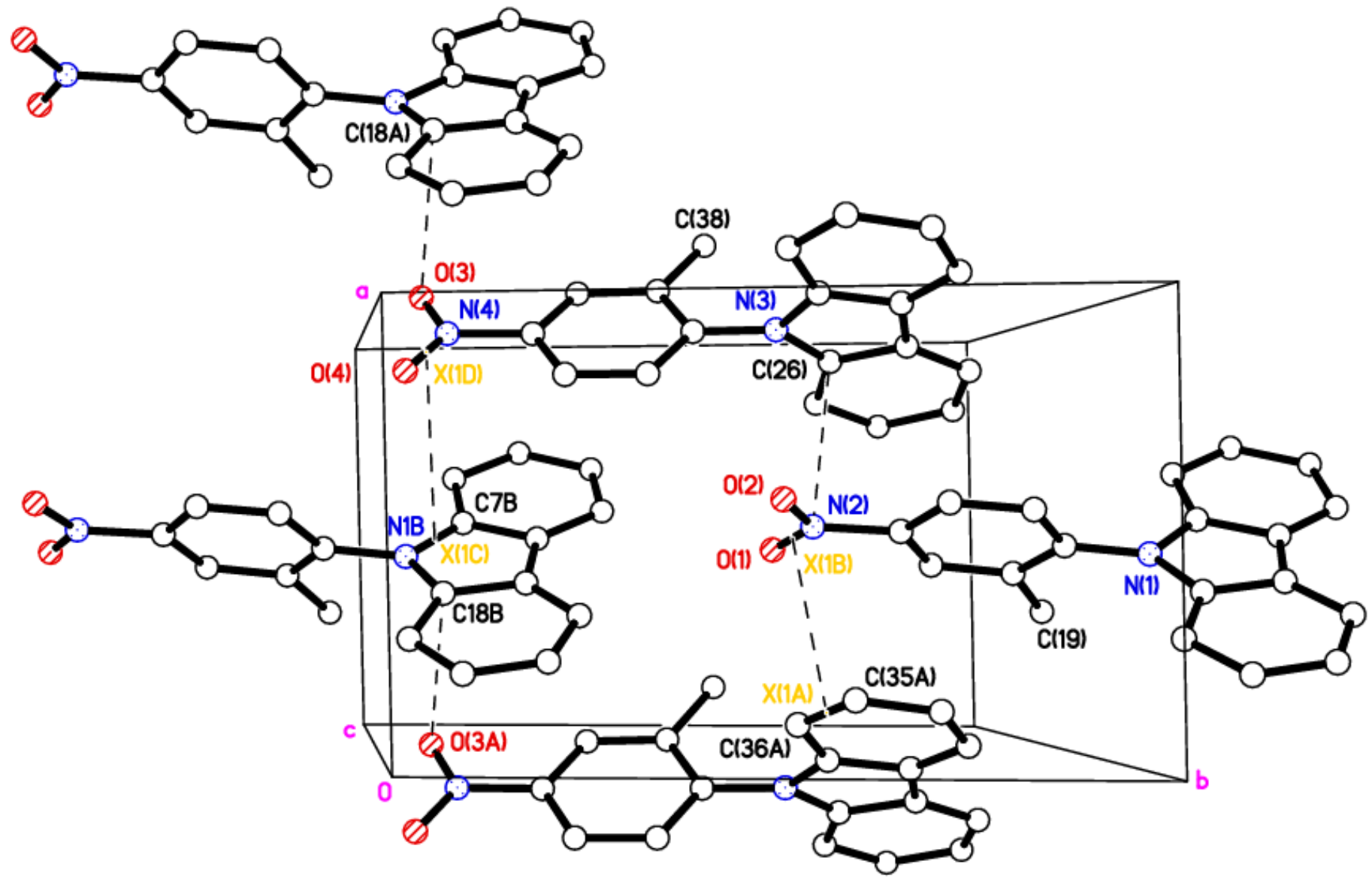

Figure S27 Crystal packing for **2** viewed down the *c*-axis. Face-to-face stacking of the donor and acceptor regimes of adjacent molecules is supported by interactions between the –NO_2 groups and the carbazole rings and shown as dashed lines. H-atoms have been omitted for clarity.

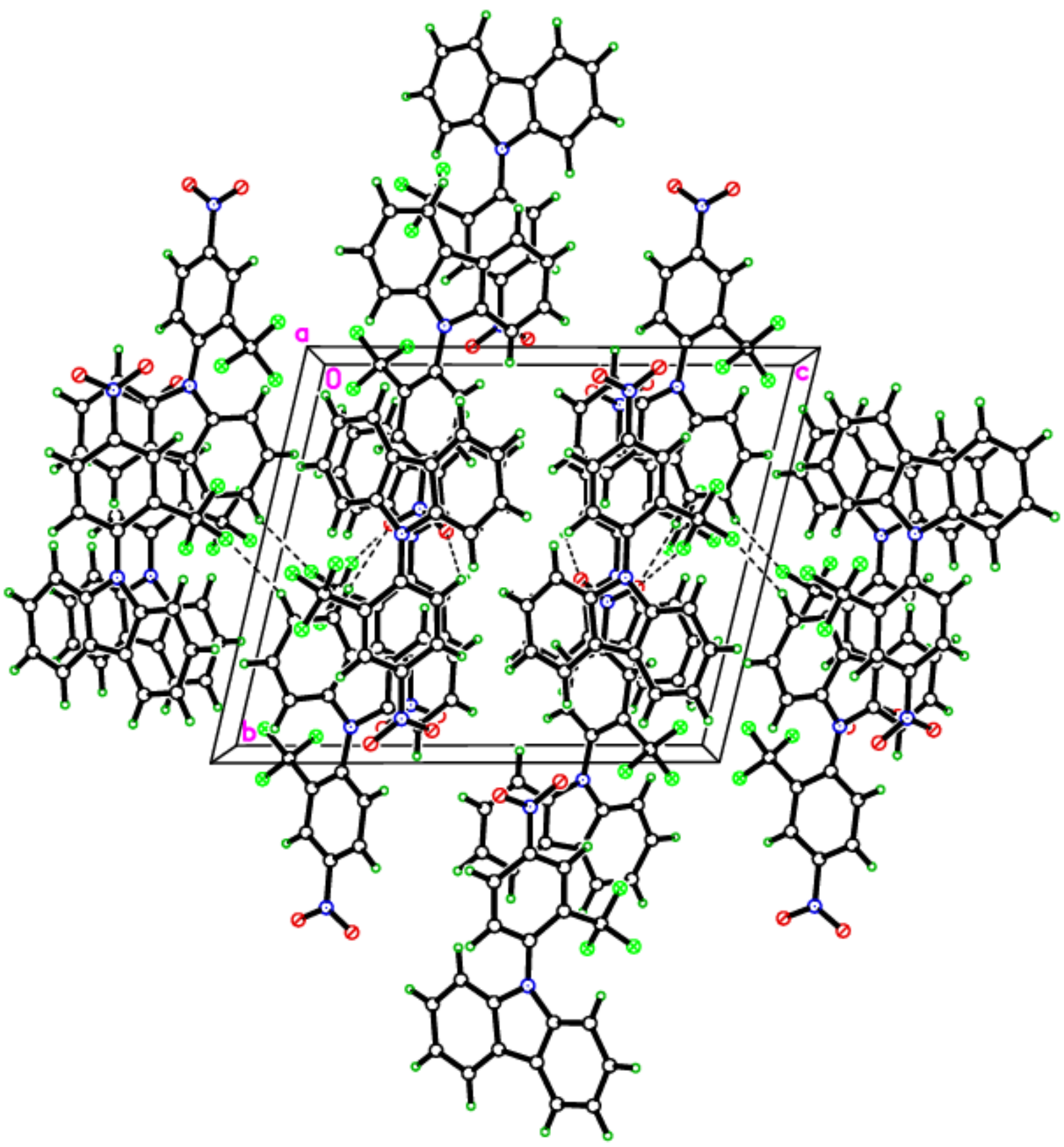

Figure S28 Crystal packing for **3** viewed down the *a*-axis. Intermolecular interactions between the –NO_2 groups with the $H^{6'}$ atoms of the aryl ring and between F and $H^{3/6}$ of the carbazole are shown as dashed lines.

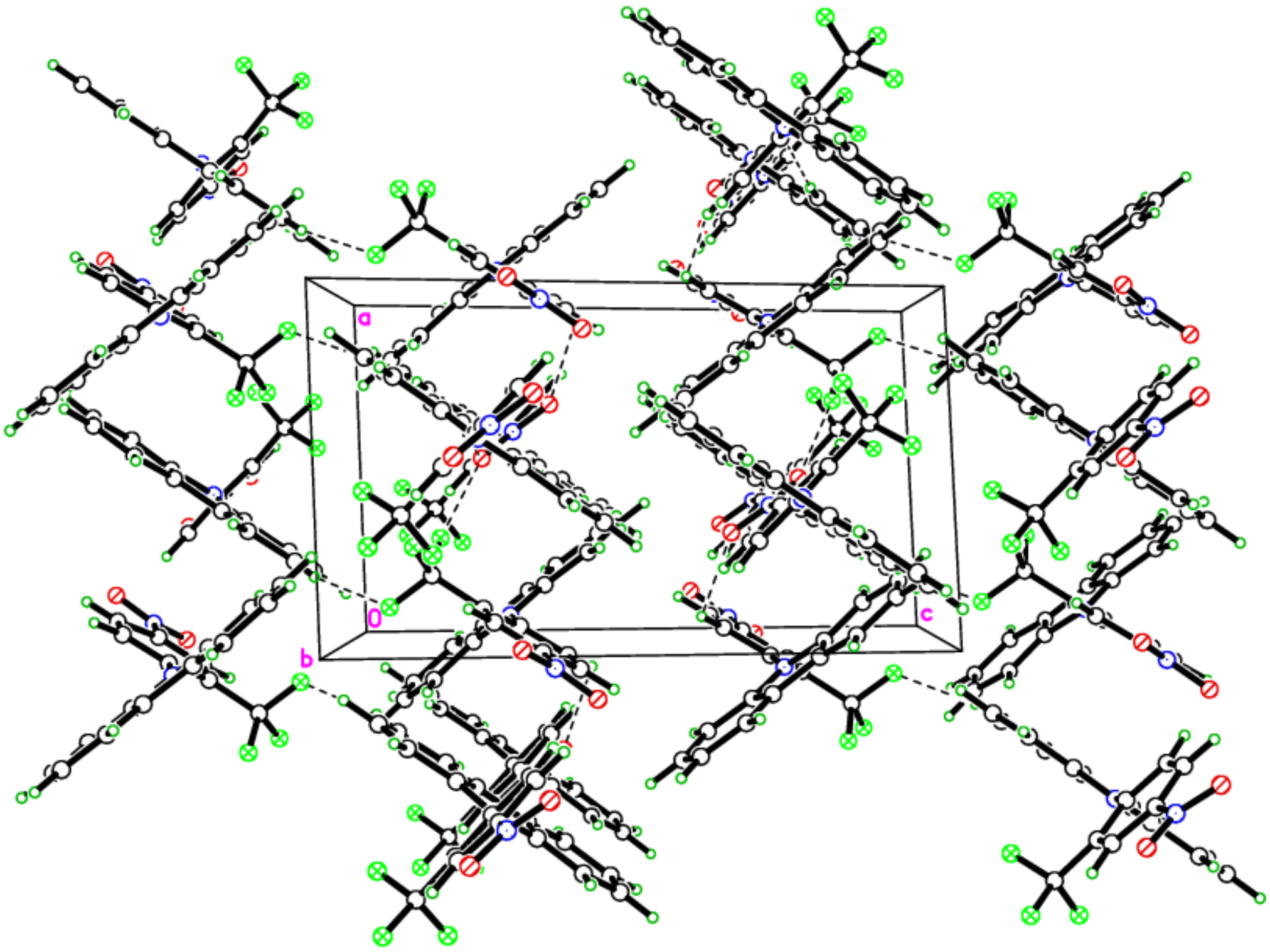

Figure S29 Crystal packing for **3** viewed down the *b*-axis. Intermolecular interactions between the –NO_2 groups with the $H^{6'}$ atoms of the aryl ring and between F and $H^{3/6}$ of the carbazole are shown as dashed lines.

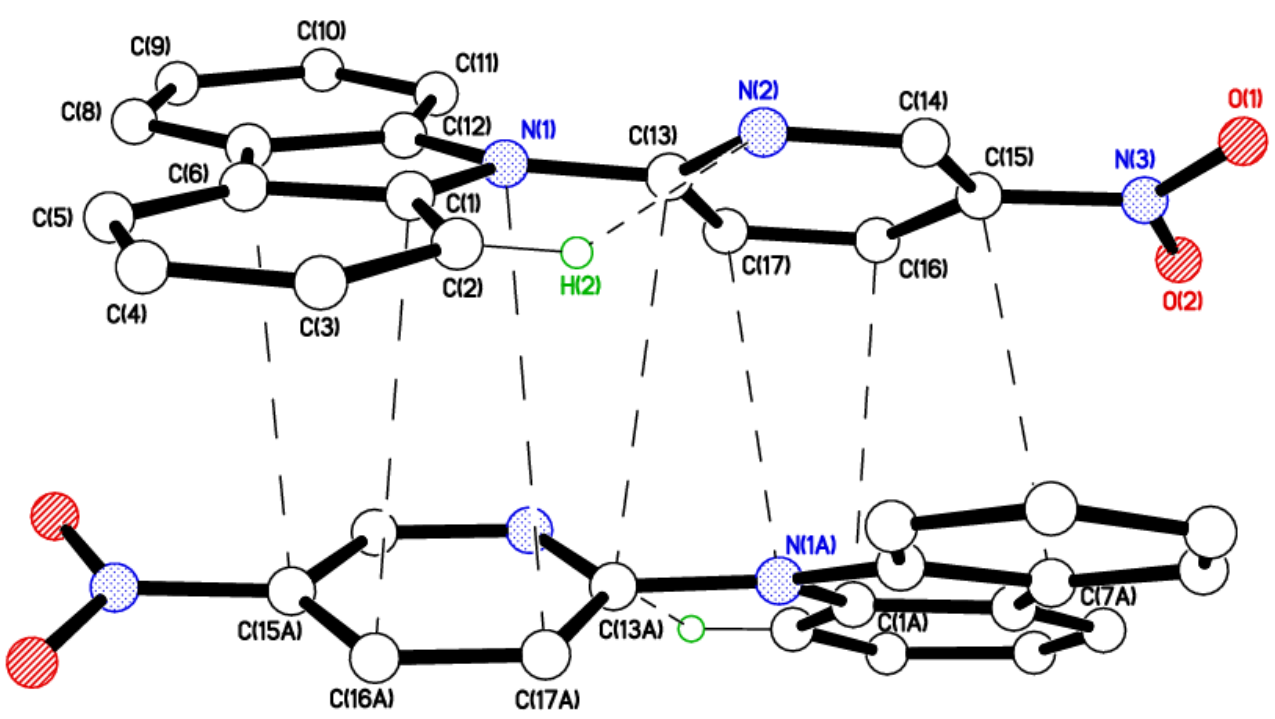

Figure S30 Face-to-face π-stacking of the donor and acceptor regimes of two adjacent molecules of **4**. The intramolecular hydrogen bond (IHB) is shown as a dashed line between H(2/2A) and N(2/2A). Other H-atoms have been omitted for clarity.

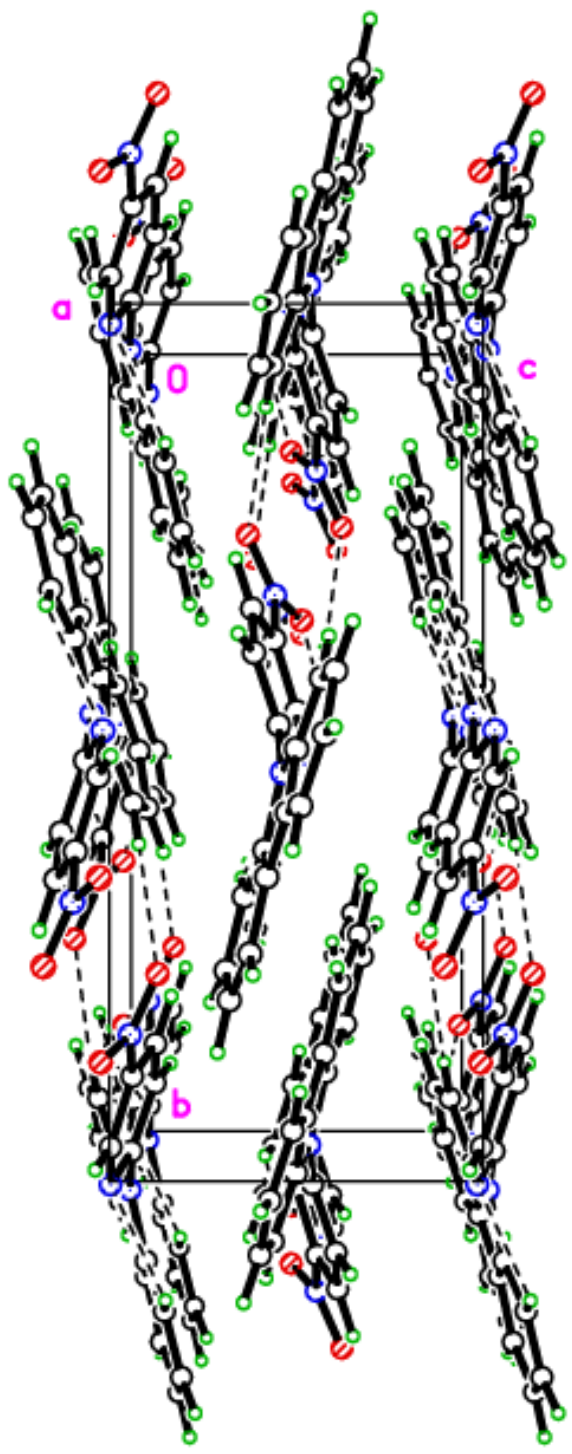

Figure S31 Crystal packing for **4** viewed down the *a*-axis. Intermolecular interactions between the –NO_2 groups and carbazole $H^{1/8}$ atoms of neighbouring molecules are shown as dashed lines.

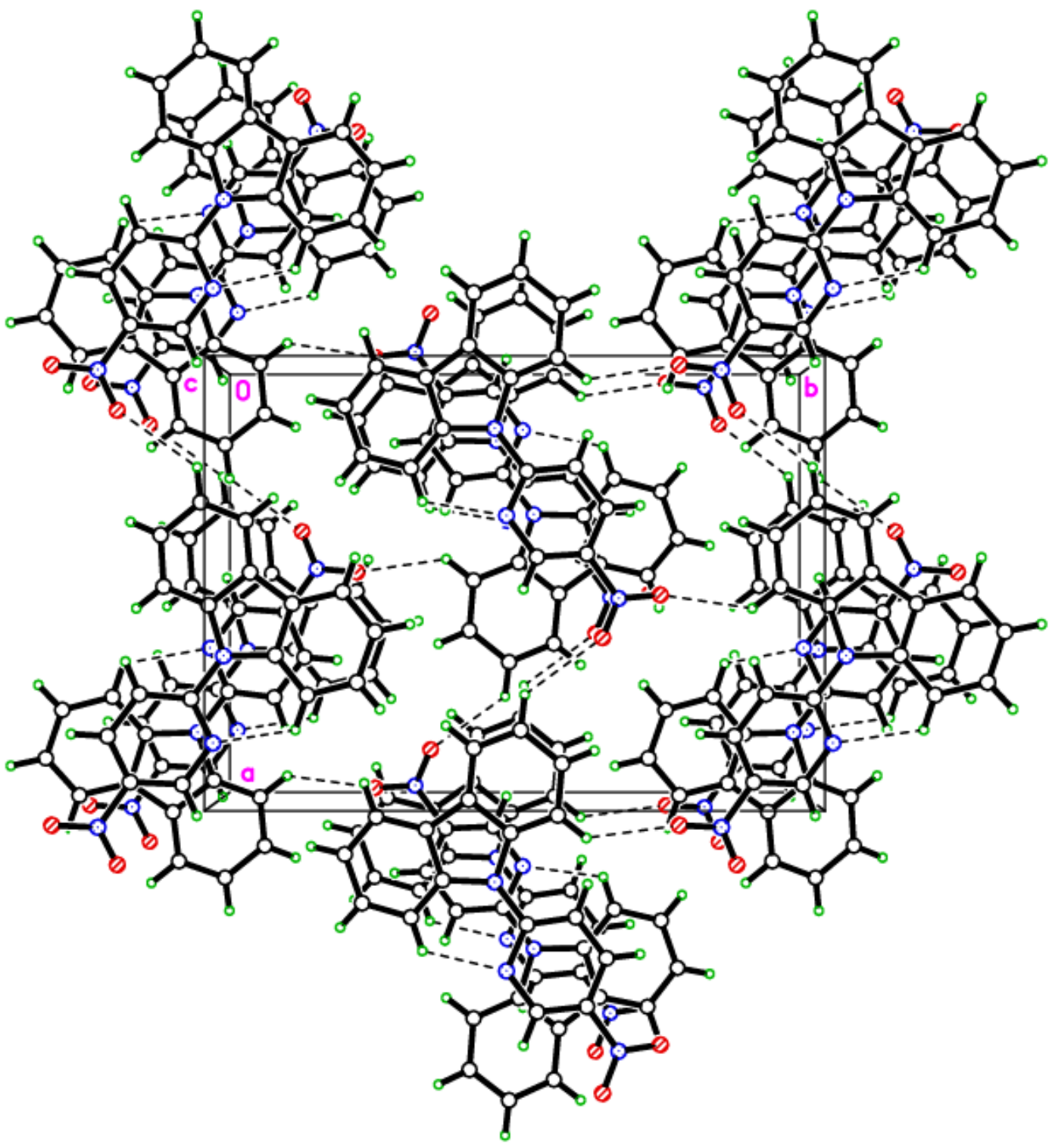

Figure S32 Crystal packing for **4** viewed down the *c*-axis. Intermolecular interactions between the –NO_2 groups and carbazole $H^{1/8}$ atoms of neighbouring molecules are shown as dashed lines.

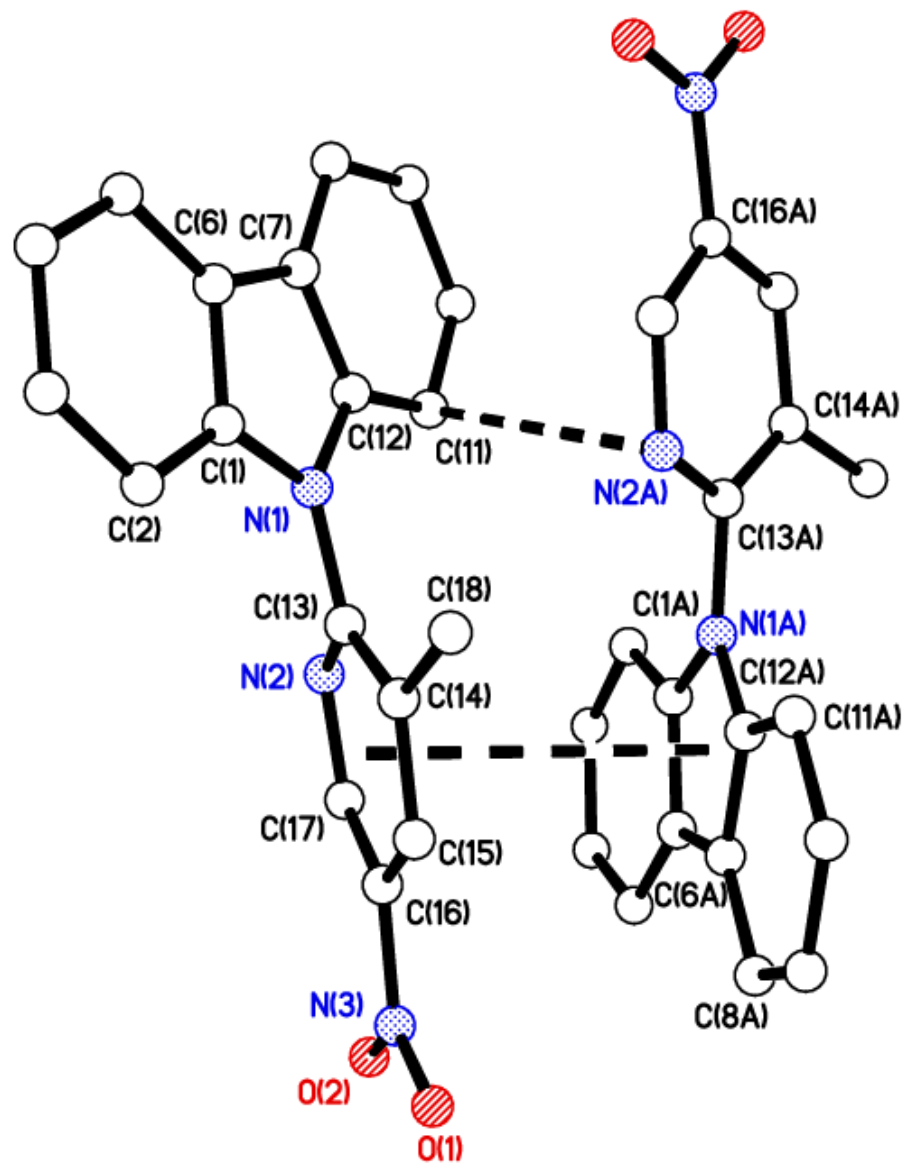

Figure S33 Face-to-face π-stacking of the donor and acceptor regimes of two adjacent molecules of **5**. H-atoms have been omitted for clarity.

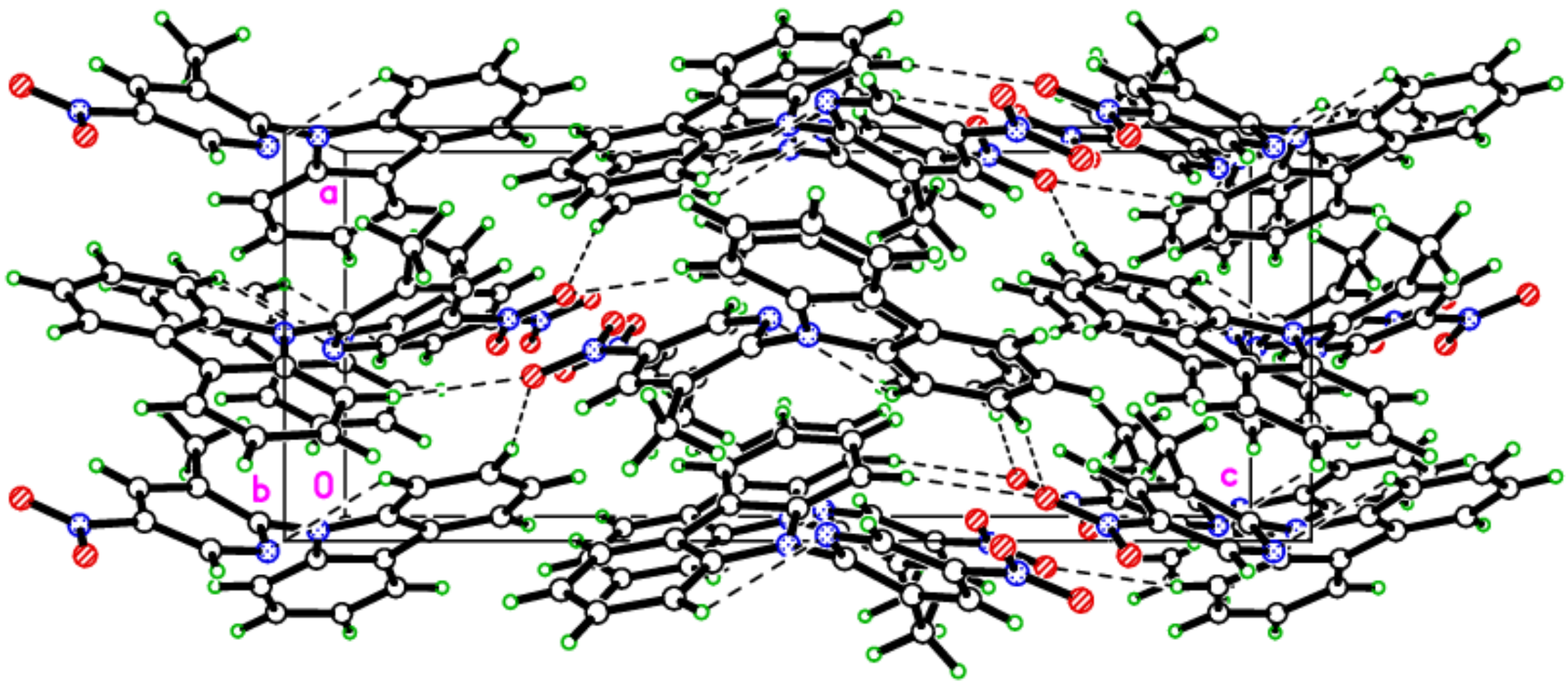

Figure S34 Crystal packing for **5** viewed down the *b*-axis The apparent IHB between the N atom of the pyridine and H^1 of the carbazole, and intermolecular interactions between the $–NO_2$ groups and the carbazole H^8 and H^2 atoms of neighbouring molecules are shown as dashed lines.

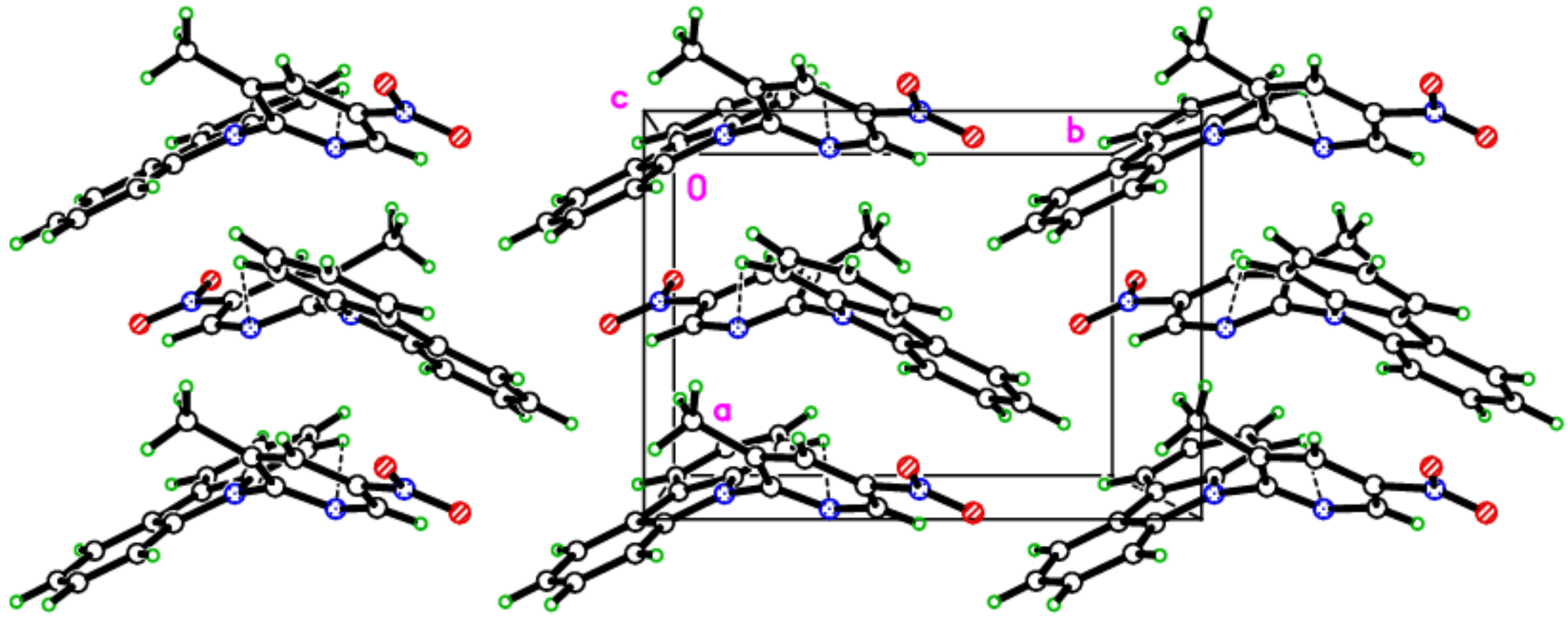

Figure S35 Crystal packing for **5** viewed down the *c*-axis. The apparent IHB between the N atom of the pyridine and H^1 of the carbazole are shown as dashed lines.

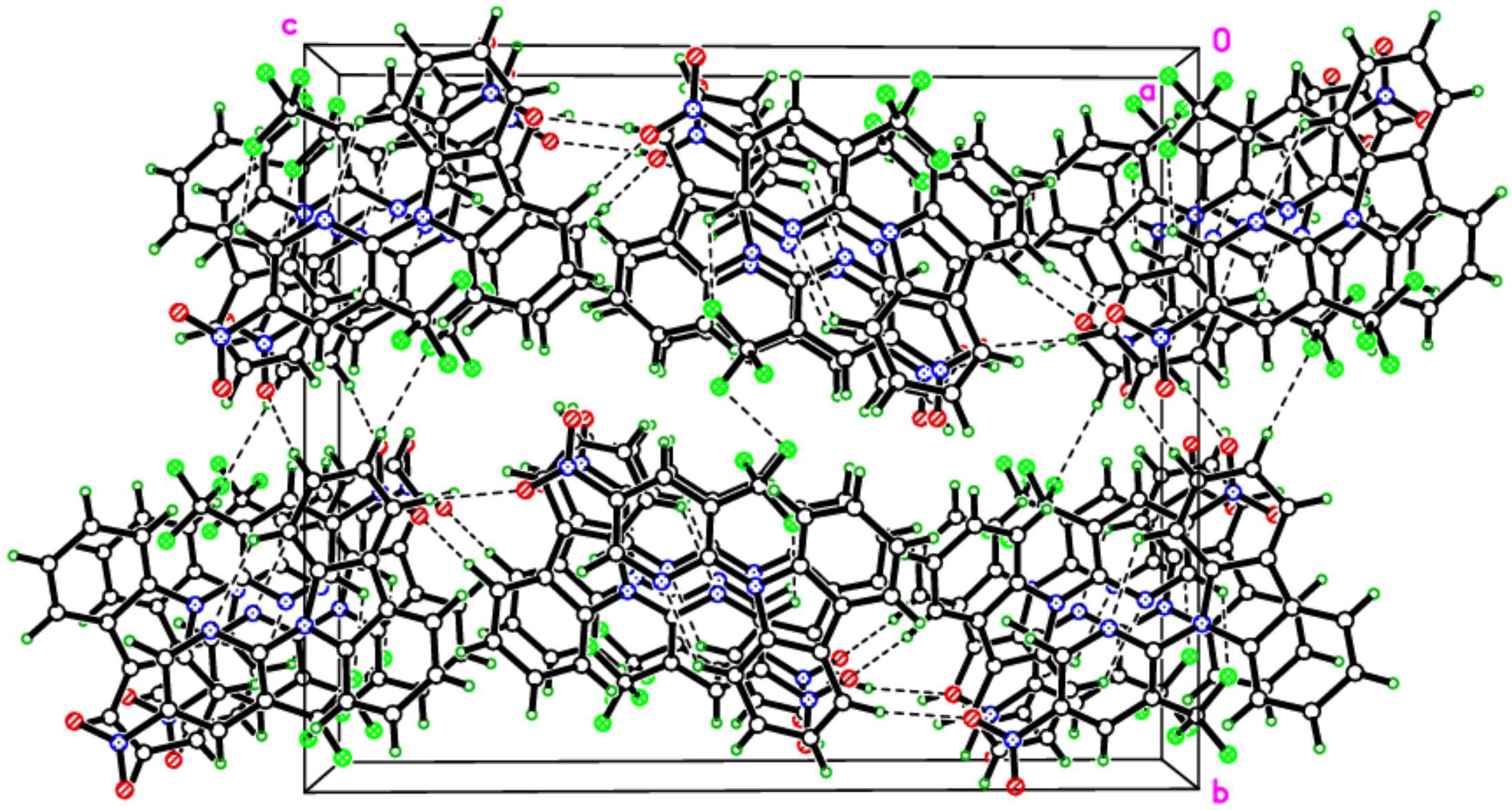

Figure S36 Crystal packing for **6** viewed down the *a*-axis. The apparent IHB between the N atom of the pyridine and H^1 of the carbazole, and intermolecular interactions between the $–NO_2$ groups and carbazole H^4 and H^5 atoms, and between F and H^3 of the carbazole are shown as dashed lines of neighbouring molecules are shown as dashed lines.

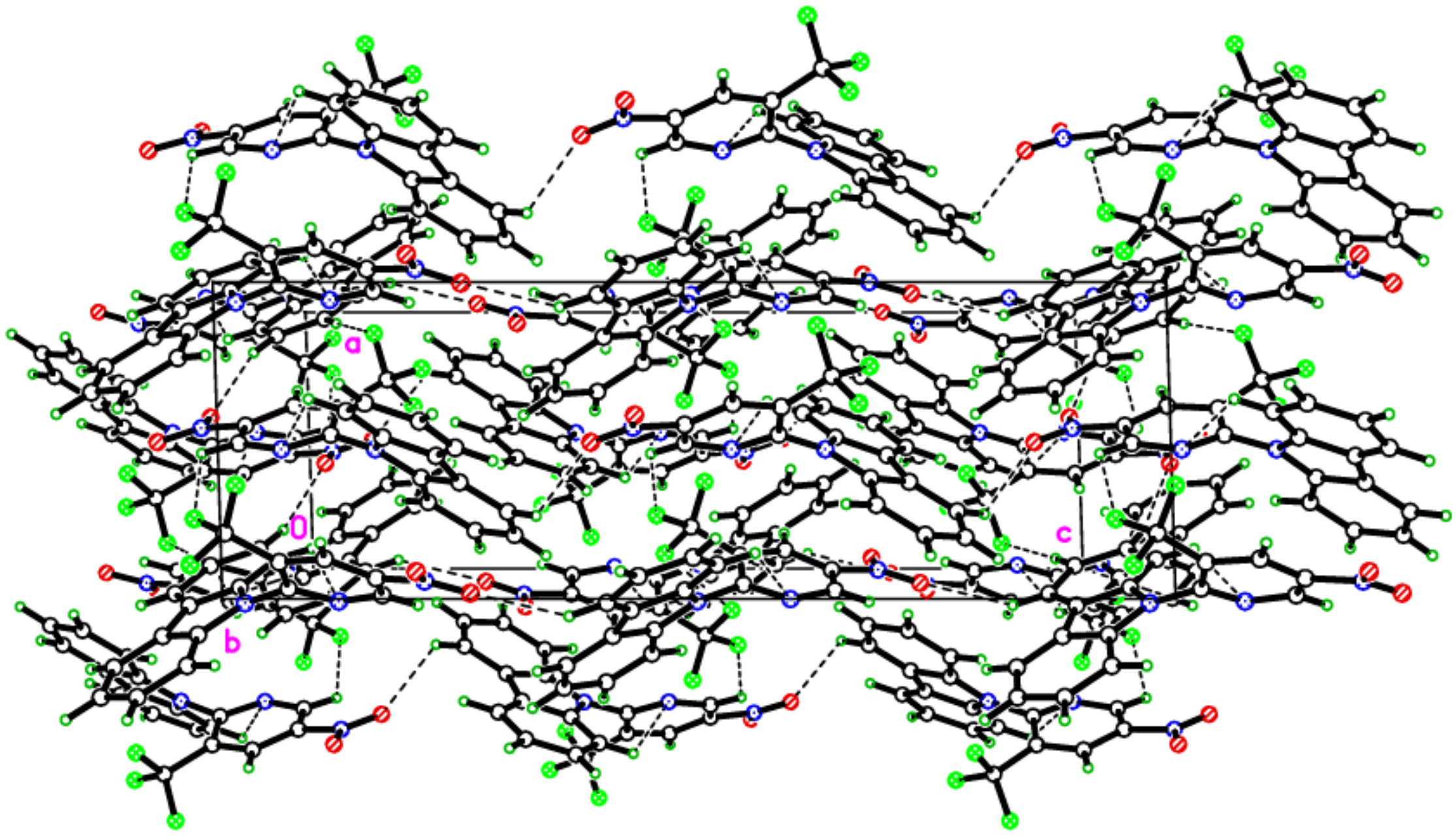

Figure S37 Crystal packing for **6** viewed down the *b*-axis. The apparent IHB between the N atom of the pyridine and H[1] of the carbazole, and intermolecular interactions between the $–NO_2$ groups and carbazole H[4] and H[5] atoms, and between F and H[3] of the carbazole are shown as dashed lines of neighbouring molecules are shown as dashed lines.

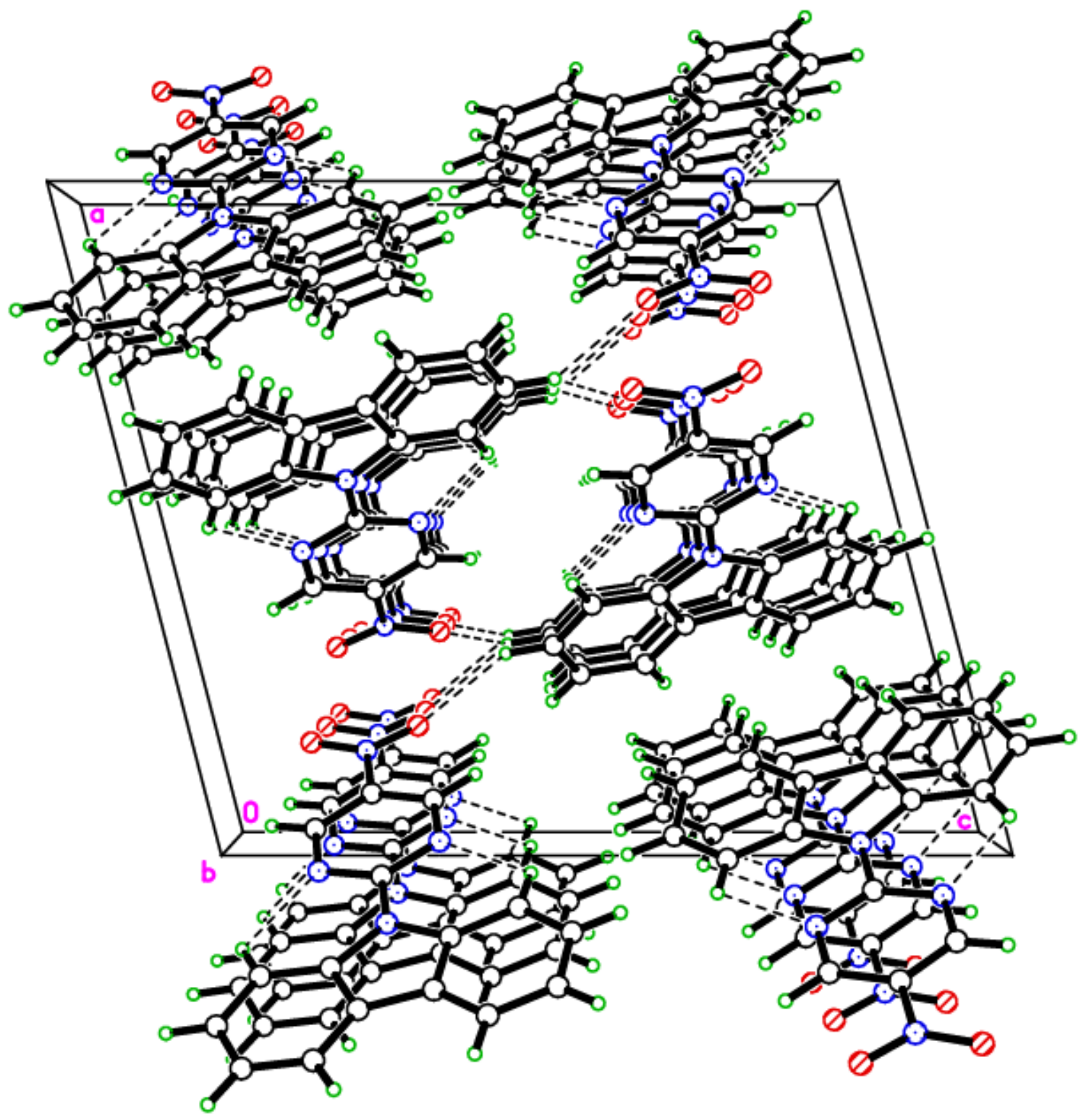

Figure S38 Crystal packing for **7** viewed down the *b*-axis. The IHB between the N atom of the pyrimidine and H[1/8] of the carbazole, and intermolecular interactions between the $–NO_2$ groups and carbazole H[2/7] atoms are shown as dashed lines.

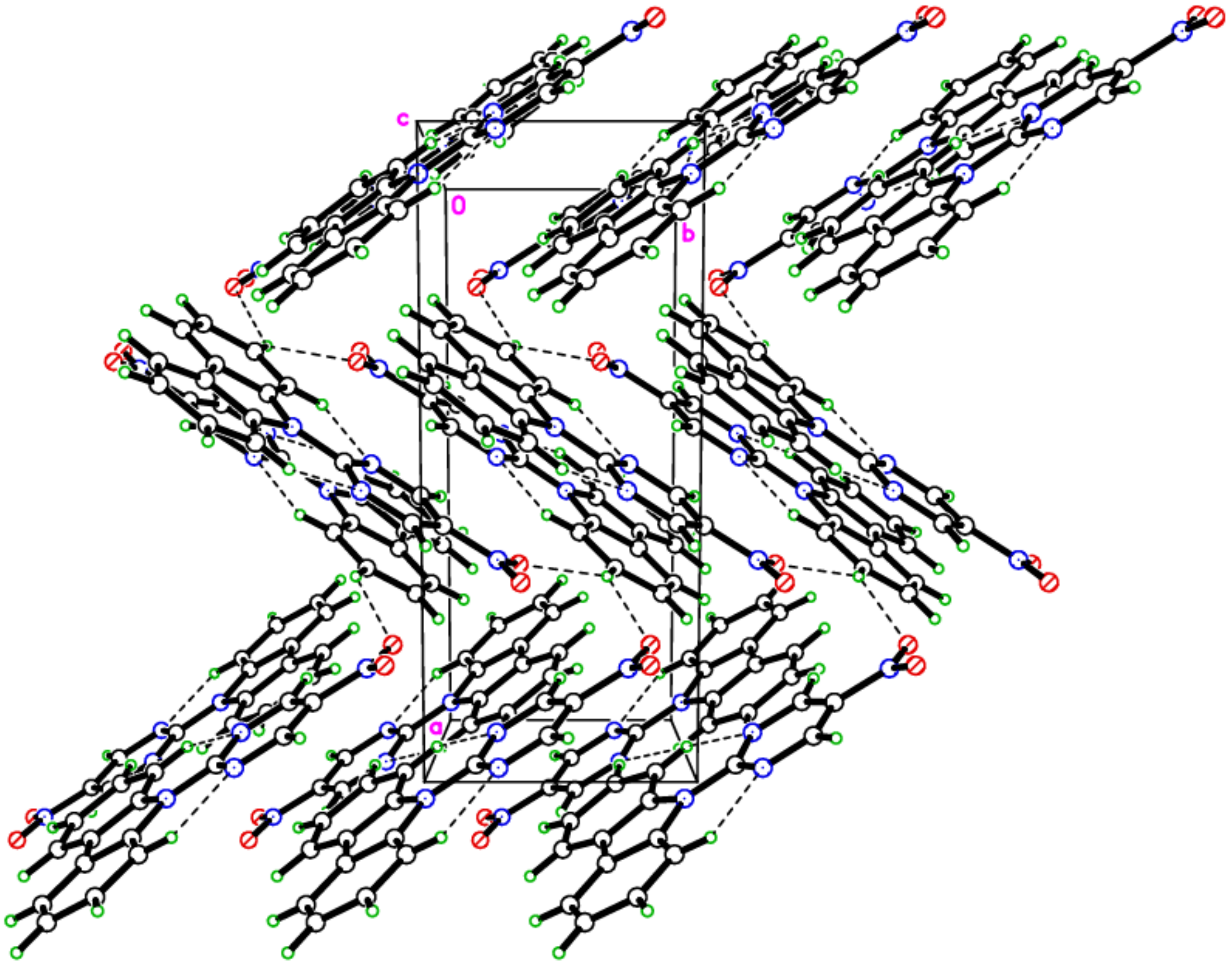

Figure S39 Crystal packing for **7** viewed down the *c*-axis. The IHB between the N atom of the pyrimidine and $H^{1/8}$ of the carbazole, and intermolecular interactions between the $–NO_2$ groups and carbazole $H^{2/7}$ atoms are shown as dashed lines.

Computational

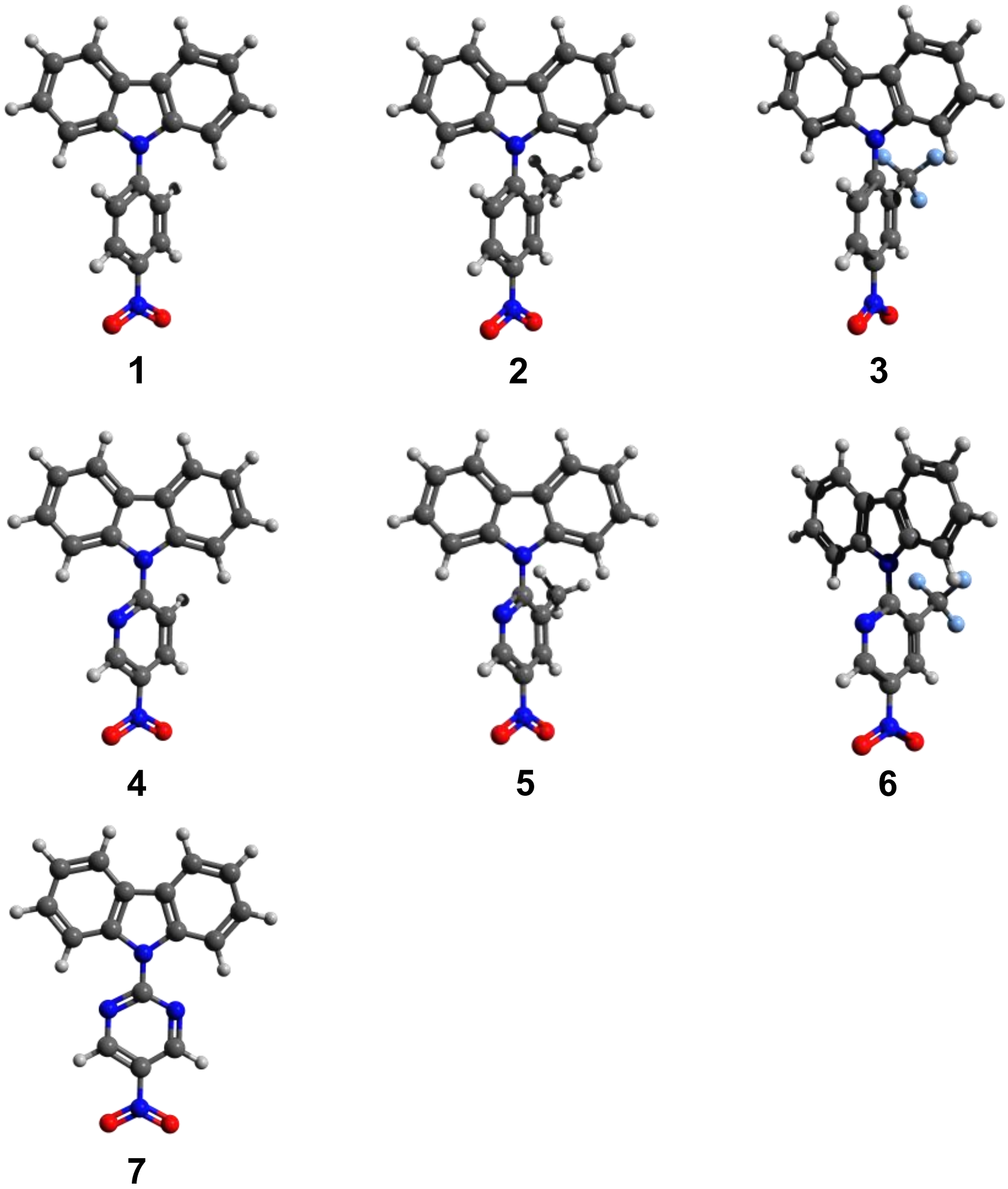

Figure S40 Optimised structures (*ab initio*, MP2/def2-SVP) for the S_0 geometry of **1**-**7**.

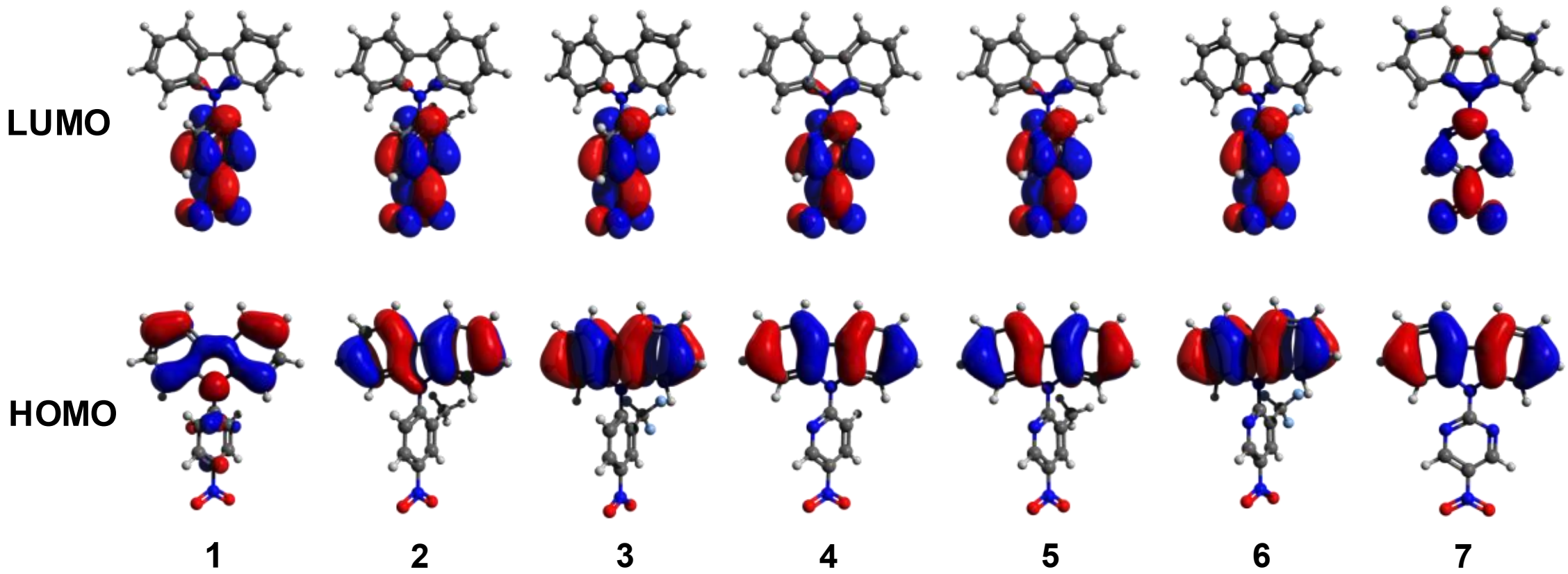

Figure S41 Highest occupied molecular orbital (HOMO) and lowest unoccupied molecular orbital (LUMO) distributions for **1**-**7** (*ab initio*, MP2/def2-SVP) in the S_0 state.

Photophysics

The absorption spectra were measured on a Shimadzu SolidSpec-3700 and the photoluminescence spectra were measured on a HORIBA Fluorolog-QM with excitation wavelength chosen to match the peak of the absorption band. The excitation wavelengths for each compound are shown in Table S3. Intensity of the photoluminescence spectra have been corrected to be a function of energy according to the Jacobian in Ref. S9.

Table S3: Excitation wavelengths used for the photoluminescence spectra in 1wt% PMMA and 20 μM solution (MCH = methylcyclohexane, 2-MeTHF = 2-methyltetrahydrofuran, MeCN = acetonitrile).

Compound	Excitation Wavelength (nm)				
	1 wt% PMMA	MCH	Toluene	2-MeTHF	MeCN
**1**	380	370	380	370	370
**2**	370	365	370	365	360
**3**	380	395	395	375	375
**4**	385	380	385	380	375
**5**	380	390	385	380	375
**6**	395	410	410	395	385
**7**	370	375	380	375	370

Table S4: Photoluminescence quantum yields of **1-7** in 1wt% PMMA.

Compound	PLQY (%)
**1**	1
**2**	<1
**3**	<1
**4**	2
**5**	<1
**6**	3
**7**	<1

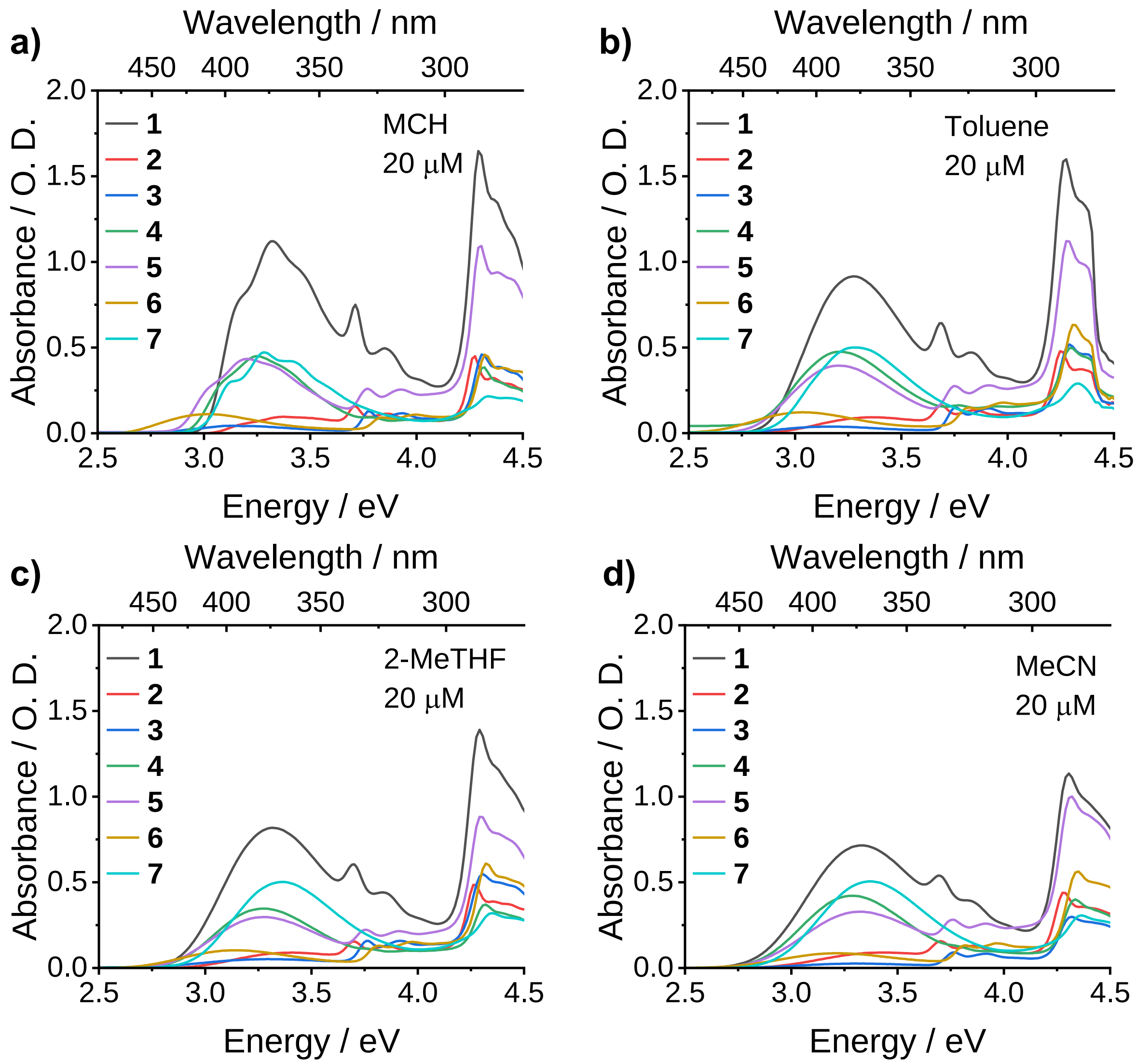

Figure S42 UV/vis absorbance spectra for **1-7** recorded at a molarity of 20 μM in a) methylcyclohexane (MCH), b) toluene, c) 2-methyltetrahydrofuran (2-MeTHF) d) acetonitrile (MeCN).

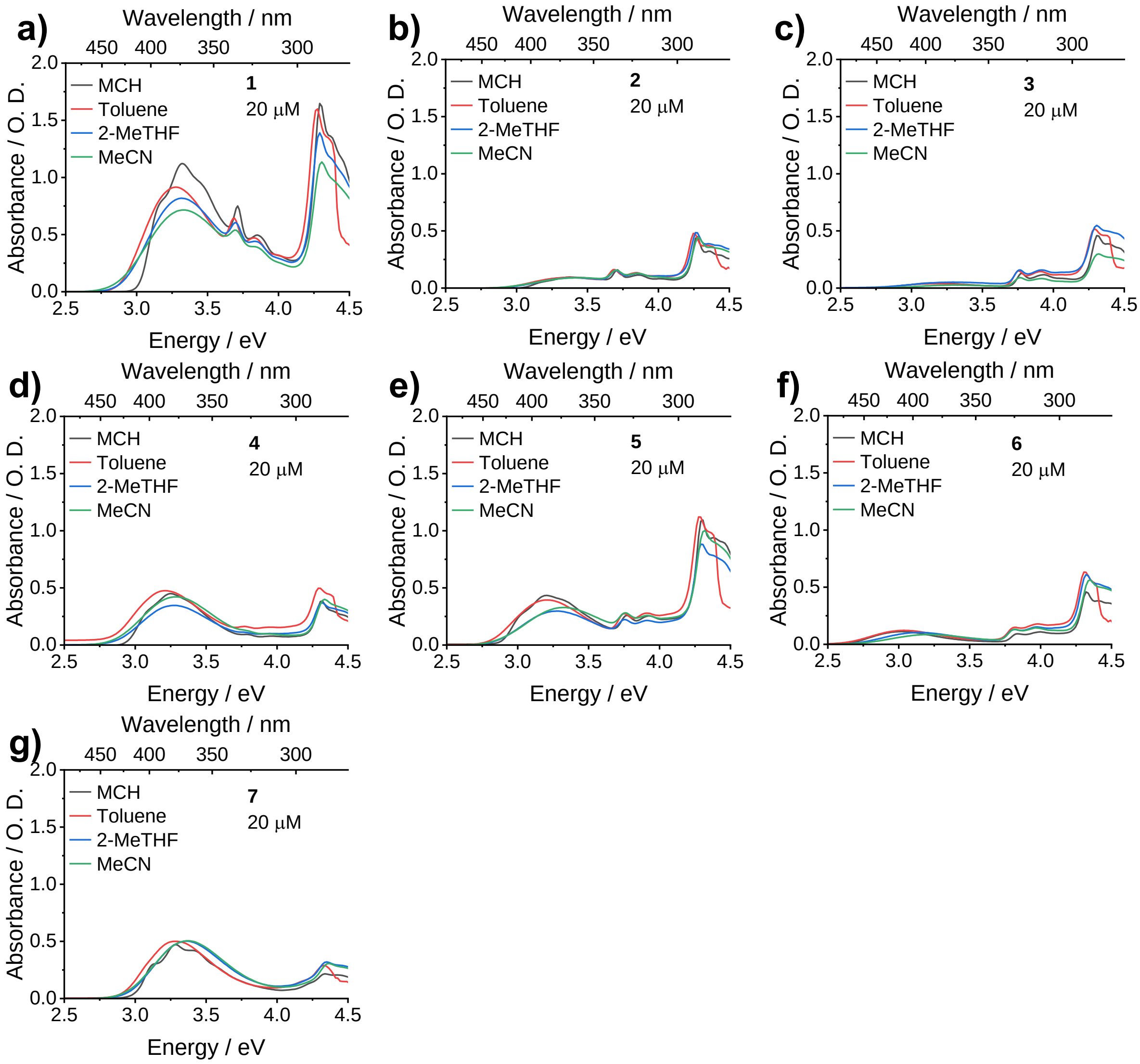

Figure S43 UV/vis absorbance spectra for **1-7** recorded at a molarity of 20 μM in a) methylcyclohexane (MCH), b) toluene, c) 2-methyltetrahydrofuran (2-MeTHF) d) acetonitrile (MeCN). This is analogous to Figure S42 but presented as individual plots for each molecule.

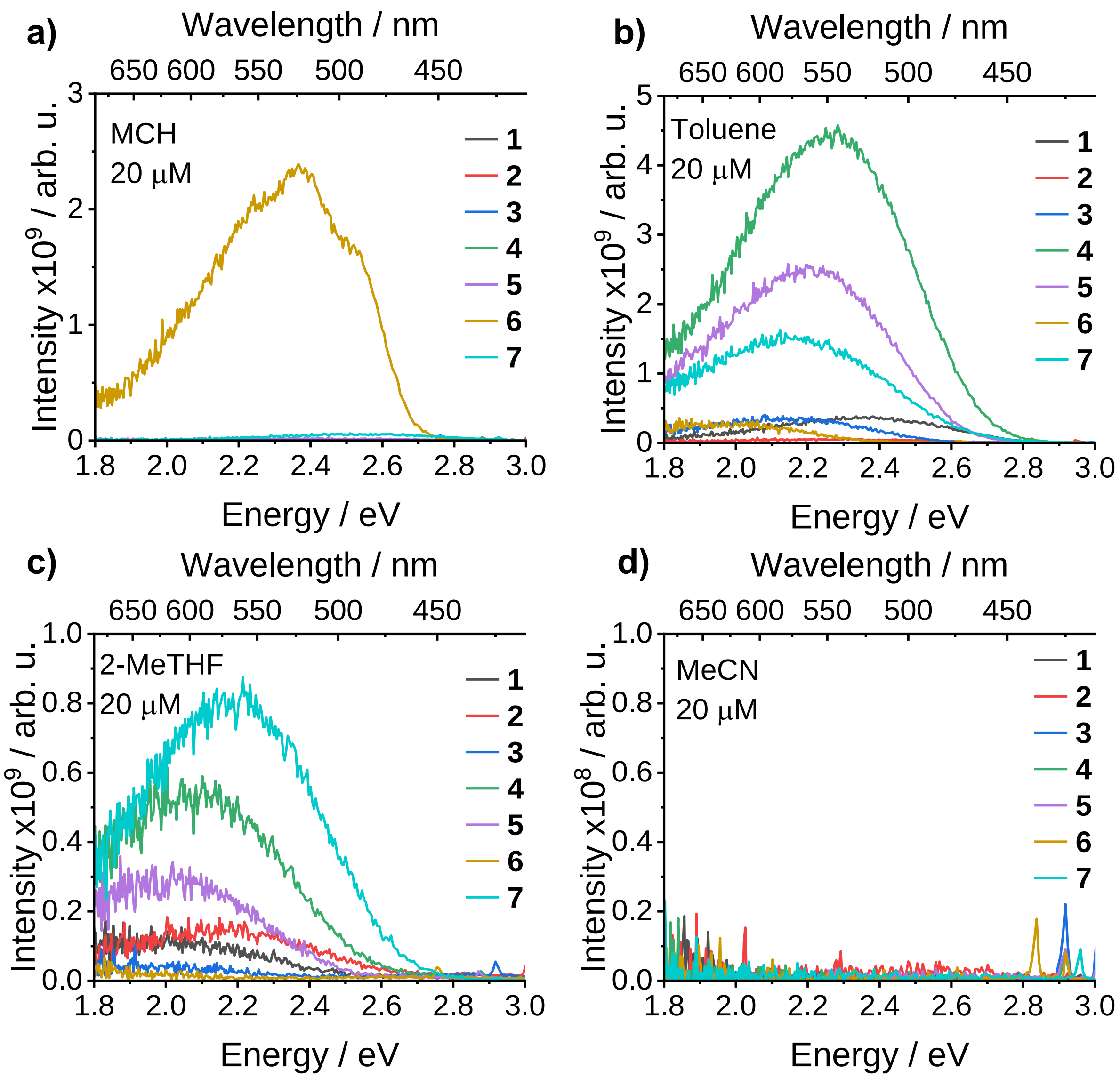

Figure S44 Photoluminescence spectra for **1**-**7** recorded at a molarity of 20 μM in a) methylcyclohexane (MCH), b) toluene, c) 2-methyltetrahydrofuran (2-MeTHF) d) acetonitrile (MeCN).

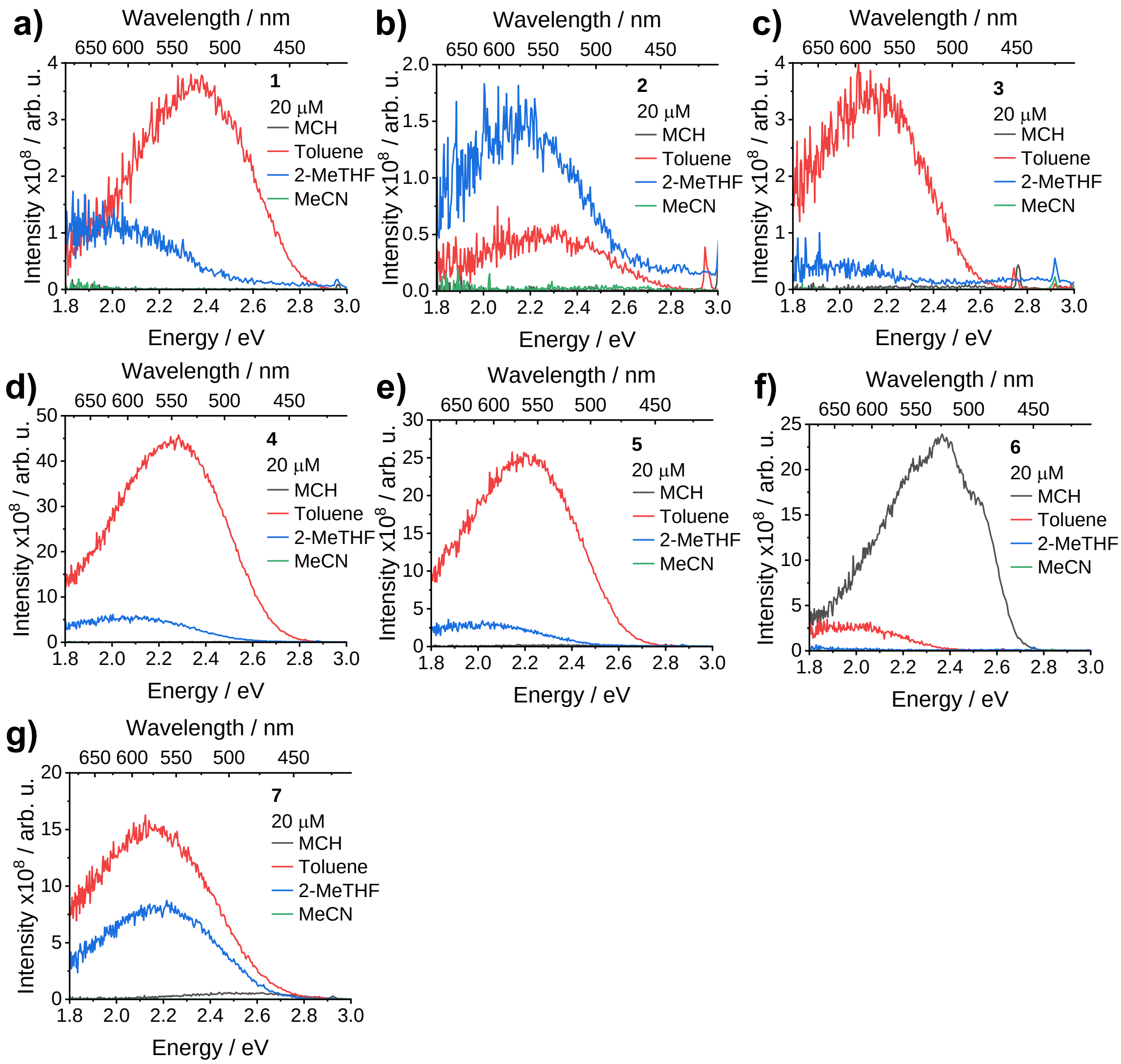

Figure S45 Photoluminescence spectra for **1**-**7** recorded at a molarity of 20 µM in a) methylcyclohexane (MCH), b) toluene, c) 2-methyltetrahydrofuran (2-MeTHF) d) acetonitrile (MeCN). This is analogous to Figure S44 but presented as individual plots for each molecule.

References

S1 Y.-C. Chen, G.-S. Huang, C.-C. Hsiao and S.-A. Chen, *J. Amer. Chem. Soc.*, 2006, **128**, 8549.

S2 Y. Liu, L. Pang, T. Liu, J. Guo, J. Wang and W. Li, *Dyes Pigments*, 2020, **173**, 107921.

S3 A. R. Katritzky, R. J. Offerman, J. M. Aurrecoechea and G. P. Savage, *Talanta*, 1990, **37**, 911.

S4 SAINT and APEX 2 (2008) software for CCD diffractometers. Bruker AXS Inc., Madison, USA.

S5 SADABS software, L. Krause, R. Herbst-Irmer, G.M. Sheldrick, & D. Stalke, *J. Appl. Cryst.* 2015,.**48**, 3

S6 *CrysAlis PRO* 1.171.40.45a software, Rigaku OD, 2019.

S7 G.M. Sheldrick, *Acta Cryst*. 2015, **A71,** 3.

S8 G.M. Sheldrick, *Acta Cryst*. 2015, **C71,** 3-8.

S9 J. Mooney and P. Kambhampati, *J. Phys. Chem. Lett.,* 2013, **4**, 3316.
